# STrengthening the Reporting Of Pharmacogenetic Studies: Development of the STROPS guideline

**DOI:** 10.1371/journal.pmed.1003344

**Published:** 2020-09-21

**Authors:** Marty Chaplin, Jamie J. Kirkham, Kerry Dwan, Derek J. Sloan, Geraint Davies, Andrea L. Jorgensen

**Affiliations:** 1 Department of Biostatistics, University of Liverpool, Liverpool, United Kingdom; 2 Centre for Biostatistics, Manchester Academic Health Science Centre, University of Manchester, Manchester, United Kingdom; 3 Cochrane Editorial Unit, London, United Kingdom; 4 School of Medicine, University of St Andrews, St Andrews, United Kingdom; 5 Department of Clinical Infection, Microbiology and Immunology, University of Liverpool, Liverpool, United Kingdom

## Abstract

**Background:**

Large sample sizes are often required to detect statistically significant associations between pharmacogenetic markers and treatment response. Meta-analysis may be performed to synthesize data from several studies, increasing sample size and, consequently, power to detect significant genetic effects. However, performing robust synthesis of data from pharmacogenetic studies is often challenging because of poor reporting of key data in study reports. There is currently no guideline for the reporting of pharmacogenetic studies that has been developed using a widely accepted robust methodology. The objective of this project was to develop the STrengthening the Reporting Of Pharmacogenetic Studies (STROPS) guideline.

**Methods and findings:**

We established a preliminary checklist of reporting items to be considered for inclusion in the guideline. We invited representatives of key stakeholder groups to participate in a 2-round Delphi survey. A total of 52 individuals participated in both rounds of the survey, scoring items with regards to their importance for inclusion in the STROPS guideline. We then held a consensus meeting, at which 8 individuals considered the results of the Delphi survey and voted on whether each item ought to be included in the final guideline. The STROPS guideline consists of 54 items and is accompanied by an explanation and elaboration document. The guideline contains items that are particularly important in the field of pharmacogenetics, such as the drug regimen of interest and whether adherence to treatment was accounted for in the conducted analyses. The guideline also requires that outcomes be clearly defined and justified, because in pharmacogenetic studies, there may be a greater number of possible outcomes than in other types of study (for example, disease–gene association studies). A limitation of this project is that our consensus meeting involved a small number of individuals, the majority of whom are based in the United Kingdom.

**Conclusions:**

Our aim is for the STROPS guideline to improve the transparency of reporting of pharmacogenetic studies and also to facilitate the conduct of high-quality systematic reviews and meta-analyses. We encourage authors to adhere to the STROPS guideline when publishing pharmacogenetic studies.

## Abstract 2

Marty Chaplin and co-authors recount the development of a guideline for reporting pharmacogenetic studies.

## Introduction

Pharmacogenetic studies investigate associations between genetic variants and treatment response for a particular drug in terms of both efficacy and adverse events. If a significant association between a genetic variant and a treatment response outcome is identified, patients may eventually be genotyped in clinical practice before being prescribed treatment. Healthcare providers may then refer to the genotyping test result when determining whether to prescribe the drug, and if prescribed, the appropriate drug dosage. This approach is known as “personalized medicine”.

Outcomes from pharmacogenetic studies are often complex traits; genetic influence may be explained by several genetic variants each having a small effect on outcome. Consequently, large sample sizes are typically required to detect pharmacogenetic associations. Meta-analysis improves sample size and increases power to detect significant associations while also allowing researchers to investigate the possibility that significant associations observed in individual studies may be spurious. However, authors may encounter difficulties when synthesizing evidence from pharmacogenetic studies because of poor reporting of data in study reports. For example, if study authors do not report outcomes for each genotype group separately, it may not be possible for researchers to include this study in meta-analyses. Furthermore, lack of reporting of participants’ ethnicities can also hinder investigations of heterogeneity, which form a key part of any systematic review and/or meta-analysis. Genetic associations often vary according to ethnicity; it is, therefore, recommended that meta-analyses are stratified by ethnicity, and pooling of results should only be performed if effect estimates for different ethnic groups appear sufficiently similar [1].

Although reporting guidelines are available for observational studies [2] and genetic association studies [3], to the best of our knowledge, no reporting guideline has been developed using rigorous methodologically specifically for pharmacogenetic studies. Pharmacogenetic studies have different characteristics than other types of observational and, indeed, genetic association studies. Although some items from existing guidelines can be applied to pharmacogenetic studies, there are many additional pharmacogenetic-specific characteristics that could be reported; clear guidance on which items are essential to report is needed.

In this article, we present results of a research project, the aim of which was to develop a reporting guideline for pharmacogenetic studies (the STrengthening the Reporting Of Pharmacogenetic Studies [STROPS] guideline) and an explanation and elaboration (E+E) document. Our aim is that the STROPS guideline will set a robust standard of reporting for pharmacogenetic studies and will consequently facilitate the conduct of high-quality systematic reviews and meta-analyses, thus improving power to detect pharmacogenetic associations.

## Methods

The protocol outlining the prespecified methods of this project has been published [4]. The 6 authors of this article form the steering committee for the project: Marty Chaplin (researcher into meta-analysis of pharmacogenetic studies), Jamie Kirkham (researcher into consensus methodology and developer of reporting guidelines), Kerry Dwan (researcher into systematic review methodology), Derek Sloan (clinical infectious disease researcher), Gerry Davies (clinical pharmacogenetic researcher in infectious diseases), and Andrea Jorgensen (researcher into statistical methods for pharmacogenetics, including evidence synthesis methods). In accordance with methodology proposed by Enhancing the QUAlity and Transparency Of health Research (EQUATOR) [4], we developed the STROPS guideline in the following stages: (1) development of a preliminary checklist, (2) two-round Delphi survey, (3) consensus meeting, and (4) development of the STROPS guideline and accompanying E+E document.

### Preliminary checklist of reporting items

To establish a preliminary checklist of reporting items, we first included items from existing relevant guidelines. We considered all guidelines listed on the EQUATOR website [5] under the clinical area of genetics. Two authors (MC and ALJ) assessed guidelines to be relevant if they were applicable to pharmacogenetics studies. Two authors (MC and ALJ) discussed whether items from these guidelines would ensure transparency of reporting of pharmacogenetic studies and consequently decided whether to include each item in the preliminary checklist. For example, the GRIPS statement [6] includes some items that can be applied to pharmacogenetic studies; however, we did not include all items from this guideline because many items are only relevant to studies in which a genetic risk prediction model is being developed, and these studies are outside the remit of our guideline. We modified some items from existing guidelines; the majority of these modifications were intended to make items more relevant to pharmacogenetic studies.

Second, we supplemented this list with additional items thought to be important. These items were either suggested by steering committee members based on our own experience in pharmacogenetic research or were drafted by MC and ALJ to cover issues identified by Jorgensen and Williamson [7], which relate specifically to the conduct of pharmacogenetic research. Finally, we drafted help text for each item, to ensure that language used was comprehensible to all Delphi participants. All steering committee members approved this preliminary checklist before the Delphi survey began.

### Delphi survey

#### Participants

In March 7, 2019–April 30, 2019, we invited 3 groups of stakeholders to participate in the Delphi survey. Stakeholder groups were chosen to encompass all aspects of pharmacogenetic research.

1. Primary researchers. We asked coordinators of 10 national and international pharmacogenetics networks (UK Pharmacogenetics and Stratified Medicine Network, Pharmacogenomics Research Network [PGRN], Canadian Pharmacogenomics Network for Drug Safety [CPNDS], South East Asian Pharmacogenomics Research Network [SEAPharm], Surveillance and Pharmacogenomics Initiative for Adverse Drug Reactions [SAPhIRE], Brazilian Pharmacogenetics Research Network [REFARGEN], European Society of Pharmacogenomics and Personalised Therapy [ESPT], European Federation for Pharmaceutical Sciences [EUFEPS] Network on Pharmacogenetics and Pharmacogenomics Research, and Clinical Pharmacogenetics Implementation Consortium [CPIC] and Ubiquitous Pharmacogenomics [U-PGx]) to forward the survey on to network members. We performed searches using Google to ensure that all major networks across the globe were identified.
2. Systematic reviewers. We identified 89 contact authors of systematic reviews of pharmacogenetics studies by searching PubMed, using the following search terms: “pharmacogenetics,” “pharmacogenomics,” “systematic review,” and “meta-analysis.” An information specialist designed the search strategy. We used a snowball technique, asking contact authors to complete the survey and to forward the survey on to their coauthors.
3. Journal editors. We contacted 210 editors-in-chief of 168 journals that may publish pharmacogenetic studies. We used a snowball technique, asking editors-in-chief to participate in the survey and also to forward the survey on to editors at their journal. We performed searches using Google to identify journals using search terms “pharmacogenetics,” “pharmacogenomics,” “precision medicine,” “personalised/personalized medicine,” and “journal.” We also considered journals listed on the “SCImago Journal & Country Rank” website [8] under the category “Genetics.”

#### Design

The Delphi process consisted of 2 rounds of survey, response and feedback. The first-round survey (Round 1, March 27, 2019–May 17, 2019) invited participants to score items from the preliminary list and to submit additional reporting items. The second-round survey (Round 2, May 31, 2019–July 12, 2019) provided feedback from the previous round and invited participants to rescore items. Additional reporting items submitted by participants in Round 1 (and approved by the steering committee) were included for scoring by participants in Round 2.

The Delphi survey was conducted using DelphiManager, a web-based system designed by the COMET Initiative (http://www.comet-initiative.org/delphimanager/) to facilitate the building and management of Delphi surveys.

#### Recruitment process

We e-mailed individuals from stakeholder groups with information about the STROPS project and Delphi process and an invitation to complete Round 1 within 3 weeks. We informed invitees that participation was optional and that scoring data would be anonymized; we allocated a unique identification number to each Delphi participant.

We sent a reminder e-mail at the end of the second week to prompt completion of the survey. All participants who completed Round 1 were invited to participate in Round 2. However, we informed invitees that completion of Round 1 did not necessitate completion in Round 2.

#### Ethics statement

The University of Liverpool Ethics Committee confirmed ethical approval for this study in January 2019 (Reference: 3586). We informed invitees to the Delphi survey that we would assume informed consent if an invitee responded to the survey.

#### Participant characteristics

We asked participants to provide their name, e-mail address, and their consent to be acknowledged as a Delphi participant in the published guideline.

#### Delphi scoring and consensus definition

Participants were asked to score each reporting item using a scale of 1–9, with 1–3 labeled “not important for inclusion in the guideline,” 4–6 labeled “important but not critical for inclusion in the guideline,” and 7–9 labeled “critical for inclusion into the guideline” [9]. Participants were also given the option to score a reporting item as “unable to score” if they were unable to offer an opinion on the importance of the item.

We defined that each stakeholder group had reached consensus for an item if at least 70% of members of that group scored the item as “critical for inclusion into the guideline.”

#### Delphi Round 1

Reporting items were presented in the order in which they would be addressed in the pharmacogenetic study report and were grouped under relevant headings: title and abstract, introduction, methods, results, discussion, and other information. Participants were asked to score each item as described previously and were also invited to suggest additional items for inclusion in the reporting guideline.

For each item, we summarized the number of respondents and the distribution of scores. Participants who scored an item as “unable to score” were excluded from the analysis for that particular item. We felt that this would not adversely affect our conclusions because for most items, the proportion of “unable to score” responses was minimal. Indeed, in Round 1, there was only 1 item for which more than 10% of participants responded “unable to score.”

The steering committee reviewed all additional reporting items suggested by participants. If items were not already covered by the existing list, we added these items to the list of reporting items presented in Round 2, or we covered the item as part of the E+E text for existing items.

#### Delphi Round 2

In Round 2, each participant was shown the number of respondents and distribution of scores for each item from Round 1, for each stakeholder group separately. Participants were also reminded how they personally scored each item in Round 1. Participants were asked to consider responses from other Delphi participants and to rescore the items. Additional items identified as part of Round 1 were scored by participants in Round 2.

For each item, the number of respondents and the distribution of scores were summarized. Participants who scored an item as “unable to score” were excluded from analysis for that particular item. Once again, for most items, the proportion of “unable to score” responses was minimal; there were 3 items for which more than 10% of participants responded “unable to score.”

If participants that did not respond to Round 2 have different opinions to participants from the same stakeholder group who completed both rounds, then Delphi results may be at risk of attrition bias. We investigated this risk by calculating average Round 1 scores for each participant and plotting these scores according to whether participants completed Round 2 or not for each stakeholder group. We visually examined these plots to assess the likelihood of attrition bias.

### Consensus meeting

The steering committee and stakeholder group representatives met to consider the Delphi results and to finalize the list of items for the reporting guideline. The meeting was conducted via conference call (Zoom). We aimed to include 1 or 2 representatives (with at least 1 being non-UK based) from each stakeholder group in the consensus meeting. We invited individuals to the meeting using the following principles: (1) Delphi participants who completed both rounds, (2) a balance across stakeholder groups, and (3) a reasonable geographic spread. If an individual could not attend, they were replaced by an individual from the same stakeholder group.

Prior to and during the meeting, attendees were shown a summary of how each stakeholder group scored each reporting item at Round 2 and the number of stakeholder groups who achieved consensus. Attendees discussed each reporting item in turn and decided whether to include the item in the reporting guideline or not. Where necessary, attendees voted using TurningPoint polling software (Turning Technologies, https://www.turningtechnologies.com/turningpoint/); the item was retained if at least 70% of participants voted for its inclusion. Items were considered in the order they were presented in the Delphi survey.

### Postconsensus meeting development

We drafted the initial reporting guideline and E+E document concurrently. The purpose of the E+E document is to provide the rationale for and meaning of each reporting item alongside examples of good reporting practice. We also provided the origin of each reporting item in the E+E document.

## Results

### Delphi survey

In Round 1, participants were asked to score 92 reporting items (the preliminary checklist of items) (S1 Table). The items are labeled 1 to 85, as some items have subitems, i.e., 52a, 52b, 52c. A total of 71 individuals completed this round: 15 journal editors, 41 primary researchers, and 15 systematic reviewers. A total of 10 participants suggested 31 additional reporting items. In addition, during Round 1, Delphi participants notified us of 2 publications containing relevant reporting items. After reviewing additional reporting items suggested by participants and the 2 relevant publications [10, 11], we included 7 additional items in Round 2 (S1 Table); we also covered some suggested reporting items by including additional detail in the E+E text for existing items.

A total of 52 individuals scored 99 reporting items in Round 2: 10 journal editors, 31 primary researchers, and 11 systematic reviewers. Anonymized data from both Delphi survey rounds are available in S1 Data. A list of individuals who gave their permission to be listed as participants in the Delphi survey is provided in S1 Document.

As we asked network coordinators, systematic reviewers, and journal editors to contact individuals on our behalf, it is impossible to determine a response rate to Round 1. However, we considered the response received to Round 2 to be reasonable (overall: 52/71, 73%; journal editors: 10/15, 67%; systematic reviewers: 31/41, 76%; primary researchers: 11/15, 73%). Considering the boxplots presented in S2 Document, the distributions of scores were similar between those who completed both rounds of the Delphi survey and those who completed Round 1 only. There was therefore no evidence to suggest that attrition bias occurred.

### Consensus meeting

The consensus meeting took place in November 7, 2019, and included 6 steering committee members and 4 representatives of stakeholder groups (1 journal editor, based in Germany; 1 primary researcher, based in Switzerland; 2 systematic reviewers, based in the UK and Spain). Names and affiliations of these representatives are provided in S1 Document. Two steering committee members did not participate in voting (JK chaired and KD took notes), so there were 8 voting individuals in attendance.

The consensus matrix (S2 Table) documents how each stakeholder group scored each item at Round 1 and at Round 2 and was provided to attendees prior to the meeting. Consensus meeting slides are provided in S1 Presentation.

Decisions made at the consensus meeting are summarized in S3 Table. We decided whether to include or exclude items and whether to combine multiple items under a single item. Where a vote was taken, this is indicated in the table; otherwise, decisions made were based solely on consideration of the Delphi results and discussion.

### Postconsensus meeting development

Following the consensus meeting, MC drafted the reporting guideline with guidance from the steering committee. The following minor amendments were made:

- We excluded item 14 and item 49; while searching for examples for these items, we found very few pharmacogenetic studies that used a matched cohort design or a cross-sectional design with a complex sampling strategy; these items would therefore be irrelevant to the vast majority of guideline users.
- We removed “Identify variables likely to be associated with population stratification (confounding by ethnic origin)” from item 22, because this is covered by item 54.
- We added more terms (“major,” “reference,” “risk,” and “effect”) that might be used to describe alleles to item 27.
- Although we decided to cover item 55 by adding to the E+E text for item 34 at the consensus meeting, the steering committee subsequently agreed that relatedness of participants is a separate issue to genotype quality control. We decided to keep item 55 as a standalone item in the guideline.
- We introduced a new subitem to item 42 to cover confounding and made item 42 a generic introduction to the statistical methods subitems.
- We modified item 68 to indicate that average and/or total follow-up time is sufficient.
- Although we voted to exclude item 80 from the “Other analyses” section of the reporting guideline at the consensus meeting, the intention was to consider this item under the “Databases” section. However, time constraints meant that we did not discuss this item again. The steering committee subsequently agreed that this item relates to additional results, rather than individual patient data in databases. We decided to keep the item in its original position, and add “i.e. in supplementary materials” so the meaning of the item is clear.

The resulting draft guideline was circulated to all consensus meeting attendees in March 2020. All comments and revisions were taken into consideration, and the checklist revised accordingly.

### STROPS guideline

In Table 1, we report the STROPS guideline. The accompanying E+E document is provided in S3 Document.

**Table 1:** STROPS reporting guideline.

Category	#	Criteria
**Abstract**		
Abstract	1	Provide in the abstract an informative and balanced summary of what was done and what was found.
**Introduction**		
Background/ rationale	2	Explain the scientific background and rationale for the investigation being reported.
	3	Provide reasons for choosing the genes and SNPs genotyped.
Objectives	4	State specific objectives, including any prespecified hypotheses.
	5	State if the study is the first report of a pharmacogenetic association, a replication effort, or both.
**Methods**		
Study design	6	Present key elements of study design early in the paper.
Setting	7	Describe the setting, locations, and relevant dates, including periods of recruitment, follow-up, and data collection.
Participants	8	Give the eligibility criteria and the sources and methods of selection of participants. For a cohort study, describe methods of follow-up. For a case-control study, state whether true controls or population controls were used. Give the rationale for the choice of cases and controls.
	9	Report the drug and regime participants were exposed to and the length of exposure.
	10	For a matched case-control study, give matching criteria and the number of controls per case.
	11	Give information on the criteria and methods for selection of subsets of participants from a larger study, when relevant.
	12	If other publications report results for the same patient cohort or a subset of the patient cohort, provide information on this patient cohort overlap and references to the relevant publications.
	13	Report disease/clinical indication of patients using a standardized ontology when possible.
Variables	14	Clearly define all outcomes, potential confounders, and effect modifiers. Give diagnostic criteria, if applicable.
	15	Provide justification for choice of outcomes.
	16	Clearly define genetic exposures (genetic variants) using a widely used nomenclature system.
	17	Report the rs number of each genotyped SNP.
	18	Clearly state how haplotypes or star alleles were defined.
	19	If referring to the minor, major, wild-type, mutant, reference, risk or effect allele of a variant, state which allele this is and for which given population/cohort.
Data sources/ measurement	20	For each variable of interest, give sources of data and details of methods of assessment (measurement). Describe comparability of assessment methods if there is more than one group.
	21	Describe laboratory methods, including source and storage of DNA, genotyping methods and platforms (including the allele calling algorithm used, and its version), error rates, and call rates. State the laboratory/center where genotyping was done. Describe comparability of laboratory methods if there is more than one group. Specify whether genotypes were assigned using all of the data from the study simultaneously or in smaller batches.
	22	Describe genotype quality control methods and findings.
	23	For quantitative outcome variables, specify if any investigation of potential bias resulting from pharmacotherapy was undertaken. If relevant, describe the nature and magnitude of the potential bias, and explain what approach was used to deal with this.
	24	Report how adherence to treatment was assessed, and report the results of the assessment.
Study size	25	Explain how the study size was arrived at, or provide details of the a priori power to detect effect sizes of varying degrees.
Quantitative variables	26	Explain how quantitative variables (confounders and effect modifiers) were handled in the analyses. If applicable, describe which groupings were chosen, and why.
Statistical methods	27	Address the following:
	(a)	Describe methods used to control for confounding.
	(b)	Describe any methods used to examine subgroups and interactions.
	(c)	Explain how missing data were addressed.
	(d)	Cohort study—If applicable, explain how loss to follow-up was addressed.
	(e)	Case-control study—If applicable, explain how matching of cases and controls was addressed.
	(f)	Describe any sensitivity analyses.
	28	State whether Hardy–Weinberg equilibrium was considered, and if so, how.
	29	Describe any methods used for inferring genotypes or haplotypes.
	30	Describe any methods used to assess or address population stratification.
	31	Describe any methods used to assess and correct for relatedness among subjects. Report results of assessments for relatedness.
	32	Describe any methods used to address multiple comparisons or to control risk of false positive results due to (a) multiple genetic variants, (b) multiple outcomes, and (c) multiple assumptions regarding mode of inheritance.
	33	Describe any methods used to adjust for extent of adherence in the analyses.
**Results**		
Participants	34	Report the numbers of individuals at each stage of the study—e.g., numbers potentially eligible, examined for eligibility, confirmed eligible, included in the study, completing follow-up, and analyzed.
SNPs	35	Report any SNPs that were excluded from analysis, and provide reasons for these exclusions.
Descriptive data	36	Give characteristics of study participants (e.g., demographic, clinical, social, ethnicity) and information on potential confounders.
	37	Cohort study—Summarize follow-up time, e.g., average and/or total amount.
	38	Where HWE tests have been undertaken, highlight SNPs that deviate from HWE.
	39	Where population stratification is assessed, report the results.
Outcome data	40a	For a cohort study, report all outcomes (phenotypes) investigated for each genotype category over time.
	40b	For a case-control study, report numbers in each genotype category for all outcomes investigated.
	40c	For a cross-sectional study, report all outcomes (phenotypes) investigated for each genotype category.
	41	If a study includes more than one ethnic group, provide the summary data specified in (40) per ethnic group.
Main results	42	Give unadjusted estimates, and if applicable, confounder-adjusted estimates and their precision (e.g., 95% confidence intervals). Make clear which confounders were adjusted for and why they were included.
	43	Report category boundaries when continuous variables were categorized.
Other analyses	44	Report other analyses done—e.g., analyses of subgroups and interactions, and sensitivity analyses.
	45	If numerous genetic exposures (genetic variants) were examined, summarize results from all analyses undertaken.
	46	If detailed results are available elsewhere, i.e., in supplementary materials, state how they can be accessed.
**Discussion**		
Key results	47	Summarize key results with reference to study objectives.
Limitations	48	Discuss limitations of the study, taking into account sources of potential bias or imprecision. Discuss both direction and magnitude of any potential bias.
Interpretation	49	Give a cautious overall interpretation of results considering objectives, limitations, multiplicity of analyses, results from similar studies, and other relevant evidence.
Generalizability	50	Discuss the generalizability (external validity) of the study results.
**Other information**		
Study registration	51	State whether the study has been registered. If the study has been registered, provide details of the registry.
Ethical approval	52	Report whether ethical approval was obtained for the collection of genetic data.
Funding	53	Give the source of funding and the role of the funders for the present study, and if applicable, for the original study on which the present article is based.
Databases	54	State whether databases for the analyzed data are or will become publicly available, and if so, how they can be accessed.

HWE, Hardy–Weinberg equilibrium; rs, reference SNP cluster ID; SNP, single nucleotide polymorphism.

## Discussion

The objective of this project was to develop the STROPS guideline. We used rigorous methodology for the development of reporting guidelines proposed by EQUATOR [4], including a 2-round Delphi survey and consensus meeting, both of which involved representatives of 3 key stakeholder groups. The final guideline consists of 54 items, 17 of which are novel items that have not been included in any existing guideline. A further 14 items originate from existing guidelines but have been modified for this pharmacogenetic-specific guideline. We encourage pharmacogenetic researchers to adhere to the STROPS guideline to ensure the transparency and completeness of their study reports.

Because of a lack of funding to cover travel and accommodation costs, we were unable to arrange a face-to-face consensus meeting as recommended by EQUATOR [4]. Our meeting was conducted via conference call, and the majority of meeting attendees were UK based. However, we invited a large, international and multidisciplinary cohort to participate in the Delphi survey, and meeting attendees were able to base their decisions on the opinions of this wider cohort. At the consensus meeting, we prioritized items for inclusion in the guideline if all stakeholder groups reached consensus, i.e., at least 70% of participants in each stakeholder group scored the item as “critical.” Although choice of threshold is subjective, prespecification of the threshold in the protocol ought to provide assurance that we did not define consensus in a post hoc way and therefore, that our own opinions did not bias the Delphi results [12].

The final phase of activities described by Moher and colleagues [4] relates to dissemination and implementation of the published guideline. We plan to circulate the STROPS guideline to individuals who completed both Delphi rounds and to ask coordinators of pharmacogenetic networks to notify their members of the publication of the guideline. We will also register the guideline on the EQUATOR website, present the guideline at conferences relevant to pharmacogenetic research, and seek guideline endorsement from relevant journals.

It is important to note that the STROPS guideline has been developed to improve the reporting of primary pharmacogenetic studies; to the best of our knowledge, no guideline exists for the reporting of systematic reviews and meta-analyses of pharmacogenetic studies. Evidence synthesis is an indispensable tool to researchers who are striving to improve the strength of the evidence base for pharmacogenetic associations, and a specific guideline designed to improve the reporting of systematic reviews and meta-analyses of pharmacogenetic studies would certainly be a useful addition in this field of research. Indeed, setting a robust standard for reporting of systematic reviews may improve the likelihood of pharmacogenetic findings being translated into clinical practice.

## Supporting information

S1 Table Items scored in the Delphi survey. (DOCX)

S2 Table Consensus matrix. (DOCX)

S3 Table Summary of decisions made at consensus meeting. (DOCX)

S1 Data Anonymized data from Round 1 and Round 2 of the Delphi survey. A score of “-9” indicates that the participant did not score an item or that the item was not scored because it was not available at Round 1. These items will also have a “0” in the Round column. Data from participants that only partially completed a round (i.e., did not score all items) were not included in or analyses. A score of “10” indicates that the participant selected “unable to score.” Participants who scored an item as “unable to score” were excluded from the analysis for that particular item.
(CSV)

S1 Document Delphi participants and consensus meeting attendees. (DOCX)

S2 Document Investigation of attrition bias in the Delphi survey. (DOCX)

S3 Document STROPS guideline: Explanation and elaboration document. STROPS, STrengthening the Reporting Of Pharmacogenetic Studies.
(DOCX)

S1 Presentation Consensus meeting slides. (PDF)

## Abbreviations

E+E: explanation and elaboration
EQUATOR: Enhancing the QUAlity and Transparency Of health Research
STROPS: STrengthening the Reporting Of Pharmacogenetic Studies
