# Supplementary material for: STrengthening the Reporting Of Pharmacogenetic Studies: Development of the STROPS guideline
Source: PLoS Med. 2020 Sep 21;17(9):e1003344. doi: 10.1371/journal.pmed.1003344 (PMC7505422; doi:10.1371/journal.pmed.1003344)
Supplement: S1 Table — (DOCX) [file pmed.1003344.s001.docx]

**S1 Table. Items scored in the Delphi survey**

| **Reporting item** | **Source** | **Help text** |
| --- | --- | --- |
| Reporting items scored at Round 1 and Round 2 | | |
| 1. Indicate the study’s pharmacogenetic design in the title and the abstract. | Modified from STROBE [1] (to be PGx-specific, and to specify that design should be indicated in title *and* abstract) | N/A |
| 2. Provide in the abstract an informative and balanced summary of what was done and what was found. | STROBE [1] | Provide the key information that enables readers to understand the research question, methods, results and conclusions of the study |
| 3. Explain the scientific background and rationale for the investigation being reported. | STROBE [1] | Provide the rationale for conducting the pharmacogenetic study in the context of existing research in this health area, i.e. what is known on a topic and what gaps in current knowledge are addressed by the study. |
| 4. Provide reasons for choosing the genes and SNPs genotyped. | Drafted by MC and ALJ to cover PGx-specific issue identified by Jorgensen and Williamson [2] | Explain how the investigated genes and SNPs were chosen, with reference to relevant functional/animal studies, previous association studies, and any procedures used such as the "tagging SNP" approach, or by assessing the likelihood of each individual SNP affecting the gene function with priority given to those with the most likely functional effect. |
| 5. If reasons for (4) include previous association studies, provide key details from these studies (effect size and standard error/confidence interval) | See above | N/A |
| 6. State specific objectives, including any pre-specified hypotheses. | STROBE [1] | Provide the objectives for the study, specifying the relevant population, genetic variants, drugs and outcomes. |
| 7. State if the study is the first report of a pharmacogenetic association, a replication effort, or both. | Modified from STREGA [3] (to be PGx-specific) | N/A |
| 8. Present key elements of study design early in the paper. | STROBE [1] | Present key elements of study design so that readers can understand the basics of the study, e.g. for a cohort study, state that the study used a cohort design, describe the group of people that comprised the cohort and the time period for which they were followed; for a case-control design, state that the study used a case-control design, describe the cases and controls and their source population, etc; for a post-hoc pharmacogenetic analysis of a RCT, state how the subjects included in the analysis were chosen, including which arm of the RCT they were from. |
| 9. Describe the setting, locations and relevant dates, including periods of recruitment, follow-up, and data collection. | Modified from STROBE [1] (removed "exposure" as not part of the setting in PGx studies) | Provide sufficient information to enable readers to assess the context and generalisability of a study's results. |
| 10. Cohort study – Give the eligibility criteria, and the sources and methods of selection of participants. Describe methods of follow-up. | STROBE [1] | Provide sufficiently detailed descriptions of the study participants to help readers understand the applicability of the results. Include details of follow-up procedures, including any procedures to minimise non-response/loss to follow-up. |
| 11. Case-control study – Give the eligibility criteria, and the sources and methods of case ascertainment and control selection. State whether true controls or population controls were used. Give the rationale for the choice of cases and controls. | Modified from STROBE [1] (added "state whether true controls or population controls were used") | Provide sufficiently detailed descriptions of cases and controls to help readers understand the applicability of the results. True controls are controls who have been exposed to the relevant treatment but have not developed the outcome of interest. Population controls are sometimes used in genetic studies for convenience; they are individuals who have already been genotyped that can be assumed to be controls, although we cannot ascertain whether they would have developed the outcome of interest if they had been exposed to the relevant treatment. |
| 12. Cross-sectional study – Give the eligibility criteria, and the sources and methods of selection of participants. | STROBE [1] | Provide sufficiently detailed descriptions of the study participants to help readers understand the applicability of the results. |
| 13. Report the drug and regime participants were exposed to, and the length of exposure. | Steering committee | Provide details of drug and regime, and length of exposure. This could be fixed across all participants, case/control specific, or variable. If variable, summarise in patient characteristics table. |
| 14. Cohort study – For matched studies, give matching criteria and number in each genotype group. | Modified from STROBE [1] (to be PGx-specific) | Provide details of variables that were used to match individuals from each genotype group at the start of follow-up to make groups more comparable, and the numbers in each group. |
| 15. Case-control study – For matched studies, give matching criteria and the number of controls per case. | STROBE [1] | Provide details of variables that were used to match cases and controls to ensure similarity between these groups, and the number of controls recruited per case. |
| 16. Give information on the criteria and methods for selection of subsets of participants from a larger study, when relevant. | STREGA [3] | If one or more sub-samples from a larger study are used for the investigation of a genetic association, provide details of: inclusion and exclusion criteria, sources and methods of selection for these sub-samples, and state whether these methods were pre-specified or post-hoc. |
| 17. If other publications report results for the same patient cohort, or a subset of the patient cohort, provide information on this patient cohort overlap and references to the relevant publications. | Steering committee | If other publications report results for the same patient cohort, or a subset of the patient cohort, report the numbers of patients in the current study for whom other publications report data for, rationale for the multiple publications, and provide references to the other publications. |
| 18. Provide justification for choice of outcomes. | Drafted by MC and ALJ to cover PGx-specific issue identified by Jorgensen and Williamson [2] | Explain why the outcomes are important, e.g. clinical importance, importance to patients, occurrence in previously developed core outcome sets, identification of a significant association in previous studies, etc. |
| 19. Clearly define all outcomes, potential confounders, and effect modifiers. Give diagnostic criteria, if applicable. | Modified from STROBE [1] (removed “predictors” as not relevant to PGx studies and “exposures” as genetic exposures are covered in (20) | Clearly define all outcomes, and all variables considered for and included in the analysis. Provide details of the diagnostic criteria for disease outcomes if applicable. |
| 20. Clearly define genetic exposures (genetic variants) using a widely-used nomenclature system. Identify variables likely to be associated with population stratification (confounding by ethnic origin). | STREGA [3] | The Human Gene Nomenclature Committee have published guidelines for human gene nomenclature (1, 2). Standard reference numbers are provided in dbSNP (3), the NCBI's database of genetic variation. Guidelines are available for variations not listed in dbSNP (4, 5).   Principle components analysis (PCA) can be undertaken to infer continuous axes of genetic variation that reduce the data to a small number of dimensions (6). The resulting principle components can be included as variables in analyses of association as a means of adjusting for population structure.  1. Wain HM, Bruford EA, Lovering RC, Lush MJ, Wright MW, Povey S. Guidelines for human gene nomenclature. Genomics. 2002;79(4):464-70. 2. Wain HM, Lush M, Ducluzeau F, Povey S. Genew: the human gene nomenclature database. Nucleic Acids Res. 2002;30(1):169-71. 3. Sherry ST, Ward M-H, Kholodov M, Baker J, Phan L, Smigielski EM, et al. dbSNP: the NCBI database of genetic variation. Nucleic Acids Res. 2001;29(1):308-11. 4. Antonarakis SE, Group NW. Recommendations for a nomenclature system for human gene mutations. Hum Mutat. 1998;11(1):1-3. 5. Dunnen JTd, Antonarakis SE. Mutation nomenclature extensions and suggestions to describe complex mutations: a discussion. Hum Mutat. 2000;15(1):7-12. 6. Price AL, Patterson NJ, Plenge RM, Weinblatt ME, Shadick NA, Reich D. Principal components analysis corrects for stratification in genome-wide association studies. Nat Genet. 2006;38(8):904. |
| 21. Report the rs number of each genotyped SNP. | Steering committee | An "rs" number (reference SNP ID number) is an identification tag assigned by NCBI to a group (or cluster) of SNPs that map to an identical location. The rs ID number, or rs tag, is assigned after submission of an SNP to dbSNP. |
| 22. Report whether the outcomes measured (including definitions) are in line with core/preferred outcome sets for the particular topic of interest | Steering committee | A core/preferred outcome set is an agreed standardised set of outcomes that should be measured and reported, as a minimum, in all clinical studies in specific areas of health or health care. The COMET database (1) lists references to planned, ongoing and completed core outcome set work for a wide range of health topics.   1. COMET. Core Outcome Measures in Effectiveness Trials [cited 2019 12 March]. Available from: http://www.comet-initiative.org/. |
| 23. For each variable of interest, give sources of data and details of methods of assessment (measurement). Describe comparability of assessment methods if there is more than one group. | STROBE [1] | Provide information on how all genetic exposures, confounders, and outcomes were measured. Report whether there were any differences in how data were collected in different patient groups (e.g. cases and controls). |
| 24. Describe laboratory methods, including source and storage of DNA, genotyping methods and platforms (including the allele calling algorithm used, and its version), error rates and call rates. State the laboratory/centre where genotyping was done. Describe comparability of laboratory methods if there is more than one group. Specify whether genotypes were assigned using all of the data from the study simultaneously or in smaller batches. | STREGA [3] | Provide sufficient details to enable the reader to assess the potential extent of genotyping errors (a source of information bias). Report whether there were any differences in laboratory methods in different patient groups (e.g. cases and controls). |
| 25. If study is case-control, confirm whether patients were genotyped in mixed batches. | Drafted by MC and ALJ to cover PGx-specific issue identified by Jorgensen and Williamson [2] | Report whether cases and controls were put into combined batches for genotyping purposes (to ensure genotyping quality is comparable across groups), rather than analysed in separate batches. |
| 26. Confirm whether genotyping personnel were blinded to outcome status | See above | N/A |
| 27. Describe the primers used. | See above | Report details of any primers that were used, or if primers were inherent to the assay used, state this and provide information on the assay. |
| 28. Describe genotype quality control methods. | See above | Provide details of genotype quality control methods, for example, using negative controls, or re-genotyping/re-sequencing in all or a random sample of patients. |
| 29. Describe findings of genotype quality control methods | See above | Provide sufficient information to enable readers to assess the risk of bias due to incorrect genotype allocation. |
| 30. Describe any efforts to address potential sources of bias. | STROBE [1] | Bias is a systematic deviation of a study’s result from a true value, due to flawed information or subject selection. Report all potential sources of bias, and any steps taken to reduce the likelihood of bias occurring. |
| 31. For quantitative outcome variables, specify if any investigation of potential bias resulting from pharmacotherapy was undertaken. If relevant, describe the nature and magnitude of the potential bias, and explain what approach was used to deal with this. | STREGA [3] | Bias from pharmacotherapy may occur when quantitative outcome variables are affected by treatment with drugs other than the study drug (e.g. outcome variables include biochemical markers of hepatotoxicity, and several patients are taking concomitant hepatotoxic medications). |
| 32. Report how adherence to treatment was assessed, and report the results of the assessment. | Drafted by MC and ALJ to cover PGx-specific issue identified by Jorgensen and Williamson [2] | Provide details on assessments of patient adherence, including limitations of the chosen method. |
| 33. Explain how the study size was arrived at, or provide details of the a priori power to detect effect sizes of varying degrees. | Modified from STROBE [1] (added “or provide details of the a priori power…”) | Report the calculation performed to obtain the study sample size, providing references to any specific methodology. Or, if sample size was predetermined (for example, secondary analyses of a published dataset), provide details of a priori power calculations for a range of plausible effect sizes. |
| 34. Explain how quantitative variables (confounders and effect modifiers) were handled in the analyses. If applicable, describe which groupings were chosen, and why. | Modified from STROBE [1] (added “confounders and effect modifiers”) | Explain how quantitative data (in relation to effect modifiers and confounders) were collected and analysed. Continuous variables may be grouped into categories to create a new categorical variable; explain why and how quantitative data were grouped. |
| 35. If applicable, describe how effects of treatment on quantitative outcome variables were dealt with. | Modified from STREGA [3] (added “on quantitative outcome variables”) | If any quantitative outcome variables may be affected by treatment with drugs other than the study drug, report whether any of the available methods for adjusting for treatment effects (1) were used to deal with this potential bias.   1. Tobin MD, Sheehan NA, Scurrah KJ, Burton PR. Adjusting for treatment effects in studies of quantitative traits: antihypertensive therapy and systolic blood pressure. Stat Med. 2005;24(19):2911-35. |
| 36. Describe all statistical methods, including those used to control for confounding. | STROBE [1] | Report which analyses were pre-specified and which were exploratory based on data inspection. If groups being compared are not similar regarding some characteristics, adjustment should be made for possible confounding variables. Provide details of procedures of variable selection and model comparison. |
| 37. State software version used and options (or settings) chosen. | STREGA [3] | Provide details of any specialized software/packages used to analyse the data. |
| 38. Describe any methods used to examine subgroups and interactions. | STROBE [1] | Explain what methods were used to examine whether associations differed across subgroups, or to examine interactions. An "interaction" occurs when one factor modifies the effect of another, and is also sometimes referred to as ‘effect modification'. Report whether these analyses were pre-planned or not. |
| 39. Explain how missing data were addressed. | STROBE [1] | Confirm whether analyses were restricted to individuals with complete data on the required variables, or whether any imputation of missing data was performed. |
| 40. Report any methods used to assess the assumption of missingness at random and the finding of such assessments. | Drafted by MC and ALJ to cover PGx-specific issue identified by Jorgensen and Williamson [2] | Data are said to be ‘missing at random’ if the fact that they are missing is unrelated to actual values of the missing data. Data that are missing at random may not be important. Analyses based on the available data will tend to be unbiased, although based on a smaller sample size than the original data set. |
| 41. Cohort study – If applicable, explain how loss to follow-up was addressed. | STROBE [1] | Individuals who withdrew from the study before the end of their observation period are "lost to follow-up". Report how many individuals were lost to follow-up, and whether these individuals were excluded or whether censoring strategies were used. Describe any censoring strategies. |
| 42. Case-control study – If applicable, explain how matching of cases and controls was addressed. | STROBE [1] | If a matched design was used, describe in detail what statistical methods were used to take into account the matching of cases and controls. |
| 43. Cross-sectional study – If applicable, describe analytical methods taking account of sampling strategy. | STROBE [1] | Sampling may be more complex than taking a simple random sample from the source population. For example, it may include several stages and clustering of participants. If a complex sampling strategy is used, estimates of association may be more or less precise than those derived from a simple random sample. If a complex sampling strategy was used, clearly state the methods used to adjust for this, so that readers may understand how the chosen sampling method influenced the precision of the obtained estimates. |
| 44. Describe any sensitivity analyses. | STROBE [1] | Provide details of any analyses that were performed to investigate whether the results of the main analysis are consistent with those obtained with alternative analysis strategies or assumptions. Report whether these analyses were pre-planned or not. |
| 45. State whether Hardy-Weinberg equilibrium was considered and, if so, how. | STREGA [3] | Describe any statistical tests or measures of departure from Hardy-Weinberg equilibrium, and any methods used to allow for deviations from Hardy-Weinberg equilibrium |
| 46. Where HWE test is undertaken, quote the p-value threshold applied to determine deviation from HWE. | Drafted by MC and ALJ to cover PGx-specific issue identified by Jorgensen and Williamson [2] | State how small a p-value from a HWE test had to be to indicate a statistically significant deviation from HWE |
| 47. Describe any methods used for inferring genotypes or haplotypes. | STREGA [3] | Provide statistical methods and software used to infer genotype phase and haplotypes. See the STREGA statement (1) for further information.   1. Little J, Higgins JP, Ioannidis JP, Moher D, Gagnon F, Von Elm E, et al. STrengthening the REporting of Genetic Association Studies (STREGA): an extension of the STROBE statement. Hum Genet. 2009;125(2):131-51. |
| 48. Describe any methods used to assess or address population stratification. | STREGA [3] | Explicitly document any methods used to assess the presence of population stratification or adjust for population stratification in the analyses. If no methods were used, state this. |
| 49. Describe any methods used to assess and correct for relatedness among subjects. Report results of assessments for relatedness | Modified from STREGA [3] (added “report results…”) | For pharmacogenetic studies, it is not uncommon for some participants to be related. Authors should report any methods used to assess relatedness, results of these assessments, and any methods used to correct for relatedness. |
| 50. Describe any assumptions made regarding mode of inheritance. | Drafted by MC and ALJ to cover PGx-specific issue identified by Jorgensen and Williamson [2] | The mode of inheritance for a particular SNP determines the observed trait for each given combination of parental alleles - some different modes of inheritance are summarised by Jorgensen and Williamson (1). If a particular mode of inheritance is assumed in the analyses, i.e. genotypes are grouped according to the resulting observed trait, state this. If more than one approach is used in the analysis, assuming different modes of inheritance, then report on all analyses performed.  1. Jorgensen AL, Williamson PR. Methodological quality of pharmacogenetic studies: issues of concern. Stat Med. 2008;27(30):6547-69. |
| 51. Provide justification for assumption of mode of inheritance or if no mode is assumed. | See above | For each analysis where a particular mode of inheritance is assumed, or no mode of inheritance is assumed, justify this approach, for example by summarising previous research on the mode of inheritance for the relevant SNP. If multiple analyses are performed making different assumptions, justify this decision. |
| 52a) Describe any methods used to address multiple comparisons or to control risk of false positive findings due to investigating multiple genetic variants. | Modified from STREGA [3] (to specify source of multiplicity) | Provide sufficient detail to enable the reader to assess the likelihood of false positive results (type 1 errors) being reported |
| 52b) Describe any methods used to address multiple comparisons or to control risk of false positive findings due to investigating multiple outcomes. | See above | Provide sufficient detail to enable the reader to assess the likelihood of false positive results (type 1 errors) being reported |
| 52c) Describe any methods used to address multiple comparisons or to control risk of false positive findings due to investigating multiple assumptions regarding mode of inheritance. | See above | Provide sufficient detail to enable the reader to assess the likelihood of false positive results (type 1 errors) being reported |
| 53. Describe any methods used to adjust for extent of adherence in the analyses. | Drafted by MC and ALJ to cover PGx-specific issue identified by Jorgensen and Williamson [2] | N/A |
| 54a) Report the numbers of individuals at each stage of the study – e.g., numbers potentially eligible, examined for eligibility, confirmed eligible, included in the study, completing follow-up, and analysed. | STROBE [1] | Give an account of the numbers of individuals considered at each stage of recruiting study participants, from the choice of a target population to the inclusion of participants' data in the analysis. |
| 54b) Give reasons for non-participation at each stage. | STROBE [1] | Provide transparent information on reasons for non-participation/exclusion of participants at each stage, to allow the reader to judge whether the study population was representative of the target population, and whether bias was possibly introduced. |
| 54c) Consider use of a flow diagram. | STROBE [1] | A flow diagram can be an efficient and transparent way to convey the information described in 54a) and 54b), which may otherwise require a lengthy description in the text. |
| 55. For each genetic variant, report numbers of individuals in whom genotyping was attempted and numbers of individuals in whom genotyping was successful. | Modified from STREGA [3] (added “for each genetic variant”) | N/A |
| 56. Report any SNPs that were excluded from analysis, and provide reasons for these exclusions | Drafted by MC and ALJ to cover PGx-specific issue identified by Jorgensen and Williamson [2] | Examples of possible reasons for excluding SNPs are the failure of the HWE test, or excessive missing data, etc. |
| 57. Give characteristics of study participants (e.g., demographic, clinical, social) and information on potential confounders. | Modified from STROBE [1] (removed “exposures”) | Report participant characteristics with appropriate summary measures. For example, for continuous data, mean and standard deviation, or median and range; for dichotomous data, numbers and proportions. |
| 58. Indicate the number of participants with missing data for each variable of interest. | STROBE [1] | Report the amount of missing genotype data, missing data for potential confounders, and missing data for other important patient characteristics and outcomes. |
| 59. For a cohort study, consider giving information listed in (57) and (58) by genotype. | Modified from STREGA [3] (added “for a cohort study”) | Where several genetic variants have been considered, this may not be practical. |
| 60. For a case-control study, give the information listed in (57) and (58) for cases and controls separately. | Steering committee | N/A |
| 61. Report reasons for missing genotype data. | Drafted by MC and ALJ to cover PGx-specific issue identified by Jorgensen and Williamson [2] | Report any reasons that genotype data were missing and report how much of the missing data was attributed to each reason |
| 62. Cohort study – Summarize follow-up time, e.g. average and total amount. | STROBE [1] | Average follow-up can be summarised using the mean and/or median follow-up time. Total amount of follow-up may be reported using total person-years of follow-up, or some indication of the completeness of follow-up (1).   1. Clark TG, Altman DG, De Stavola BL. Quantification of the completeness of follow-up. Lancet. 2002;359(9314):1309-10. |
| 63. Where HWE test undertaken, highlight SNPs that deviate from HWE. | Drafted by MC and ALJ to cover PGx-specific issue identified by Jorgensen and Williamson [2] | Report any SNPs for which a HWE test indicated deviation for HWE. |
| 64. Where population stratification is assessed, report the results. | See above | Report the results of any tests performed to detect the presence of population stratification. |
| 65a) For a cohort study, report all outcomes (phenotypes) investigated for each genotype category over time | Modified from STREGA [3] (clarified that all investigated outcomes should be reported) | For outcomes that relate to the occurrence of some event, report the number of events that occurred. If the risk of an event occurring changes over follow-up time, present the numbers and rates of events in appropriate intervals of follow-up or as a Kaplan-Meier life table or plot. For other outcomes, present appropriate summary measures (e.g. means and standard deviations) over time. |
| 65b) For a case-control study, report numbers in each genotype category for all outcomes investigated | See above | Report numbers of cases and controls in each genotype category. |
| 65c) For a cross sectional study, report all outcomes (phenotypes) investigated for each genotype category | See above | For outcomes that relate to the occurrence of some event, report the number of events that occurred. For other outcomes, present appropriate summary measures (e.g. means and standard deviations). |
| 66. If a study includes more than one ethnic group, provide the summary data specified in (65) per ethnic group. | Steering committee | If the study includes patients belonging to different ethnic groups, provide all outcome data stratified by ethnicity. |
| 67. Give unadjusted estimates and, if applicable, confounder-adjusted estimates and their precision (e.g., 95% confidence intervals). Make clear which confounders were adjusted for and why they were included. | STROBE [1] | Provide both unadjusted measures of association and measures of association adjusted for potential confounders, to enable readers to compare both measures and assess how the measure of association is impacted by adjusting for confounders. List all potential confounder variables considered, and the criteria/rationale for excluding or including variables in statistical models. |
| 68. Report category boundaries when continuous variables were categorized. | STROBE [1] | If continuous outcomes were categorised, report the range of values covered by each category. |
| 69. If relevant, consider translating effect estimates to number needed to test to illustrate potential clinical utility of any significant findings. | Modified from STROBE [1] (to be PGx-specific) | In pharmacogenetic studies, a "number needed to test" (NNT) can be calculated to demonstrate the clinical relevance of study findings. For example, "12 tuberculosis patients (95% CI: 7 to 23) would need to be tested for the genetic variant (and possibly put on alternative therapy dependent on the results of the test) to prevent one case of hepatotoxicity" |
| 70. Report results of any adjustments for multiple comparisons. | STREGA [3] | For example, report Bonferroni adjusted p-values, or false discovery rates. |
| 71. Report precise p-values for all associations. | Drafted by MC and ALJ to cover PGx-specific issue identified by Jorgensen and Williamson [2] | Report the precise p-values for all associations, as opposed to only indicating whether an association was found to be statistically significant or not. For example, stating p<0.05 or p>0.05, or indicating statistical significance (or a lack of) by using asterisks (*) is not sufficient to satisfy this criterion. Reporting of p-values may be subject to journal guidelines; p-values below a certain threshold e.g. p<0.0001 may need to be reported as such. |
| 72. Report other analyses done – e.g., analyses of subgroups and interactions, and sensitivity analyses. | STROBE [1] | Report the results of any analyses performed in addition to the main analysis. It may be impractical to present detailed findings for all analyses performed; in this case, present detailed results for important results only. Less important results can be summarised briefly in the text i.e. "results of the sensitivity analysis were consistent with the results of the main analysis" and detailed in full in supplementary materials. |
| 73. If numerous genetic exposures (genetic variants) were examined, summarize results from all analyses undertaken. | STREGA [3] | Report results for all genetic variants that were investigated in the study, rather than selectively reporting only "interesting" or significant results. Full results can be provided in supplementary materials if necessary. |
| 74. If detailed results are available elsewhere, state how they can be accessed. | STREGA [3] | Report what results are available, and where to find these results. Provide sufficient details that a reader would easily be able to locate these resources. |
| 75. Summarize key results with reference to study objectives. | STROBE [1] | Remind the reader of the main findings of the study with a short summary. This helps the reader to assess whether the author's interpretation and suggested implications are supported by the findings. |
| 76. Discuss limitations of the study, taking into account sources of potential bias or imprecision. Discuss both direction and magnitude of any potential bias. | STROBE [1] | Help the reader to interpret the validity and health care relevance of the study findings. Limitations might relate to, for example, characteristics of included patients, methods of outcome measurement, multiplicity of analyses, missing data, etc. |
| 77. Give a cautious overall interpretation of results considering objectives, limitations, multiplicity of analyses, results from similar studies, and other relevant evidence. | STROBE [1] | When interpreting results, authors should consider the position of the study on the discovery to verification continuum. Consider potential sources of bias, residual confounding (due to unmeasured variables or imprecise measurement of confounders), the results of relevant sensitivity analyses, the issue of multiplicity and subgroup analyses. Authors should address the real range of uncertainty in estimates, which is larger than the statistical uncertainty reflected in confidence intervals. |
| 78. Report genotype frequencies from other studies | Drafted by MC and ALJ to cover PGx-specific issue identified by Jorgensen and Williamson [2] | If allelic frequencies have previously been reported for individuals from the same population, quote these for comparison purposes. |
| 79. Discuss the generalisability (external validity) of the study results. | STROBE [1] | Consider the extent to which the results of the study can be applied to other circumstances, i.e. different populations/settings/countries. |
| 80. Discuss, if pertinent, the health care relevance of the study results. | Modified from GRIPS [4] (removed “generalizability” as covered in previous item) | Consider 1) the efforts it takes to obtain the additional genotype information, 2) the impact the results might have on medical or public health decision making and on expected health benefits, and 3) the extent to which these benefits will outweigh the potential harms related to genetic testing and the cost of implementation. Describe what evidence is still needed before health care implementation can be considered. |
| 81. State whether the protocol for the analysed data is publicly available and if so, how the protocol can be accessed. | Steering committee | If the protocol is available, provide sufficient details that a reader would easily be able to locate this resource. |
| 82. State whether the study has been registered. If the study has been registered, provide details of the registry. | Steering committee | Studies can be registered on many different official platforms; the most widely used platform is ClinicalTrials.gov. |
| 83a) Report whether ethical approval was obtained for the collection of genetic data | Steering committee | N/A |
| 83b) If ethical approval was obtained, report the committee that gave ethical approval and a reference ID | Steering committee | N/A |
| 84. Give the source of funding and the role of the funders for the present study and, if applicable, for the original study on which the present article is based. | STROBE [1] | Role of funders: State which part of the study the funders took direct responsibility for, e.g., study design, data collection, analysis, drafting of manuscript, decision to publish. |
| 85. State whether databases for the analysed data are or will become publicly available and if so, how they can be accessed. | Modified from GRIPS [4] (removed reference to risk models) | If databases are available, provide sufficient details that a reader would easily be able to locate these resources. |
| Additional reporting items suggested by Delphi participants, scored in Round 2 only | | |
| Clearly state how haplotypes or star alleles were defined | Delphi Round 1 | Even when referring to well-defined star alleles or haplotypes, it is good practice to provide full details in the current publication using standard nomenclature. For example, “NAT2*5A was defined as rs1801280 c.341 allele C and rs1799929 c.481 allele T”. |
| Clearly state on which chromosomal strand the alleles are reported | Delphi Round 1 | If the chromosomal strand for which alleles are reported is not stated, it is difficult to know which allele is associated with the phenotype of interest for A/T or G/C SNPs. A statement such as ‘the A allele (positive chromosomal strand) is associated with...’ is clear. |
| If studying drug metabolites, provide references and links to structures and database identifiers | Delphi Round 1 | e.g. PubChem Compound IDs (https://pubchem.ncbi.nlm.nih.gov/) |
| Report disease/clinical indication of patients using a standardised ontology | Delphi Round 1 | e.g. SNOMED CT; Mesh |
| If referring to the minor, wild-type or mutant allele of a variant, state which allele this is and for which given population/cohort. | Delphi Round 1 | The minor (less frequent) allele in one population may be the major (more frequent) allele in a different population. The allele and population should be clearly stated if using the terms ‘minor’, ‘wild-type’ or ‘mutant’, e.g. ‘the minor allele, T, in Gujarati Indians’. |
| Report on the risk of phenoconversion (genotype-phenotype mismatch) and its magnitude in the study population | Delphi Round 1 | For more information, see:  Shah RR, Smith RL. Addressing phenoconversion: the Achilles' heel of personalized medicine. British journal of clinical pharmacology. 2015 Feb 1;79(2):222-40. |
| Confirm whether patients were blinded to their genotyping result | Delphi Round 1 | N/A |

References

1. Von Elm E, Altman DG, Egger M, Pocock SJ, Gøtzsche PC, Vandenbroucke JP. The Strengthening the Reporting of Observational Studies in Epidemiology (STROBE) statement: guidelines for reporting observational studies. Ann Intern Med. 2007;147(8): 573-577.

2. Jorgensen AL, Williamson PR. Methodological quality of pharmacogenetic studies: issues of concern. Stat Med. 2008;27(30): 6547-6569.

3. Little J, Higgins JP, Ioannidis JP, Moher D, Gagnon F, Von Elm E, et al. STrengthening the REporting of Genetic Association studies (STREGA): an extension of the STROBE statement. Hum Genet. 2009;125(2): 131-151.

4. Janssens AC, Ioannidis JP, van Duijn CM, Little J, Khoury MJ. Strengthening the reporting of Genetic RIsk Prediction Studies: the GRIPS Statement. PLoS Med. 2011;8(3): e1000420.
