# Supplementary material for: STrengthening the Reporting Of Pharmacogenetic Studies: Development of the STROPS guideline
Source: PLoS Med. 2020 Sep 21;17(9):e1003344. doi: 10.1371/journal.pmed.1003344 (PMC7505422; doi:10.1371/journal.pmed.1003344)
Supplement: S3 Table — (DOCX) [file pmed.1003344.s003.docx]

S3 Table. Summary of decisions made at consensus meeting

| **Category** | **#** | **Criteria** | **Consensus at Delphi** | **Decision at consensus meeting** |
| --- | --- | --- | --- | --- |
| **Title and abstract** | | | | |
| Title and abstract | 1 | Indicate the study’s pharmacogenetic design in the title and the abstract. | 1 group | Exclude after vote |
|  | 2 | Provide in the abstract an informative and balanced summary of what was done and what was found. | 3 groups | Include - add to E+E text that study design should be reported in abstract |
| **Introduction** | | | | |
| Background/  rationale | 3 | Explain the scientific background and rationale for the investigation being reported. | 3 groups | Include |
|  | 4 | Provide reasons for choosing the genes and SNPs genotyped. | 3 groups | Include |
|  | 5 | If reasons for (4) include previous association studies, provide key details from these studies (effect size and standard error/confidence interval). | 1 group | Exclude after vote |
| Objectives | 6 | State specific objectives, including any pre-specified hypotheses. | 3 groups | Include |
|  | 7 | State if the study is the first report of a pharmacogenetic association, a replication effort, or both. | 2 groups | Include after vote |
| **Methods** | | | | |
| Study design | 8 | Present key elements of study design early in the paper. | 3 groups | Include |
| Setting | 9 | Describe the setting, locations and relevant dates, including periods of recruitment, follow-up, and data collection. | 0 groups | Include after vote |
| Participants | 10 | Cohort study – Give the eligibility criteria, and the sources and methods of selection of participants. Describe methods of follow-up. | 3 groups | Combine #10, #11 and #12 |
|  | 11 | Case-control study – Give the eligibility criteria, and the sources and methods of case ascertainment and control selection. State whether true controls or population controls were used. Give the rationale for the choice of cases and controls. | 3 groups | Combine #10, #11 and #12 |
|  | 12 | Cross-sectional study – Give the eligibility criteria, and the sources and methods of selection of participants. | 3 groups | Combine #10, #11 and #12 |
|  | 13 | Report the drug and regime participants were exposed to, and the length of exposure. | 3 groups | Include |
|  | 14 | Cohort study – For matched studies, give matching criteria and number in each genotype group. | 3 groups | Combine #14, #15 |
|  | 15 | Case-control study – For matched studies, give matching criteria and the number of controls per case. | 3 groups | Combine #14, #15 |
|  | 16 | Give information on the criteria and methods for selection of subsets of participants from a larger study, when relevant. | 3 groups | Include |
|  | 17 | If other publications report results for the same patient cohort, or a subset of the patient cohort, provide information on this patient cohort overlap and references to the relevant publications. | 2 groups | Include after vote |
|  | 18 | Report disease/clinical indication of patients using a standardised ontology. | 1 group | Include and add “where possible” to E+E text (standardised ontology may not always be available); vote taken on whether to include the item with the change to E+E text |
|  | 19 | Confirm whether patients were blinded to their genotyping result. | 1 group | Exclude after vote |
| Variables | 20 | Provide justification for choice of outcomes. | 3 groups | Include; reverse order of #20 and #21 |
|  | 21 | Clearly define all outcomes, potential confounders, and effect modifiers. Give diagnostic criteria, if applicable. | 3 groups | Include; reverse order of #20 and #21 |
|  | 22 | Clearly define genetic exposures (genetic variants) using a widely-used nomenclature system. Identify variables likely to be associated with population stratification (confounding by ethnic origin). | 3 groups | Include |
|  | 23 | Report the rs number of each genotyped SNP. | 3 groups | Include |
|  | 24 | Report whether the outcomes measured (including definitions) are in line with core/preferred outcome sets for the particular topic of interest. | 1 group | Exclude |
|  | 25 | Clearly state how haplotypes or star alleles were defined. | 3 groups | Include |
|  | 26 | Clearly state on which chromosomal strand the alleles are reported. | 1 group | Exclude |
|  | 27 | If referring to the minor, wild-type or mutant allele of a variant, state which allele this is and for which given population/cohort. | 2 groups | Include after vote |
|  | 28 | If studying drug metabolites; provide references and links to structures and database identifiers. | 0 groups | Exclude |
| Data sources /measurement | 29 | For each variable of interest, give sources of data and details of methods of assessment (measurement). Describe comparability of assessment methods if there is more than one group. | 1 group | Include after vote |
|  | 30 | Describe laboratory methods, including source and storage of DNA, genotyping methods and *platforms* (including the allele calling algorithm used, and its version), error rates and call rates. State the laboratory/centre where genotyping was done. Describe comparability of laboratory methods if there is more than one group. Specify whether genotypes were assigned using all of the data from the study simultaneously or in smaller batches. | 3 groups | Include |
|  | 31 | If study is case-control, confirm whether patients were genotyped in mixed batches. | 0 groups | Cover by adding to E+E text for #30 |
|  | 32 | Confirm whether genotyping personnel were blinded to outcome status. | 0 groups | Exclude |
|  | 33 | Describe the primers used. | 0 groups | Exclude |
|  | 34 | Describe genotype quality control methods. | 1 group | Include after vote |
|  | 35 | Describe findings of genotype quality control methods. | 0 groups | Combine with #34 |
| Bias | 36 | Describe any efforts to address potential sources of bias. | 1 group | Exclude after vote |
|  | 37 | For quantitative outcome variables, specify if any investigation of potential bias resulting from pharmacotherapy was undertaken. If relevant, describe the nature and magnitude of the potential bias, and explain what approach was used to deal with this. | 3 groups | Include |
|  | 38 | Report how adherence to treatment was assessed, and report the results of the assessment. | 3 groups | Include |
| Study size | 39 | Explain how the study size was arrived at, or provide details of the a priori power to detect effect sizes of varying degrees. | 3 groups | Include |
| Quantitative variables | 40 | Explain how quantitative variables (confounders and effect modifiers) were handled in the analyses. If applicable, describe which groupings were chosen, and why. | 3 groups | Include |
|  | 41 | If applicable, describe how effects of treatment on quantitative outcome variables were dealt with. | 2 groups | Exclude |
| Statistical methods | 42 | Describe all statistical methods, including those used to control for confounding. | 3 groups | Include |
|  | 43 | State software version used and options (or settings) chosen. | 0 groups | Exclude |
|  | 44 | Describe any methods used to examine subgroups and interactions. | 1 group | Include after vote as subitem to #42 |
|  | 45 | Explain how missing data were addressed. | 3 groups | Include as subitem to #42 |
|  | 46 | Report any methods used to assess the assumption of missingness at random and the finding of such assessments. | 1 group | Cover by adding to E+E text of item #45 |
|  | 47 | Cohort study – If applicable, explain how loss to follow-up was addressed. | 1 group | Include after vote as subitem to #42 |
|  | 48 | Case-control study – If applicable, explain how matching of cases and controls was addressed. | 2 groups | Include after vote as subitem to #42 |
|  | 49 | Cross-sectional study – If applicable, describe analytical methods taking account of sampling strategy. | 1 group | Include after vote as subitem to #42 |
|  | 50 | Describe any sensitivity analyses. | 0 groups | Include after vote as subitem to #42 |
|  | 51 | State whether Hardy-Weinberg equilibrium (HWE) was considered and, if so, how. | 2 groups | Combine #51, #52 (vote taken on whether to include or exclude this combined item) |
|  | 52 | Where HWE test is undertaken, quote the p-value threshold applied to determine deviation from HWE. | 2 groups | Combine #51, #52 (vote taken on whether to include or exclude this combined item) |
|  | 53 | Describe any methods used for inferring genotypes or haplotypes. | 2 groups | Include |
|  | 54 | Describe any methods used to assess or address population stratification. | 1 group | Include after vote |
|  | 55 | Describe any methods used to assess and correct for relatedness among subjects. Report results of assessments for relatedness. | 1 group | Include after vote; after the vote the CM participants decided to cover #55 by adding to the E+E text for #34 |
|  | 56 | Describe any assumptions made regarding mode of inheritance. | 1 group | Exclude after vote |
|  | 57 | Provide justification for assumption of mode of inheritance or if no mode is assumed. | 1 group | Exclude |
|  | 58a) | Describe any methods used to address multiple comparisons or to control risk of false positive results due to investigating multiple genetic variants. | 3 groups | Include |
|  | 58b) | Describe any methods used to address multiple comparisons or to control risk of false positive results due to investigating multiple outcomes. | 3 groups | Include |
|  | 58c) | Describe any methods used to address multiple comparisons or to control risk of false positive results due to investigating multiple assumptions regarding mode of inheritance. | 2 groups | Include |
|  | 59 | Describe any methods used to adjust for extent of adherence in the analyses. | 2 groups | Include |
| **Results** | | | | |
| Participants | 60a) | Report the numbers of individuals at each stage of the study – e.g., numbers potentially eligible, examined for eligibility, confirmed eligible, included in the study, completing follow-up, and analysed. | 3 groups | Include |
|  | 60b) | Give reasons for non-participation at each stage. | 0 groups | Cover by adding to E+E text of #60a |
|  | 60c) | Consider use of a flow diagram. | 0 groups | Cover by adding to E+E text of #60a |
|  | 61 | For each genetic variant, report numbers of individuals in whom genotyping was attempted and numbers of individuals in whom genotyping was successful. | 1 group | Exclude after vote |
| SNPs | 62 | Report any SNPs that were excluded from analysis, and provide reasons for these exclusions. | 2 groups | Include after vote |
| Descriptive data | 63 | Give characteristics of study participants (e.g., demographic, clinical, social) and information on potential confounders. | 3 groups | Include |
|  | 64 | Indicate the number of participants with missing data for each variable of interest. | 3 groups | Include by adding to E+E text for #63 |
|  | 65 | For a cohort study, consider giving information listed in (63) and (64) by genotype. | 1 group | Exclude after vote |
|  | 66 | For a case-control study, give the information listed in (63) and (64) for cases and controls separately. | 0 groups | Combine with #63 (add to E+E text) |
|  | 67 | Report reasons for missing genotype data. | 0 groups | Exclude |
|  | 68 | Cohort study – Summarize follow-up time, e.g. average and total amount. | 2 groups | Include after vote |
|  | 69 | Where HWE tests have been undertaken, highlight SNPs that deviate from HWE. | 3 groups | Include |
|  | 70 | Where population stratification is assessed, report the results. | 3 groups | Include |
| Outcome data | 71a) | For a cohort study, report all outcomes (phenotypes) investigated for each genotype category over time. | 3 groups | Include |
|  | 71b) | For a case-control study, report numbers in each genotype category for all outcomes investigated. | 3 groups | Include |
|  | 70c) | For a cross sectional study, report all outcomes (phenotypes) investigated for each genotype category. | 3 groups | Include |
|  | 72 | If a study includes more than one ethnic group, provide the summary data specified in (71) per ethnic group. | 3 groups | Include |
| Main results | 73 | Give unadjusted estimates and, if applicable, confounder-adjusted estimates and their precision (e.g., 95% confidence intervals). Make clear which confounders were adjusted for and why they were included. | 3 groups | Include |
|  | 74 | Report category boundaries when continuous variables were categorised. | 3 groups | Include |
|  | 75 | If relevant, consider translating effect estimates to number needed to test to illustrate potential clinical utility of any significant findings. | 0 groups | Exclude after vote |
|  | 76 | Report results of any adjustments for multiple comparisons. | 3 groups | Cover by adding to E+E text of #73 |
|  | 77 | Report precise p-values for all associations. | 3 groups | Cover by adding to E+E text of #73 |
| Other analyses | 78 | Report other analyses done – e.g., analyses of subgroups and interactions, and sensitivity analyses. | 2 groups | Include |
|  | 79 | If numerous genetic exposures (genetic variants) were examined, summarize results from all analyses undertaken. | 2 groups | Include |
|  | 80 | If detailed results are available elsewhere, state how they can be accessed. | 3 groups | Exclude after vote |
| **Discussion** | | | | |
| Key results | 81 | Summarize key results with reference to study objectives. | 3 groups | Include |
| Limitations | 82 | Discuss limitations of the study, taking into account sources of potential bias or imprecision. Discuss both direction and magnitude of any potential bias. | 3 groups | Include |
|  | 83 | Report on the risk of phenoconversion (genotype-phenotype mismatch) and its magnitude in the study population. | 1 group | Exclude after vote |
| Interpretation | 84 | Give a cautious overall interpretation of results considering objectives, limitations, multiplicity of analyses, results from similar studies, and other relevant evidence. | 3 groups | Include |
|  | 85 | Report genotype frequencies from other studies. | 0 groups | Exclude |
| Generalisability | 86 | Discuss the generalisability (external validity) of the study results. | 3 groups | Include |
|  | 87 | Discuss, if pertinent, the health care relevance of the study results. | 1 group | Exclude after vote |
| **Other information** | | | | |
| Study registration/  protocol | 88 | State whether the protocol for the analysed data is publicly available and if so, how the protocol can be accessed. | 0 groups | Exclude after vote |
|  | 89 | State whether the study has been registered. If the study has been registered, provide details of the registry. | 2 groups | Include after vote |
| Ethical approval | 90a) | Report whether ethical approval was obtained for the collection of genetic data. | 3 groups | Include |
|  | 90b) | If ethical approval was obtained, report the committee that gave ethical approval and a reference ID. | 0 groups | Cover by adding to E+E text of #90a |
| Funding | 91 | Give the source of funding and the role of the funders for the present study and, if applicable, for the original study on which the present article is based. | 3 groups | Include |
| Databases | 92 | State whether databases for the analysed data are or will become publicly available and if so, how they can be accessed. | 1 group | Include after vote |

CM: consensus meeting; E+E: explanation and elaboration document; HWE: Hardy-Weinberg equilibrium; SNP: single nucleotide polymorphism
