# Supplementary material for: STrengthening the Reporting Of Pharmacogenetic Studies: Development of the STROPS guideline
Source: PLoS Med. 2020 Sep 21;17(9):e1003344. doi: 10.1371/journal.pmed.1003344 (PMC7505422; doi:10.1371/journal.pmed.1003344)
Supplement: S1 Document — (DOCX) [file pmed.1003344.s005.docx]

**S1 Document. Delphi participants and consensus meeting attendees**

**Delphi participants:**

Irma Aguilar-Delfín, Dymium BT (Mexico)

José A G Agúndez, Universidad de Extremadura (Spain)

Sophie M Argon, University of Washington (USA)

M J Arranz, Fundació Docència i Recerca MútuaTerrassa (Spain)

Derrick A Bennett, University of Oxford (UK)

Stefan Böhringer, Leiden University Medical Center (The Netherlands)

Lawrence Brody, National Human Genome Research Institute (USA)

Ingolf Cascorbi, University Hospital Schleswig-Holstein (Germany)

Erika Cecchin, National Cancer Institute (Italy)

Mandy Crommentuijn-van Rhenen, Royal Dutch Pharmacists Association (The Netherlands)

Ann K Daly, Newcastle University (UK)

Nur Aizati Athirah Daud, Universiti Sains Malaysia (Malaysia)

Jorge Duconge, University of Puerto Rico (Puerto Rico)

Chiara Fabbri, King’s College London (UK)

Alison Fitches, Springer Nature (New Zealand)

Andrea Gaedigk, Children’s Mercy Kansas City (USA)

Donato Gemmati, University of Ferrara (Italy)

**Claudia Maria Hattinger**, **IRCCS Istituto Ortopedico Rizzoli (Italy)**

Dan Hawcutt, University of Liverpool (UK)

Rachel Huddart, Stanford University (USA)

Evelyne Jacqz-Aigrain, Université de Paris (France)

Slobodan M Janković, University of Kragujevac (Serbia)

Theodora Katsila, National Hellenic Research Foundation (Greece)

Gideon Koren, Ariel University and Shamir Medical Center (Israel)

Beata S Lipska-Ziętkiewicz, Medical University of Gdańsk (Poland)

Thomas Liehr, Jena University Hospital (Germany)

A H Maitland-van der Zee, University of Amsterdam (The Netherlands)

Lisanne E N Manson, Leiden University Medical Center (The Netherlands)

Martin H Maurer, University of Heidelberg (Germany)

Juan Eduardo Megías-Vericat, Hospital Universitari i Politècnic la Fe (Spain)

Taichi Ochi**,** University of Groningen (The Netherlands)

Daniel J O'Connor, Medicines and Healthcare product Regulatory Agency (UK)

Laura B Ramsey, Cincinnati Children’s Hospital Medical Center (USA)

Gad Rennert, Technion - Israel Institute of Technology (Israel)

Francesco Rucci, University of Milan (Italy)

Gaetano Santulli, Albert Einstein College of Medicine (USA)

Aris Saoulidis, University of Cambridge (UK)

Rashmi R Shah, Pharmaceutical Consultant (United Kingdom)

Alessandro Serretti, University of Bologna (Italy)

Andrew Somogyi, University of Adelaide (Australia)

Gere Sunder-Plassmann, Medical University of Vienna (Austria)

Virginia Boso-Ribelles, Hospital General Universitario de Castellón (Spain)

Caroline F Thorn, Stanford University (USA)

Evangelia Eirini Tsermpini, University of Patras (Greece)

Satyanarayana Chakradhara Rao Uppugunduri, University of Geneva (Switzerland)

Michael A van Es, University Medical Center Utrecht (The Netherlands)

Magdalena Zarowiecki, Genomics England (UK)

**Consensus meeting attendees:**

Martin H Maurer, University of Heidelberg (Germany)

Juan Eduardo Megías-Vericat, Hospital Universitari i Politècnic la Fe (Spain)

Aris Saoulidis, University of Cambridge (UK)

Satyanarayana Chakradhara Rao Uppugunduri, University of Geneva (Switzerland)
