# Supplementary material for: STrengthening the Reporting Of Pharmacogenetic Studies: Development of the STROPS guideline
Source: PLoS Med. 2020 Sep 21;17(9):e1003344. doi: 10.1371/journal.pmed.1003344 (PMC7505422; doi:10.1371/journal.pmed.1003344)
Supplement: S2 Document — (DOCX) [file pmed.1003344.s006.docx]

**S2 Document. Investigation of attrition bias in the Delphi survey**

If participants that did not respond to Round 2 of the Delphi survey have different opinions to participants who completed both rounds, then attrition bias has occurred and the results of the Delphi survey may be affected.

We investigated the risk of attrition bias by calculating average Round 1 scores for each participant, and then producing boxplots to show the distributions of these average scores according to whether participants completed Round 2 or not for all Delphi participants (Figure S2A), and for each stakeholder group separately (Figure S2B to Figure S2D). We visually examined these plots and found no evidence to suggest that attrition bias had occurred.


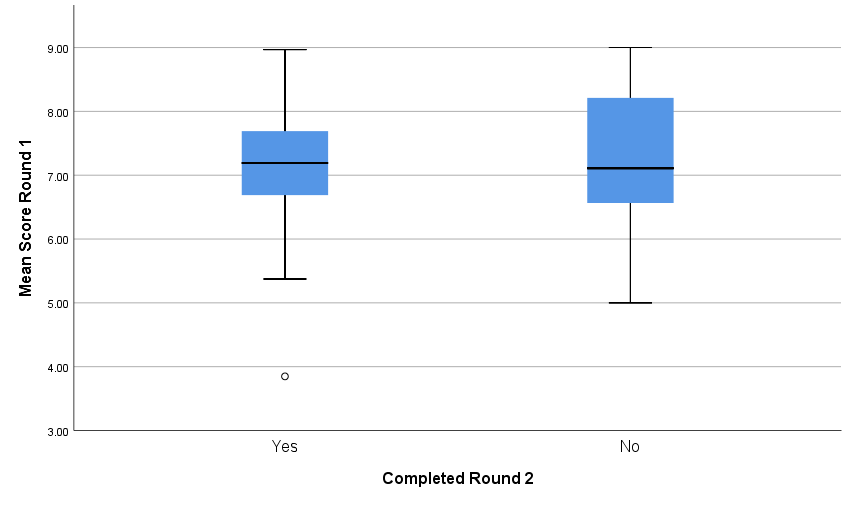


Figure S2A. Investigation of attrition bias: All Delphi participants


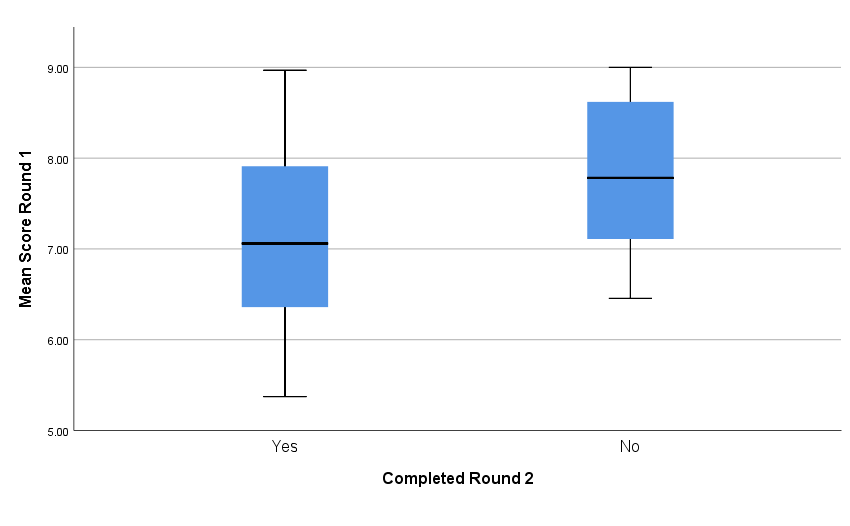


Figure S2B. Investigation of attrition bias: Journal editors


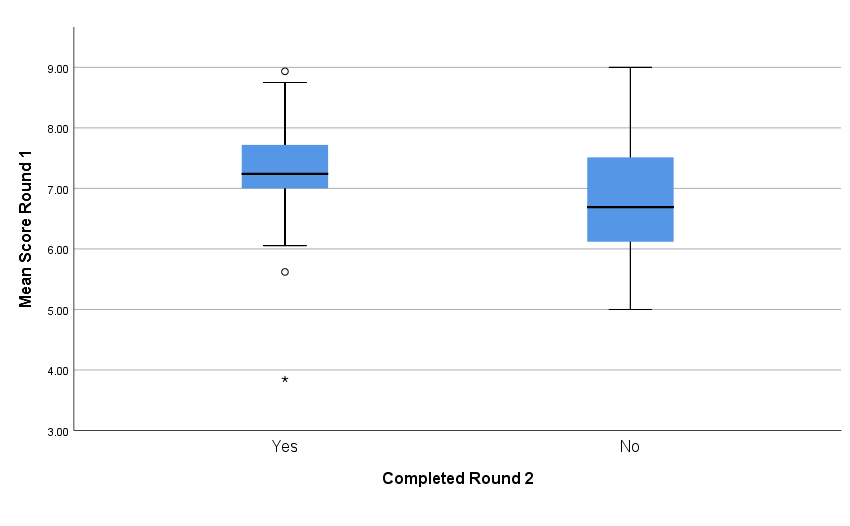


Figure S2C. Investigation of attrition bias: Primary researchers


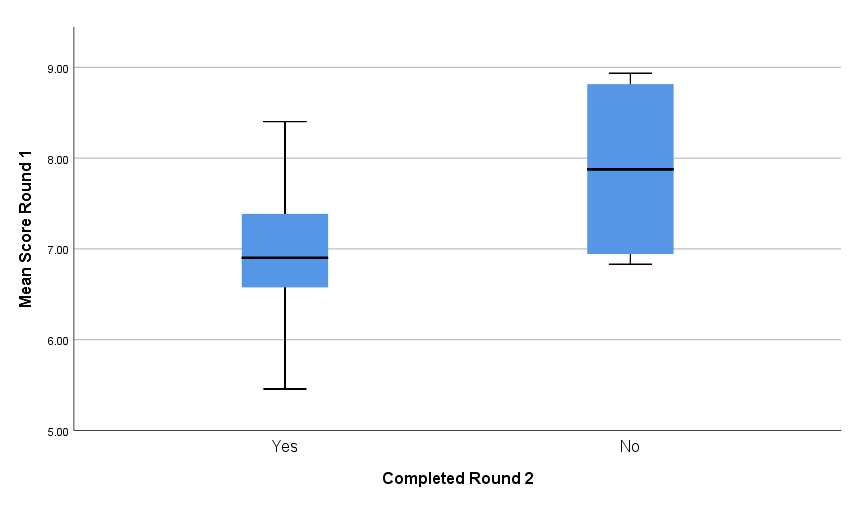


Figure S2D. Investigation of attrition bias: Systematic reviewers
