# Supplementary material for: STrengthening the Reporting Of Pharmacogenetic Studies: Development of the STROPS guideline
Source: PLoS Med. 2020 Sep 21;17(9):e1003344. doi: 10.1371/journal.pmed.1003344 (PMC7505422; doi:10.1371/journal.pmed.1003344)
Supplement: S3 Document — STROPS, STrengthening the Reporting Of Pharmacogenetic Studies. (DOCX) [file pmed.1003344.s007.docx]

# S3 Document. STROPS guideline: Explanation and elaboration document

# Abstract

### 1. Provide in the abstract an informative and balanced summary of what was done and what was found.

#### Explanation

Study authors should provide the key information that enables readers to understand the research question, study design, methods, results and conclusions of the study. This item is from the STROBE statement [1] (Item 1b); further guidance and rationale for this item is detailed in the STROBE explanation and elaboration paper [2]. An example abstract is provided below; however it is important to note that journals may specify their own set of guidelines, which authors ought to follow in terms of abstract structure and content.

#### Example

“INTRODUCTION: Approximately 30% of patients with epilepsy are resistant to treatment with anti-epileptic drugs (AEDs). The ABC drug transporter proteins are hypothesized to mediate drug resistance in epilepsy. More recently, a non-ABC putative transporter, RLIP76, has also been proposed to be involved in the mechanism of pharmacoresistance. One previous association study of six polymorphisms in *RLIP76* failed to find any association with drug resistance in a retrospective cohort of epilepsy patients. We aimed to look for an association with outcomes reflecting drug response in a larger prospective cohort, with gene-wide coverage.

PATIENTS AND METHODS: We investigated the role of common polymorphisms in *RLIP76* in epilepsy pharmacoresistance by genotyping 23 common *RLIP76* polymorphisms in a prospective cohort of 503 epilepsy patients, from the standard and new anti-epileptic drugs (SANAD) prospective study of new and old AEDs. A total of 13 of these were tested for association with four outcomes reflecting response to drugs: time to first seizure, time to 12-month remission, time to withdrawal due to inadequate seizure control, and time to withdrawal due to unacceptable adverse drug events.

RESULTS: No significant associations, allowing for multiple testing, were found in the whole cohort. There was also no effect in a subgroup of patients on carbamazepine, which is thought to be a RLIP76 substrate, although two polymorphisms were associated with time to first seizure (p=0.007).

DISCUSSION: We failed to demonstrate any association between *RLIP76* polymorphisms and four different measures of drug response in the larger cohort, but a subgroup analysis of patients receiving carbamazepine suggested an association that should be investigated further.

CONCLUSIONS: Our data suggest that common variants in *RLIP76* are unlikely to contribute to epilepsy drug response” [3].

# Introduction

## Background/rationale

### 2. Explain the scientific background and rationale for the investigation being reported.

#### Explanation

Study authors should provide the rationale for conducting the pharmacogenetic study in the context of existing research in this health area, i.e. what is known on a topic and what gaps in current knowledge are addressed by the study. This item is from the STROBE statement [1] (Item 2); further guidance and rationale for this item is detailed in the STROBE explanation and elaboration paper [2].

#### Example

“Inhaled corticosteroids (ICS) are recommended for adults and children with asthma and for chronic obstructive pulmonary disease (COPD). Although ICS are generally well tolerated and have fewer systemic adverse effects than do oral corticosteroids, some patients can still develop systemic adverse effects. Adrenal suppression is a clinically important adverse effect, particularly in children with asthma, in whom the diagnosis of adrenal suppression can be challenging because presentation can range from asymptomatic biochemical changes to nonspecific lethargy to florid adrenal crisis and death (…).

Interindividual variation in susceptibility to adrenal suppression is striking (…). The reasons for the interindividual variability in both adults and children remain unclear, because clinical factors only account for a small proportion of the variance.

Previous pharmacogenomic studies in patients with asthma using corticosteroids have focused on efficacy. As far as we know, no studies examining the pharmacogenomics of corticosteroid-induced adrenal suppression have been reported. The aim of the Pharmacogenetics of Adrenal Suppression with Inhaled Steroids (PASS) study was to undertake a pharmacogenomics assessment of factors predisposing to corticosteroid-induced adrenal suppression among children with asthma using ICS as part of their treatment” [4].

### 3. Provide reasons for choosing the genes and SNPs genotyped.

#### Explanation

It is important that researchers conducting a candidate gene study choose the genes and SNPs to be investigated in a systematic way, using prior knowledge to guide their decisions. Study authors should explain how the investigated genes and SNPs were chosen, with reference to relevant functional/animal studies, previous association studies, and any procedures used, such as examining linkage disequilibrium patterns (the "tagging SNP" approach), or assessing the likelihood of each individual SNP affecting the gene function with priority given to those with the most likely functional effect. Clear rationale instils confidence in the reader that all analyses performed have been reported, rather than only statistically significant or interesting results (i.e. selective reporting of results).

It is important to note that for next generation sequencing (NGS), criteria may be applied to prioritise genetic variants for association analyses. In this case, filtering options ought to be specified.

The origin of this item is Jorgensen and Williamson’s quality assessment checklist for pharmacogenetic studies [5].

#### Example

“More recently, a novel putative mechanism of epilepsy multidrug resistance has been described. RLIP76, also known as RALBP1, is alleged to be a non-ABC multispecific transporter, which transports a variety of drugs, with a similar substrate specificity to PGP [P-glycoprotein]. A study by Awasthi et al. showed that RLIP76 was upregulated in brain tissue from drug-resistant epilepsy patients, and colocalized with PGP in endothelial cells. RLIP76 was shown to transport both phenytoin and carbamazepine in an isolated artificial liposome system and in crude membrane vesicles. Furthermore, RLIP76 blockade with anti-RLIP76 antibodies altered phenytoin and carbamazepine transport to a much greater extent than did PGP blockade, indicating the potential importance of RLIP76 in AED [anti-epileptic drug] transport.

(...) All SNPs in the *RLIP76* gene in HapMap Phase 1, and exonic and untranslated region SNPs from dbSNP, were selected for genotyping. Primer design was successful for 23 polymorphisms (Figure 1), which were genotyped on the Sequenom MALDI-TOF mass extension platform at the Sanger Institute (...)” [3].

## Objectives

### 4. State specific objectives, including any pre-specified hypotheses.

#### Explanation

Study authors should provide the objectives for the study, specifying the relevant population, genetic variants, drugs and outcomes. This item is from the STROBE statement [1] (Item 3); further guidance and rationale for this item is detailed in the STROBE explanation and elaboration paper [2].

#### Example

“The primary objective of this study was to determine, in a large patient cohort, whether c.516G>T and c.983T>C polymorphisms are predisposing factors for nevirapine hypersensitivity in a Malawian HIV-infected adult population. Secondly, we aimed to investigate whether carriage of *HLA-C*04:01* in combination with variants of *CYP2B6* increases the risk for nevirapine hypersensitivity” [6].

### 5. State if the study is the first report of a pharmacogenetic association, a replication effort, or both.

#### Explanation

This item is from the STREGA statement [7] (Item 3), although we modified the item to be more applicable to pharmacogenetic studies. In the STREGA statement, the item reads: “State if the study is the first report of a genetic association (…)”. Further guidance and rationale for this item is detailed in the STREGA statement publication.

#### Example

“As far as we know, no studies examining the pharmacogenomics of corticosteroid-induced adrenal suppression have been reported. The aim of the Pharmacogenetics of Adrenal Suppression with Inhaled Steroids (PASS) study was to undertake a pharmacogenomic assessment of factors predisposing to corticosteroid-induced adrenal suppression among children with asthma using ICS [inhaled corticosteroids] as part of their treatment. Validation was undertaken in both a paediatric asthma cohort (enrolled to the PASS study) and an adult COPD [chronic obstructive pulmonary disease] cohort (enrolled to the Pharmacogenomics of Adrenal Suppression in COPD [PASIC] study)” [4].

# Methods

## Study design

### 6. Present key elements of study design early in the paper.

#### Explanation

Study authors should state the study design used, and present key features of the study design so that readers can understand the basics of the study, e.g. for a cohort study: describe the group of people that comprised the cohort and the time period for which they were followed; for a case-control design: describe the cases and controls and their source population; for a post-hoc pharmacogenetic analysis of a randomised controlled trial (RCT): state how the subjects included in the analysis were chosen, including which arm of the RCT they were from. This item is from the STROBE statement [1] (Item 4); further guidance and rationale for this item is detailed in the STROBE explanation and elaboration paper [2].

#### Example

“Blood samples, demographic and clinical data from patients initiating warfarin for venous thromboembolism or atrial fibrillation between November 2004 and March 2006 were collected, as described previously (…).

In this prospective cohort study (...), all patients received usual clinical care with doses being determined either by the anticoagulant clinic or attending physician. There were four fixed study visits for each patient, the first at the time of initiation of warfarin (index visit), then at 1 week, 8 weeks and 26 weeks of warfarin therapy” [8].

## Setting

### 7. Describe the setting, locations and relevant dates, including periods of recruitment, follow-up, and data collection.

#### Explanation

Study authors should provide sufficient information to enable readers to assess the context and generalisability of a study's results. It is advisable to specify dates rather than length of time periods, i.e. the dates that recruitment began and ended, the dates that follow-up began and ended, and the date of data collection. This item is from the STROBE statement [1] (Item 5), although we modified the item to be applicable to pharmacogenetic studies. We removed the reference to periods of “exposure”, as pharmacogenetic studies do not investigate the effects of “exposures”, (other than genotype, which is fixed over time). Further guidance and rationale for this item is detailed in the STROBE explanation and elaboration paper [2].

#### Example

“This prospective study was conducted at National Taiwan University Hospital, a tertiary-care center in Taiwan (...).

From March 2007 to February 2010, adult patients (>16 years) with culture-confirmed pulmonary TB [tuberculosis] were enrolled as the derivation cohort. Mycobacterial culture and drug susceptibility testing were performed as previously described. Subjects were excluded if they were pregnant, had a life expectancy 6 months, had abnormal baseline liver function test (LFT), or had Mycobacterium tuberculosis (MTB) isolates resistant to INH [isoniazid], RMP [rifampin], or both. From March 2010 to February 2013, TB patients fulfilling these criteria were enrolled as the validation cohort (...).

The LFT was checked at 2, 4, 6, 8, 12, and 16 weeks after the start of anti-TB treatment or whenever symptoms of hepatitis developed during the initial 6 months of anti-TB treatment” [9].

## Participants

### 8. Give the eligibility criteria, and the sources and methods of selection of participants. For a cohort study, describe methods of follow-up. For a case-control study, state whether true controls or population controls were used. Give the rationale for the choice of cases and controls.

#### Explanation

Study authors should provide sufficiently detailed descriptions of the study participants to help readers understand the applicability of the results. In a case-control study, true controls are controls who have been exposed to the relevant treatment but have not developed the outcome of interest. If historical controls have been used, specify the setting in which this data was collected. Population controls are individuals who have already been genotyped that can be assumed to be controls, although we cannot ascertain whether they would have developed the outcome of interest if they had been exposed to the relevant treatment.

This item is derived from the STROBE statement [1] (item 6a); we added the specification that study authors should state whether true controls or population controls were used, as the use of population controls is a common feature in pharmacogenetic studies. Further guidance and rationale for this item is detailed in the STROBE explanation and elaboration paper [2].

#### Example: Cohort study

“Patients initiated onto warfarin irrespective of indication were recruited from the Royal Liverpool and Broadgreen University Hospitals Trust and University Hospital Aintree between November 2004 and March 2006. The only exclusion criterion was inability or refusal to give informed consent (…).

There were four fixed study visits for each patient, the first at the time of initiation of warfarin (index visit), then at 1 week, 8 weeks and 26 weeks of warfarin therapy (…). At the index visit, patient demographics were recorded and baseline INR [international normalised ratio], clotting factor activity and protein levels were measured (Table 1). At the remaining follow-up visits INR was again measured, and dose changes since the previous visit were recorded. In addition to the four fixed study visits, patients also attended anticoagulant clinic according to their clinical needs. This meant that, at the end of follow-up, data on warfarin dose changes and INR levels were available longitudinally for each patient, which together provided a complete picture of treatment progress from warfarin initiation onwards. For patients who missed one or more fixed follow-up visits, INR measurements and dose changes missing as a consequence were obtained from clinical records” [10].

#### Example: Case-control study with population controls

“From a cohort of ~600,000 patients receiving statins identified in the CPRD [the UK Clinical Practice Research Datalink] (www.cprd.com), a case-control design was used to identify suitable patients for the study, as previously described. Participation was restricted to white people ≥18 years of age and with the first ever statin prescription at least 1 year after the start of CPRD data collection.

All cases conformed to internationally agreed standards for statin-induced myopathy and rhabdomyolysis. Cases were categorized into two groups: (i) myopathy: patients who discontinued their implicated statin with a rise in CK [creatine kinase] > 4 × ULN [upper limit of normal]; and (ii) severe myopathy: individuals with a history of rhabdomyolysis or CK  > 10 × ULN after statin exposure (…).

Population control genotype data for the initial discovery case-control GWAS [genome-wide association study] was obtained from the Wellcome Trust Case-Control Consortium 2 (WTCCC2) cohort of 2,501 individuals from the UK Blood Service” [11].

#### Example: Case-control study with true controls

“The study cohort consisted of older adults with polypharmacy and history of cardiovascular disease to ensure homogeneity of the study sample. The cases were presented by eligible individuals with the history of FH [frequent hospitalisations]. The controls included eligible patients with infrequent hospitalizations (IHs) randomly drawn from the study cohort, based on case–control matching criteria. On the basis of a previous work, cases (FH) were defined as individuals who were hospitalized at least 3 times during the past 2 years” [12].

This article provides a reference to previous work conducted to justify their choice of cases and controls.

### 9. Report the drug and regime participants were exposed to, and the length of exposure.

#### Explanation

The purpose of pharmacogenetic studies is to explore how genetic variants influence individuals’ responses to drugs. Study authors should provide details of drug and regime, length of exposure, and route of administration. This could be fixed across all participants or variable. If variable, authors should provide this information in the text or in a table of patient characteristics. We recommend that authors use generic drug terms from a standardised database e.g. DrugBank where possible. This item was conceived by members of the Steering Committee.

#### Example

“All patients received oral INH (300 mg), rifampicin (600 mg), pyrazinamide (20 mg/kg body weight), and ethambutol (800 mg) daily for the first 2 months. Pyrazinamide was then discontinued, while INH, rifampicin and ethambutol were continued for another 4 months” [13].

### 10. For a matched case-control study, give matching criteria and the number of controls per case.

#### Explanation

Study authors should provide details of variables that were used to match individuals to make case and control groups more comparable. This item is from the STROBE statement [1] (item 6b), although we modified the item to be more applicable to pharmacogenetic studies. We removed the reference to matched cohort studies; following searches of the literature we found very few pharmacogenetic studies that used a matched cohort design, and this item would therefore be irrelevant to the vast majority of guideline users. Further guidance and rationale for this item is detailed in the STROBE explanation and elaboration paper [2].

#### Example

“For each case, two controls were recruited, within the same cohort and on the same ATD [anti-tuberculosis drug] (INH, RIF, PZA) [isoniazid, rifampicin, pyrazinamide] but with serum ALT [alanine aminotransferase] levels <3 times ULN [upper limit of normal], serum bilirubin <1 mg/dL and no history of severe nausea, vomiting within the first 3 months of initiation of therapy. Controls were matched with cases on the basis of age, sex, disease severity and drug dosage” [14].

### 11. Give information on the criteria and methods for selection of subsets of participants from a larger study, when relevant.

#### Explanation

If one or more sub-samples from a larger study are used for the investigation of a pharmacogenetic association, authors should provide details of: inclusion and exclusion criteria, sources and methods of selection for these sub-samples, and state whether these methods were pre-specified or post-hoc. This item is from the STREGA statement [7] (Item 6a); rationale for this item is detailed in the STREGA statement publication.

#### Example

“In a post hoc analysis of a 4.3 year placebo-controlled randomized trial with 390 patients with Type 2 diabetes [T2D] already on insulin, we analyzed the influence of polymorphisms in genes coding for ATM and the transporters OCT1 and MATE1 (…). The HOME trial is a 4.3 year randomized placebo controlled trial that included 390 Caucasian patients aged 30–80 years with T2D treated with insulin. Patient selection, study design, data collection and power analysis have been described previously. Figure 1 shows the trial design and the recruitment plus retention of patients for the current study” [15].

Figure 1 of this publication provides information on the criteria for including patients in the post-hoc pharmacogenetic analysis of the RCT.

### 12. If other publications report results for the same patient cohort, or a subset of the patient cohort, provide information on this patient cohort overlap and references to the relevant publications.

#### Explanation

In pharmacogenetic research, it is common for multiple articles to report data for the same patient cohort (or for overlapping patient cohorts); different articles may report on different outcomes and genetic variants. To aid interpretation, it is useful to highlight overlapping or identical cohorts across articles. This item was conceived by members of the Steering Committee.

#### Example

“Given the association between *CYP4F2* and warfarin dose requirements, and the emerging evidence that this P450 isoform is involved in the metabolism of vitamin K1, we have undertaken a comprehensive analysis of *CYP4F2*SNPs and haplotypes in a prospectively recruited cohort of patients from two UK clinics (...).

Patients (n = 311) were recruited prospectively as they were initiated onto warfarin at two hospitals in Liverpool, the Royal Liverpool and Broadgreen University Hospitals Trust and University Hospital Aintree (...). Analyses of association between the *CYP2C9* and *VKORC1*, and 27 other genes, and the response to warfarin are reported in the accompanying manuscript” [16].

The article provides a reference to the accompanying manuscript in the text.

### 13. Report disease/clinical indication of patients using a standardised ontology when possible.

#### Explanation

To aid interpretation, authors should use controlled vocabularies such as MeSH and SNOMED to describe the disease/clinical indication of patients. This item was suggested by Delphi participants at Round 1 of the survey.

#### Example

“We included children aged 5–18 years with asthma using ICS [inhaled corticosteroids] as part of their treatment” [4].

## Variables

### 14. Clearly define all outcomes, potential confounders, and effect modifiers. Give diagnostic criteria, if applicable.

#### Explanation

Study authors should define all outcomes, and all variables considered for and included in the analysis. If outcomes are categorised into “primary” and “secondary” outcomes, this should be specified. Authors should use controlled vocabularies such as MeSH and SNOMED to describe phenotypes.

This item is from the STROBE statement [1] (item 7), although we modified the item to be applicable to pharmacogenetic studies. We removed reference to “predictors” from the item as this is not relevant to pharmacogenetic studies, and to “exposures”, as the exposure in a pharmacogenetic study is the genetic variant; definition of genetic variants is covered in the STROPS guideline item 16. Further guidance and rationale for this item is detailed in the STROBE explanation and elaboration paper [2].

#### Example

“For this GWAS [genome-wide association study], we used the following outcome measures: (1) Mean weekly dose (MWD): mean dose received weekly during a minimum follow-up time of 14 days post-loading; the loading period, that is, the first 3 days of treatment, was not included in the calculations. (2) Stable mean weekly dose (SMWD): mean weekly dose for at least three consecutive visits where INRs [international normalised ratios] were within the targeted range, spanning a minimum of 14 days and with at least 7 days separating the first and middle INR measurements, and the middle and last one. (3) INR >4.0 in the first week on warfarin (...).

Non-genetic variables used for testing univariately for association with each outcome were age, height, weight, BMI [body mass index], gender, loading dose, total follow-up time, dosing method (manual or computerised), mean target INR, blood count (haemoglobin, platelets, white cells, neutrophils, basophils, lymphocytes, monocytes, eosinophils), potassium, bicarbonate, chloride, urea, creatinine, triglycerides, albumin, total protein, bilirubin, ALT [alanine transaminase], alkaline phosphate, gamma GT, fibrinogen, coagulation factors II, V, VII, IX and X, Proteins C and S, current smoking status, number of cigarette smoked per day, ex-smoker status, alcohol consumption, interacting co-medication (binary), non-interacting co-medication (binary), sum of effect of interacting co-medications. The coagulation factors were measured as described by Jorgensen et al. For each variable, either a linear (quantitative outcomes) or logistic (binary outcome) regression was used to test for association with outcome in R, and variables found to be significant univariately (P ≤0.05) were included as covariates in the linear or logistic regressions used to test for association between each SNP and outcome in turn” [8].

### 15. Provide justification for choice of outcomes.

#### Explanation

Study authors should explain why the outcomes are important, e.g. clinical importance, importance to patients, inclusion in previously developed core outcome sets, identification of a significant association in previous studies, etc. A core outcome set is an agreed standardised set of outcomes that should be measured and reported, as a minimum, in all clinical studies in specific areas of health or health care. The COMET database [17] lists references to planned, ongoing and completed core outcome set work for a wide range of health topics. Providing a clear justification for the choice of outcomes provides reassurance to the reader that selective reporting of results has not occurred, i.e. results have not been omitted from the report due to the significance or perceived importance of the estimate of association. The origin of this item is Jorgensen and Williamson’s quality assessment checklist for pharmacogenetic studies [5].

#### Example

“Guidelines for TB management recommend a combination regimen including isoniazid (INH), rifampin, ethambutol, and pyrazinamide as the first-line treatment. This regimen often causes adverse drug reactions, such as hepatitis, cutaneous reactions, gastrointestinal upset, and drug fever. Although mild reactions can be tolerated or managed with symptomatic therapy, serious cases require discontinuation of medication and prolongation of the treatment period (…).

Previous studies in an Indian and a Taiwanese population reported that homozygous null mutations in *GSTM1* increased the risk of ATD-induced hepatitis. In contrast to these findings, a subsequent study in Spain failed to validate this association, reporting instead that null mutations in *GSTT1* were associated with ATD-induced hepatotoxicity. Thus, the association between null mutations of *GSTT1* or *GSTM1* and ATD-induced hepatitis remains unclear and needs to be replicated in other ethnic groups.

ATD-induced cutaneous reactions, such as rashes, can be serious adverse reactions and their incidence is higher than that of hepatitis and gastrointestinal reactions. Despite the clinical significance of ATD-induced cutaneous reactions, not much is known about genetic predisposition to these reactions. It is suggested that, like drug-induced liver injury, hypersensitivity reactions to reactive metabolites underlie the mechanisms of drug eruption. Langerhans cells and epidermal keratinocytes have been suggested to play pivotal roles in the development of drug-induced hypersensitivity reactions in the skin. Drug metabolites transferred into or bioactivated in the skin can induce an immune response after haptenization. Detoxification by GST enzymes may be involved in the development of cutaneous reactions to ATD. Therefore, we hypothesized that null mutations of *GSTT1* and *GSTM1* genes are associated with ATD-induced cutaneous reactions. To our knowledge, there is no published report on the association between genetic polymorphisms in GST enzymes and ATD-induced skin reactions. In this study, we examined whether null mutations in

*GSTT1* and *GSTM1* were associated with the development of ATD-induced hepatitis and adverse cutaneous reactions in a Korean population” [18].

### 16. Clearly define genetic exposures (genetic variants) using a widely-used nomenclature system.

#### Explanation

Study authors should state all genetic variants that were screened (including SNPs, indels, copy number variations, and structural variations) and specify the tissue source of DNA, as recommended by McDonagh et al [19]. This information could be provided in supplementary materials if necessary. It is also useful for study authors to provide references to any databases and resources used for the selection of variants.

The Human Gene Nomenclature Committee have published guidelines for human gene nomenclature [20, 21]. The Human Genome Variation Society has detailed information about how to describe variant locations (<http://varnomen.hgvs.org/recommendations/general/>), as recommended by McDonagh et al. [19] and Thorn et al. [22].

This item is from the STREGA statement [7] (Item 7b); further guidance is detailed in the STREGA statement publication.

#### Example

“Venous blood sample collection followed informed consent from the parents or guardians of the participants and DNA was extracted using a standard salting-out method (…).

SNPs were submitted to Illumina (CA, USA) Technical Support for evaluation using the Assay Design Tool. SNPs were scored (varying from 0–1) by the Assay Design Tool based on compatibility to successful GoldenGate genotyping. A total of 14 SNPs with a score above 0.6 were selected for genotyping (summarized in [Table 1](https://www.futuremedicine.com/doi/full/10.2217/pgs.11.106#T1))” [23].

Table 1 of this article gives gene names, rs numbers, and HGVS names for all the genotyped SNPs.

### 17. Report the rs number of each genotyped SNP.

#### Explanation

An "rs" number (reference SNP ID number) is an identification tag assigned by NCBI (National Center for Biotechnology Information) to a group (or cluster) of SNPs that map to an identical location. The rs ID number, or rs tag, is assigned after submission of an SNP to dbSNP [24]. Reporting an rs number for each genotyped SNP allows the reader to identify the same SNP across multiple articles. Guidelines are available for variations not listed in dbSNP [25, 26]. In particular, submission ID or the position of the SNP and reference sequence ID for the chromosome should be specified. This item was conceived by members of the Steering Committee.

#### Example

“A total of 448 individuals were genotyped for the rs4149056 SNP in *SLCO1B1* and rs4693075 in *COQ2*” [27].

### 18. Clearly state how haplotypes or star alleles were defined.

#### Explanation

Even when referring to well-defined star alleles or haplotypes, authors should provide full details in the current publication using standard nomenclature. This allows the reader to identify the same haplotypes/star alleles across multiple articles, and can provide reassurance that haplotypes/star alleles have been defined according to widely accepted nomenclature. Standard nomenclature for some haplotypes are available on the PharmVar database [28, 29]. This item was suggested by Delphi participants at Round 1 of the survey.

#### Example

“*SULT4A1-1* status was assigned to all CATIE subjects (…) using rs2285162 (A) and rs2285167 (G) as the haplotype tagging SNPs” [30].

### 19. If referring to the minor, major, wild-type, mutant, reference, risk or effect allele of a variant, state which allele this is and for which given population/cohort.

#### Explanation

The allele and population should be clearly stated if using any of these terms. For many genetic variants, the minor/mutant/risk/effect (less frequent) allele in one population may be the major/wild-type/reference (more frequent) allele in a different population. This item was suggested by Delphi participants at Round 1 of the survey.

#### Example

“The risk allele (T) frequency was highest in East Asians…” [31].

## Data sources/measurement

### 20. For each variable of interest, give sources of data and details of methods of assessment (measurement). Describe comparability of assessment methods if there is more than one group.

#### Explanation

Authors should provide information on how all confounders and outcomes were measured. It is also important to report any differences in how data were collected in different patient groups (e.g. cases and controls). This item is from the STROBE statement [1] (item 8); further guidance and rationale for this item is provided in the STROBE explanation and elaboration paper [2].

#### Example

“Warfarin dose and INR [international normalised ratio] values for each POC [point of care] testing event is routinely collected for all patients using warfarin at our institution, and this data were extracted onto our study database. From this information, each patient’s full dose and INR history could be determined.

Data were also collected on indication for treatment, target INR, age, gender, height, weight, BMI [body mass index], haemorrhagic complications, serial serum albumin concentration(s) (as many children were hypoalbuminaemic at onset of therapy), and height and weight measurements. Clinical data were collected from hospital notes and the cardiac liaison team database” [32].

### 21. Describe laboratory methods, including source and storage of DNA, genotyping methods and platforms (including the allele calling algorithm used, and its version), error rates and call rates. State the laboratory/centre where genotyping was done. Describe comparability of laboratory methods if there is more than one group. Specify whether genotypes were assigned using all of the data from the study simultaneously or in smaller batches.

#### Explanation

Authors should provide sufficient details to enable the reader to assess the potential extent of genotyping errors (a source of information bias). It is important to report any differences in laboratory methods in different patient groups (e.g. cases and controls). Furthermore, if the study uses a case-control design, authors should report whether cases and controls were put into mixed batches for genotyping purposes (to ensure genotyping quality is comparable across groups), rather than analysed in separate batches. It is important to note that for next generation sequencing (NGS), library preparation, instrument, coverage level, pipeline and tools for variant calling should also be specified. This item is from the STREGA statement [7] (Item 8b); further guidance and rationale for this item is detailed in the STREGA statement publication.

#### Examples

“Whole blood on FTA® cards was obtained from 37 deceased Finnish individuals (...). All subjects and toxicology data were collected according to the ethical handling of human subject practices of the University of Helsinki. Anonymized DNA samples were transferred to University of North Texas Health Science Center (UNTHSC) and handled according to the UNTHSC Institutional Review Board Protocol Number 2016-051 (...).

DNA was extracted from FTA cards using the QIAGEN® QIAamp® DNA Blood Mini Kit and total human DNA was quantitated using the ThermoFisher Scientific Quantifiler™ Trio DNA Quantification Kit according to the respective manufacturers’ recommendations (...).

Genotyping was performed using the Illumina Infinium® LCG Assay and Infinium® Omni2.5Exome-8 v1.3 BeadChip according to the manufacturer’s recommended protocol. Template DNA input ranged from 200 to 1 ng genomic DNA. Image acquisition was performed on the Illumina HiScan™ System using the iScan Control Software (...).

“BeadChip images were analyzed in GenomeStudio® Genotyping Module v2.0.2, following the manufacturer’s recommended quality control procedures including a Genotype Call (GenCall) Score cutoff of 0.15.”

This article provides detailed information on call rates and evaluation of genotyping errors in supplementary materials to the publication.

“Samples pertaining to matched cases and controls were analyzed in the same batch, and laboratory personnel were unable to distinguish between cases and controls” [33].

### 22. Describe genotype quality control methods and findings.

#### Explanation

It is important to report any quality control methods and findings so that the reader is able to assess how reliable the genotyping results, and consequently, the findings of a study are. Genotype quality control methods include using negative controls, and re-genotyping all or a random sample of patients. The origin of this item is Jorgensen and Williamson’s quality assessment checklist for pharmacogenetic studies [5].

#### Example

“Samples were genotyped at Affymetrix’s service laboratory on the Genome-Wide Human SNP Array 6.0. Genotype data quality control was via the standard protocol that was established for the WTCCC2 studies (supplementary methods). Specifically, concordance check was performed on 116 SNPs by 1779 individuals overlapped between this GWA [genome-wide association] data and the WTCCC1 T2D [type 2 diabetes] case control study. Based on the concordance rate of 99.73%, individuals with more than 10% discordance were removed from the current study. After such stringent QC [quality control], the clean data set included 705125 autosomal SNPs on 3736 samples, of whom 1024 have definable metformin response” [34].

### 23. For quantitative outcome variables, specify if any investigation of potential bias resulting from pharmacotherapy was undertaken. If relevant, describe the nature and magnitude of the potential bias, and explain what approach was used to deal with this.

#### Explanation

Bias from pharmacotherapy may occur when quantitative outcome variables are affected by treatment with drugs other than the study drug (e.g. outcome variables include biochemical markers of hepatotoxicity, and several patients are taking concomitant hepatotoxic medications). This item is from the STREGA statement [7] (Item 9b); further guidance and rationale for this item is detailed in the STREGA statement publication.

#### Example

“…use of drugs with anti- or pro-emetic effects may also influence the occurrence and intensity of nausea and vomiting in cancer patients, but have not been thoroughly investigated yet (…).

All regressions were stratified by country and use of antiemetics was included as a binary stratification variable (antiemetics used/not used) for each country” [35].

### 24. Report how adherence to treatment was assessed, and report the results of the assessment.

#### Explanation

In general, treatment adherence is not an issue of great concern for non-pharmacogenetic studies of drug efficacy. This is because the aim of these trials is to estimate how effective the drug will be when used in a real-world setting; in reality, patients are likely to occasionally be non-adherent with the prescribed regimen, so the trial results will be reflective of clinical practice. However, the aim of pharmacogenetic studies is to identify associations between genetic variants and drug response outcomes. Treatment adherence is therefore an important issue in pharmacogenetic studies; taking too much or too little of the prescribed drug undoubtedly may impact drug response outcomes. Providing information on treatment adherence assessment methods and results allows the reader to consider whether adherence may have had an impact on outcomes. The origin of this item is Jorgensen and Williamson’s quality assessment checklist for pharmacogenetic studies [5].

#### Example

“Treatment compliance was assessed by comparing the number of administered treatment doses to the number of treatment doses scheduled each month. There were no consistently missing doses corresponding to more than 10 days of monthly scheduled doses of medication for all the patients” [13].

## Study size

### 25. Explain how the study size was arrived at, or provide details of the a priori power to detect effect sizes of varying degrees.

#### Explanation

Study authors ought to report the calculation performed to obtain the study sample size, providing references to any specific methodology. Or, if sample size was predetermined (for example, if the study reports secondary analyses of a published dataset), provide details of a priori power calculations for a range of plausible effect sizes. This item is derived from the STROBE statement [1] (item 10); we added the detail that providing power calculations for a range of plausible effect sizes is sufficient to address this item, as it is not uncommon for sample size to be predetermined in pharmacogenetic studies. For example, in a post-hoc pharmacogenetic analysis of a RCT, sample size would be limited by the number of participants included in the RCT who it would be possible to obtain genotype information for. Further guidance and rationale for this item is detailed in the STROBE explanation and elaboration paper [2].

#### Examples

“As we are investigating a wide range of gene-outcome associations it is difficult to provide precise power calculations in advance. However, we can calculate power for some simplistic analyses. A key variable is the minor allele frequency (MAF) among controls. For rarer variants to be clinically important, their effect size (odds ratio (OR)) must be large. We therefore specify two benchmarks for the power analyses: we seek to have good power for (a) OR=3 and a rare variant (MAF=5%); (b) OR=2 and a common variant (MAF=20%). Assuming a type I error rate of 5% and 80% power, for scenario a) we would require 115 cases and 230 controls and for scenario b) we would require 123 cases and 246 controls. If we increased our sample size to 250 cases and 500 controls we would have 80% power to observe and odds ratio of 2.2 for a MAF of 5%, and an odds ratio as small as 1.6 for a MAF of 20%. Please note that these effect sizes are for a single causal variant. We expect to realise much larger overall effect sizes via combinations of causal variants” [36].

“The target sample size for the primary analysis cohort was 500. To arrive at this estimate we considered two possible scenarios: (a) an odds ratio (OR) of 3 for association between a rare variant (minor allele frequency=5%) and the primary outcome; (b) an OR of 2 for association between a common variant (minor allele frequency=20%) and the primary outcome. A liberal type 1 error rate of 5% was assumed on the basis that validation cohorts would also be analysed to help eliminate false positives arising from the initial analyses. Assuming first of all prevalence of impaired adrenal response in children with asthma using inhaled steroid to be 17% (based on a the [*sic*] lower end of the range of previously published rates of adrenal suppression) the power for scenario a) was calculated as 77% and the power for scenario b) was calculated as 75%. If the prevalence was 40% (the upper end of the range of published estimates), the power increased to 91% for both scenarios. A prevalence of 20% would ensure power of at least 80% in both scenarios.” [4] (Supplementary Appendix).

## Quantitative variables

### 26. Explain how quantitative variables (confounders and effect modifiers) were handled in the analyses. If applicable, describe which groupings were chosen, and why.

#### Explanation

If continuous variables have been grouped into categories to create a new categorical variable, it is important to explain why and how quantitative data were grouped. This item is from STROBE [1] (item 11), although we added detail to make it clear that the item relates to confounders and effect modifiers, rather than quantitative outcome variables. Further guidance and rationale for this item is detailed in the STROBE explanation and elaboration paper [2].

#### Example

“The potential genetic risk scores ranged from 0 to 3 (…). The risk scores were dichotomized as low risk (0–1 points) and high risk (2–3 points) because only a small number of subjects had a score of 0 and 3, and they responded similarly to those with a score of 1 and 2, respectively” [37].

## Statistical methods

### 27. Address the following:

1. Describe methods used to control for confounding.
2. Describe any methods used to examine subgroups and interactions.
3. Explain how missing data were addressed.
4. Cohort study – If applicable, explain how loss to follow-up was addressed.
5. Case-control study – If applicable, explain how matching of cases and controls was addressed.
6. Describe any sensitivity analyses.

#### Explanation

All statistical methods should be reported clearly, including details of which analyses were pre-specified and which were exploratory based on data inspection. Details of any software used should also be provided. The STROBE explanation and elaboration document [2] advises that sufficient detail should be provided that a statistically competent reader with access to the data set would be able to verify reported results based on the reported methods. In particular:

1. If adjustments were made for confounding factors, authors ought to provide details of procedures of variable selection and model comparison.
2. Study authors should explain any methods used to examine whether associations differed across subgroups, or to examine interactions. An "interaction" occurs when one factor modifies the effect of another, and is also sometimes referred to as ‘effect modification'.
3. Authors should confirm whether analyses were restricted to individuals with complete data on the required variables, or whether any imputation of missing data was performed. Data are said to be ‘missing at random’ if the fact that they are missing is unrelated to actual values of the missing data. Data that are missing at random may not be important. Analyses based on the available data will tend to be unbiased, but will also be based on a smaller sample size than the original data set. If authors assessed the assumption of missingness at random, the methods and findings of these assessments should be provided.
4. For a cohort study, authors ought to report how many individuals were lost to follow-up, and whether these individuals were excluded or whether censoring strategies were used.
5. For a matched case-control study, authors should describe in detail what statistical methods were used to account for the matching of cases and controls.
6. Provide details of any sensitivity analyses i.e. analyses performed to investigate whether the results of the main analysis are consistent with those obtained with alternative analysis approaches.

This item is from STROBE [1] (items 12a, 12b, 12c, 12d and 12e). Further guidance and rationale for this item is provided in the STROBE explanation and elaboration paper [2].

#### Examples

1. “First, a univariate multinomial logistic regression model was fitted for each non-genetic factor in turn, to identify which non-genetic factors to adjust for in the SNP association analyses. Next, multivariable multinomial logistic regression models were fitted for each SNP in turn. For each SNP, two models were fitted. The first model included covariates to represent all non-genetic factors with p<0.25 univariately. Stepwise variable selection was applied to this baseline model to remove any covariates no longer significant in the multivariable model. The final model following variable selection was called the ‘baseline model’. The second model (‘the genetic model’) was the same as the baseline model but also included a covariate to represent the SNP. The likelihood ratio test was applied to compare the two models and thus assess for statistical significance of the SNP” [38].
2. “In the multivariate logistic regression analysis, an interaction variable between sex and *PXR* genotypes and haplotypes was also included” [9].
3. “For some of the non-genetic variables there was a considerable amount of missing data (see Table 1). In order to minimise the impact of this, multiple imputation using chained equations, specifically the predictive mean matching method, was used to impute data for height, weight and albumin at the start of warfarin treatment (all variables had <30% missing observations). Multiple imputation was not used for these variables at the time stable dose was achieved, as the amount of missingness was deemed too high (>40%). Instead, these variables were excluded from the list of potential covariates” [32].
4. “For analyzing EFS [event-free survival] and PFS [progression-free survival], patients who stopped imatinib or switched treatment during follow-up were censored at the time of stopping or switching. However, these patients could be informative, as their reason for stopping/switching is related to the events investigated. Therefore, 2 sensitivity analyses were undertaken to determine whether censoring was informative. The first assumed that these patients were at high risk of an event and that all censored observations were therefore EFS/PFS events occurring immediately after censoring. The second assumed that these patients were at low risk of an event and assumed that all EFS/PFS events happened after the latest follow-up; their censoring time was changed to the time of last follow-up” [39].
5. “The Wilcoxon signed-rank test was used while comparing the means of continuous variables from matched samples. The exact McNemar’s test was used on paired nominal data” [12].
6. “Sensitivity analyses were also undertaken, in which each analysis was repeated, but cases without evidence of H. pylori infection were excluded” [40].

### 28. State whether Hardy-Weinberg equilibrium was considered and, if so, how.

#### Explanation

Authors should describe any statistical tests or measures of departure from Hardy-Weinberg equilibrium (HWE), and any methods used to allow for deviations from HWE. Where HWE tests have been undertaken, it is important to state the p-value threshold applied to determine deviation from HWE. This item is from the STREGA statement [7] (item 12f); further guidance and rationale for this item is provided in the STREGA statement publication.

#### Example

“Prior to analysis, each SNP was tested for Hardy–Weinberg Equilibrium (HWE) using Fisher’s exact test. Those with a p-value of less than 0.001 were excluded from further analyses” [3].

### 29. Describe any methods used for inferring genotypes or haplotypes.

#### Explanation

Study authors ought to provide details of any statistical methods or software used to infer genotype phase and haplotypes. This item is from the STREGA statement [7] (item 12g); further guidance and rationale for this item is provided in the STREGA statement publication.

#### Example

“We used multiple imputation methods to infer remaining missing genotype values on the basis of the correlational structure of the observed genotypes (…).

2677G→T/A and 1236C→T were successfully genotyped in 96% and 94% of individuals, respectively (…). Based on these genotypes, haplotypes were inferred with PHASE” [41].

This article refers to the software program, PHASE [42], which can be used to estimate haplotypes.

### 30. Describe any methods used to assess or address population stratification.

#### Explanation

Authors should explicitly document any methods used to assess the presence of population stratification or adjust for population stratification in the analyses. If no methods were used, this should be made clear in the study report. This item is from the STREGA statement [7] (item 12g); further guidance and rationale for this item is provided in the STREGA statement publication.

#### Example

“In the primary analysis cohort, to test for association, regression models assuming an additive genetic model were fitted in SNPtest using each SNP as a covariate in an independent model. To adjust for population substructure we included up to five principal components as covariates in this genome-wide analysis, subject to the principal components being significantly associated with the outcome univariately (p<0.05). If no principal components were significantly associated with the outcome, the first two principal components were included as covariates in genome-wide analysis.” [4] (Supplementary Appendix).

This article refers to the software program, SNPtest [43], which can be used to analyse single SNP associations in genome-wide studies.

### 31. Describe any methods used to assess and correct for relatedness among subjects. Report results of assessments for relatedness.

#### Explanation

It is not uncommon in pharmacogenetic studies for some participants to be related. Authors should report any methods used to assess relatedness, results of these assessments, and any methods used to correct for relatedness. This item is from the STREGA statement [7] (item 12j), although we added the specification that results of assessments for relatedness ought to be provided. Further guidance and rationale for this item is provided in the STREGA statement publication.

#### Example

“Patients within the primary analysis cohort were excluded from association analyses if any of the following criteria were met: (...) c) the pairwise identity by descent (IBD) statistic of relatedness was >0.1875 (patient with lowest call rate of the pair excluded)” [4] (supplementary materials).

“92 (18%) children failed genotype quality control, of whom (...) nine did not meet identity-by descent criteria” [4].

### 32. Describe any methods used to address multiple comparisons or to control risk of false positive results due to investigating:

1. multiple genetic variants
2. multiple outcomes
3. multiple assumptions regarding mode of inheritance.

#### Explanation

Pharmacogenetic studies that perform a large number of statistical tests are at risk of type 1 errors. This item is derived from STREGA [7] (item 12i), but we modified the item to specify different possible sources of multiplicity in pharmacogenetic studies, i.e. multiple outcomes, genetic variants, and assumptions regarding mode of inheritance. Mode of inheritance refers to the way in which a genetic trait is passed from one generation to the next, e.g. dominant, recessive, co-dominant inheritance. Authors may undertake multiple analyses, each making a different assumption about the underlying mode of inheritance, or making no assumption about the mode of inheritance. Study authors should provide sufficient detail to enable the reader to assess the likelihood of false positive results (type 1 errors) being reported. Rationale for this item is provided in the STREGA statement.

#### Example

“To account for multiple testing, the false discovery rate (FDR) was calculated in addition to the P value for each test of association. In calculating the FDR, all tests for association undertaken on the dataset, including those referred to in an accompanying manuscript, were taken into account” [16].

### 33. Describe any methods used to adjust for extent of adherence in the analyses.

#### Explanation

As discussed under item 24, treatment adherence is an important issue in pharmacogenetic studies. It is important to take treatment adherence into consideration in statistical analyses for pharmacogenetic studies, as recommended by Jorgensen and Williamson in their quality assessment checklist for pharmacogenetic studies [5] (the origin of this reporting item).

Generally, adjusting for a non-confounding covariate (such as treatment adherence) can explain variability in the outcome, consequently reducing noise and increasing power to detect pharmacogenetic associations [44]. However, it is important to note that when the drug response outcome is binary, and individuals are recruited according to case or control status, adjusting for the covariate can actually reduce power [45]. Nevertheless, methods have been developed to overcome this issue [46-48], which account for non-confounding covariates while increasing power to detect genetic associations in case-control studies. It is therefore advisable to account for treatment adherence when investigating pharmacogenetic associations, even in the analyses of case-control studies, providing careful consideration is given to the choice of analysis method.

#### Example

“Repeating analyses of genetic association but adjusting for adherence: For any of the outcomes found significantly associated with nonadherence in the univariate analyses, the analyses of association with each of the 196 SNPs as previously reported in Jorgensen et al. were repeated, but this time after adjusting for adherence. To do this, for each SNP in turn, two proportional hazard regression models were compared using the likelihood ratio test. The first model included a covariate representing adherence status; the second was the same but also included a covariate to represent the SNP.

Finally, for the outcome of stable dose, an alternative approach was also adopted where the outcome itself was reduced by the estimated proportion of doses missed and the analyses for association with clinical and genetic factors as reported in Jorgensen et al. repeated with this revised outcome. For testing for association with genetic factors, two tests of association were undertaken for each SNP. The first made no assumptions regarding the underlying mode of inheritance and used ANOVA [analysis of variance] to test for association and the second assumed an additive mode of inheritance and used univariate linear regression. For testing for association with clinical factors, Student’s t-test was used for binary variables, ANOVA for categorical variables and linear regression for continuous variables” [49].

# Results

## Participants

### 34. Report the numbers of individuals at each stage of the study – e.g., numbers potentially eligible, examined for eligibility, confirmed eligible, included in the study, completing follow-up, and analysed.

#### Explanation

Authors ought to report the numbers of individuals considered at each stage of recruiting study participants, alongside reasons for non-participation at each stage. This allows the reader to judge whether the study population was representative of the target population, and whether bias was possibly introduced. A flow diagram can be an efficient and transparent way to convey this information. This item is from STROBE [1] (item 13a). Further guidance and rationale for this item is provided in the STROBE explanation and elaboration paper [2].

#### Example

“From October 2007 to June 2008, a total of 4488 newly diagnosed patients with sputum smear positive pulmonary TB [tuberculosis] were recruited from four provinces (Zhejiang, Guangxi, Chongqing, Jilin) in China (…). A total of 4304 patients finished the follow-up (…).

Patients with any of the following were excluded from the present study: (i) abnormal serum ALT [alanine aminotransferase], AST [aspartate aminotransferase] or total bilirubin levels before anti-TB treatment; (ii) carriers of the hepatitis B or C virus; (iii) alcoholic liver disease or habitual alcohol drinking; (iv) the concomitant use of hepatotoxic drugs; and (v) a history of chronic liver disease or systemic diseases that may cause liver dysfunction. Among the remained patients, those fulfilled the criteria of ATDH [anti-TB drug-induced hepatotoxicity] were assigned into the case group. Incidence density sampling method was adopted to select controls from patients free of ATDH up to the date when the paired cases were diagnosed with ATDH. For each ATDH case, four controls were randomly selected and matched with age (within 5 years old), sex, treatment history, disease severity, drug dosage and place of sample collection. Finally, 89 patients with ATDH and 356 matched controls were included in the study” [50].

## SNPs

### 35. Report any SNPs that were excluded from analysis, and provide reasons for these exclusions.

#### Explanation

Authors should provide explicit statements of why variants considered important initially were excluded from analyses, for example, due to excessive missing data [5]. This provides assurance to the reader that no additional investigations have been undertaken, and that all analyses have been fully reported. The origin of this item is Jorgensen and Williamson’s quality assessment checklist for pharmacogenetic studies [5].

#### Example

“Of the 23 SNPs genotyped across *RLIP76* (Table 1), six SNPs failed successful genotyping, three SNPs were found to have a MAF of less than 1% (indeed one was monomorphic), and one SNP was found to deviate from HWE” [3].

Table 1 of this publication provides the rs number of each genotyped SNP, whether the SNP was included in analyses, and reasons for exclusion where applicable.

## Descriptive data

### 36. Give characteristics of study participants (e.g., demographic, clinical, social) and information on potential confounders.

#### Explanation

Study authors ought to report participant characteristics (e.g. age, sex, ethnicity, special characteristics such as pregnancy and concomitant medications) with appropriate summary measures. For example, for continuous data, mean and standard deviation, or median and range; for dichotomous data, numbers and proportions. For a case-control study, this information ought to be provided for cases and controls separately. It is also important to indicate the number of participants with missing data for each variable.

This item is from the STROBE statement [1] (Item 14a), although we removed the reference to “exposures”, as the exposure in a pharmacogenetic study is the genetic variant. We would expect information on patient genotypes to be reported with the outcome data; it is not mandatory to provide this information as part of the baseline characteristics of the study population. Further guidance and rationale for this item is detailed in the STROBE explanation and elaboration paper [2].

#### Example

“The baseline demographic factors including underlying comorbidities are summarized in Table 1. The majority of patients (n=309), 60% of whom were inpatients, had a target INR [international normalised ratio] range of 2–3, with the remaining two patients having a target range of 3–4. The majority of the patients were White with atrial fibrillation being the most common indication for warfarin therapy. There was significant variation in the loading doses prescribed. Sixty-three percent were given 10 mg on the first 2 days, 17% were given 10 mg on day 1 followed by 5 mg on day 2, 7% were given 3 mg on both days whereas 5% were given 5 mg on both days” [10].

Table 3 of this publication summarises key baseline characteristics for patients included in this study; footnotes are used to indicate the amount of missing data for each variable.

### 37. Cohort study – Summarize follow-up time, e.g. average and/or total amount.

#### Explanation

Average follow-up can be summarised using the mean and/or median follow-up time. Total amount of follow-up may be reported using total person-years of follow-up, or some indication of the completeness of follow-up [51]. This item is derived from the STROBE statement [1] (Item 14c); we modified the item slightly to indicate that average and/or total follow-up time would satisfy this criteria. Further guidance and rationale for this item is detailed in the STROBE explanation and elaboration paper [2].

#### Example

“In our study, patients were entered into the study at the time they started antiepileptic drug treatment, and were followed-up prospectively to determine their response to medication, including both seizure control and adverse events (...).

Follow-up ranged from 84 days to 2296 days (median 934, mean 1041)” [41].

### 38. Where HWE tests have been undertaken, highlight SNPs that deviate from HWE.

#### Explanation

Although deviations from HWE may be due to disturbing factors which the researcher has no control over, it is also possible that deviation may be caused by genotyping errors [52], by the existence of population stratification or by biased selection of controls [53]. In the quality assessment checklist for pharmacogenetic studies (the origin of this item) [5], Jorgensen and Williamson recommend that study authors highlight any SNPs that were found to deviate from HWE.

#### Example

“The rs3813867 SNP was not in Hardy-Weinberg equilibrium and was therefore excluded from (...) analysis” [54].

### 39. Where population stratification is assessed, report the results.

#### Explanation

Study authors ought to report the results of any tests performed to detect the presence of population stratification. If tests determine that population stratification is present, this indicates that the study is at risk of confounding. Therefore, any associations detected may be spurious findings. It is essential that readers are informed of the results of any tests for population stratification to enable appropriate interpretation of study findings. The origin of this item is Jorgensen and Williamson’s quality assessment checklist for pharmacogenetic studies [5].

#### Example

“The data collected showed that the African, European, and Amerindian ancestry mean ratios were not significantly different between the two groups examined (p > 0.05). Figure 1 shows the individual parental ethnic contribution of the case group (patients with hepatotoxicity) and control group (patients without hepatotoxicity) estimated through 48 AIMs [ancestry informative markers]” [55].

## Outcome data

### 40a) For a cohort study, report all outcomes (phenotypes) investigated for each genotype category over time.

### 40b) For a case-control study, report numbers in each genotype category for all outcomes investigated.

### 40c) For a cross sectional study, report all outcomes (phenotypes) investigated for each genotype category.

#### Explanation

All outcome data should be reported clearly, including the amount of missing genotype/outcome data. This item is derived from STREGA [7] (item 15); we modified the item to specify that all investigated outcomes ought to be reported, to emphasise the importance of not selectively reporting results. Specific guidance on how to report outcome data for each type of study design are as follows:

1. Cohort study: For outcomes that relate to the occurrence of some event, report the number of events that occurred. If the risk of an event occurring changes over follow-up time, present the numbers and rates of events in appropriate intervals of follow-up or as a Kaplan-Meier life table or plot. For other outcomes, present appropriate summary measures (e.g. means and standard deviations) over time.
2. Case-control study: Report numbers of cases and controls in each genotype category.
3. Cross-sectional study: For outcomes that relate to the occurrence of some event, report the number of events that occurred. For other outcomes, present appropriate summary measures (e.g. means and standard deviations).

#### Example: Cohort study

In a cohort study conducted by Ramsey et al. [30], the authors report change in PANSS-T (Positive and Negative Syndrome Scale total score) (summarised by means and standard deviations in Table 2), response rate and completer status (summarised by percentage of responders and completers in Table 3) and weight gain (summarised by mean and standard errors in Figure 1) for each haplotype group.

#### Example: Case-control study

“Associations between -308G/A and ATD [anti-tuberculosis drug]-induced hepatitis: The genotype frequencies in the case and control groups (...) are presented in Table 2.”

Table 2 of this publication reports the number and percentage of cases and controls in each genotype group (AA, AG, or GG) [56].

#### Example: Cross-sectional

In a cross-sectional study conducted by Ebid et al. [57], the numbers of responders and non-responders to metformin and glimepiride combination therapy in each genotype group is presented in Table 2 of the publication for the two investigated SNPs (*SLC22A1* rs622342 and *ABCC8* rs757110).

### 41. If a study includes more than one ethnic group, provide the summary data specified in (40) per ethnic group.

#### Explanation

Due to the possibility of confounding by population stratification, it is advisable to present results stratified by ethnicity. Furthermore, a significant association between a SNP and a treatment response outcome does not necessarily indicate a causal relationship; it is possible that the association only exists due to the SNP of interest being in strong linkage disequilibrium with the causal SNP. Patterns of linkage disequilibrium vary from one population to the next [58], and therefore differences in estimates of association may be observed between different populations.. This provides further rationale for stratifying results by ethnicity. This item was conceived by members of the Steering Committee.

#### Example

In a case-control study conducted by Ng et al. [59], the number of cases and controls in each *NAT2* acetylator group (assigned by genotype) is presented in Table 3 of the publication, for the overall study population, for patients from Europe, and for patients from the Indian subcontinent.

## Main results

### 42. Give unadjusted estimates and, if applicable, confounder-adjusted estimates and their precision (e.g., 95% confidence intervals). Make clear which confounders were adjusted for and why they were included.

#### Explanation

Providing both unadjusted measures of association and measures of association adjusted for potential confounders enables readers to compare both measures and assess how the measure of association is impacted by adjusting for confounders. Study authors ought to list all potential confounder variables considered, and the criteria/rationale for excluding or including variables in statistical models. It is also useful for authors to:

- Clearly state which allele/genotype of the variant is associated with the phenotype and the direction of the association, when associations are reported
- Report results of any adjustments for multiple comparisons, for example, Bonferroni adjusted p-values, or false discovery rates.
- Report precise p-values for all associations, as opposed to only indicating whether an association was found to be statistically significant or not. For example, stating p<0.05 or p>0.05, or indicating statistical significance (or a lack of) by using asterisks (*) is not appropriate.

This item is from the STROBE statement [1] (Item 16a), further guidance and rationale for this item is detailed in the STROBE explanation and elaboration paper [2].

#### Example

In a retrospective cohort study conducted by Higashi et al. [60], the authors present unadjusted and unadjusted estimates in Table 5, and specify that warfarin daily dose is the only covariate included in the adjusted model. The authors explain that covariates were included in the model if the hazard ratio changed by more than 5% upon inclusion of the covariate in the model.

### 43. Report category boundaries when continuous variables were categorised.

#### Explanation

If continuous outcomes were categorised, study authors ought to report the range of values covered by each category. This item is from the STROBE statement [1] (Item 16b), further guidance and rationale for this item is detailed in the STROBE explanation and elaboration paper [2].

#### Example

“(…) patients were divided according to the HbA1C level obtained at the patient clinic appointment and classified into responders or non-responders. Responders were defined as patients who received metformin and glimepiride combination therapy for at least 6 months, and their HbA1C was less than 7%. Non-responders were considered to be on combination therapy for at least 6 months, and their HbA1C was equal or higher than 7%” [57].

## Other analyses

### 44. Report other analyses done – e.g., analyses of subgroups and interactions, and sensitivity analyses.

#### Explanation

It may be impractical to present detailed findings for all analyses performed; in this case, authors should present detailed results for important results only. Less important results can be summarised briefly in the text and detailed in full in supplementary materials. This item is from the STROBE statement [1] (Item 17), further guidance and rationale for this item is detailed in the STROBE explanation and elaboration paper [2].

#### Example

“In a sensitivity analysis in which only those patients without any evidence of H. pylori infection (n=376) were analysed as cases, there was no significant interaction seen with *CYP2C19*17* (P=0.068) (see Supplementary Data online)” [40].

### 45. If numerous genetic exposures (genetic variants) were examined, summarize results from all analyses undertaken.

#### Explanation

This item encourages authors to report results for all genetic variants that were investigated in the study, rather than selectively reporting only "interesting" or statistically significant results. Full results can be provided in supplementary materials if necessary. This item is from the STREGA statement [7] (item 17b), further guidance and rationale for this item is detailed in the STREGA statement publication.

#### Example

“In the logistic regression analyses using an additive model, only one SNP, *CYP2C19*17*, was significantly associated with the presence of PUD [peptic ulcer disease] (odds ratio 1.47 (95% confidence interval (CI) 1.12 to 1.92); P=0.005, Table 3)” [40].

Table 3 of this publication provides the results of logistic regression analyses for all analysed SNPs.

### 46. If detailed results are available elsewhere, i.e. in supplementary materials, state how they can be accessed.

#### Explanation

Study authors ought to report what results are available, and provide sufficient details that a reader would easily be able to locate these resources. This item is derived from the STREGA statement [7] (item 17c), although we modified the item to indicate that we are referring to supplementary materials to the study publication. Further guidance and rationale for this item is detailed in the STREGA statement publication.

#### Example

“Supplementary material is linked to the online version of the paper at <http://www.nature.com/cpt>” [40].

# Discussion

## Key results

### 47. Summarize key results with reference to study objectives.

#### Explanation

A short summary of the main findings of the study helps the reader to assess whether the author's interpretation and suggested implications are supported by the findings. This item is from the STROBE statement [1] (item 18); further guidance and rationale for this item is detailed in the STROBE explanation and elaboration paper [2].

#### Example

“In this large study in Malawian and Ugandan adults treated with nevirapine, we have been able to show an association between nevirapine-induced SJS/TEN [Stevens-Johnson syndrome/toxic epidermal necrolysis] and the c.983T>C polymorphism. The carriage frequency in nevirapine control individuals was 16% compared with 32% in those with nevirapine-induced SJS/TEN (P=0.0005, FDR [false discovery rate]=0.015) (Table 4). This association was not observed with any other nevirapine-induced hypersensitivity phenotype” [6].

## Limitations

### 48. Discuss limitations of the study, taking into account sources of potential bias or imprecision. Discuss both direction and magnitude of any potential bias.

#### Explanation

Discussing the limitations of the study helps the reader to interpret the validity and health care relevance of the study findings. Limitations might relate to, for example, characteristics of included patients, methods of outcome measurement, multiplicity of analyses, missing data, etc. This item is from the STROBE statement [1] (item 19); further guidance and rationale for this item is detailed in the STROBE explanation and elaboration paper [2].

#### Example

“A limitation of our study is the size of the replication cohort, which was smaller than the discovery cohort and therefore lacked the statistical power to truly replicate the association observed between c.983T>C and nevirapine-induced SJS/TEN [Stevens-Johnson syndrome/toxic epidermal necrolysis]. However, nevirapine hypersensitivity is a rare phenotype and it was difficult to identify a larger number of patients. The replication cohort also consisted of both Malawian and Ugandan patients, which may introduce some population stratification. However, our data show that the frequency of c.983T>C polymorphism in the overall combined Malawian discovery and replication patients (0.18) was comparable to that observed in the Ugandan patients (0.15)” [6].

## Interpretation

### 49. Give a cautious overall interpretation of results considering objectives, limitations, multiplicity of analyses, results from similar studies, and other relevant evidence.

#### Explanation

When interpreting results, authors should consider potential sources of bias, residual confounding (due to unmeasured variables or imprecise measurement of confounders), the results of relevant sensitivity analyses and subgroup analyses. Authors should discuss the real range of uncertainty with regards to reported results, which is greater than the statistical uncertainty demonstrated by confidence intervals. This item is from the STROBE statement [1] (item 20); further guidance and rationale for this item is detailed in the STROBE explanation and elaboration paper [2].

#### Example

“Utilizing a variety of outcomes reflecting drug response, we have not demonstrated a significant association with any of 13 common SNPs in the *RLIP76* gene in the whole cohort, either when investigated univariately or by way of multiple regression, taking the genetic region spanned by the SNPs as a whole. Three SNPs demonstrated low p-values when undertaking the univariate analyses, but these, with p-values of 0.02-0.04, would not survive correction for multiple testing to account for the number of analyses being undertaken. Furthermore, each of these SNPs was found to be ''nominally significant'' for only one of any of the four outcomes investigated. Following backward variable selection, one SNP was found to be nominally associated with time to first seizure (SNP rs167897) and another was found to be nominally associated with time to 12-month remission (SNP rs12457094), but once again, the resulting p-values would not survive correction for multiple testing.

In the subgroup analysis, including only those patients on carbamazepine, the only AED [anti-epileptic drug] used in the SANAD cohort that may be a substrate for RLIP76, univariate testing of the 13 SNPs demonstrated four SNPs with p-values less than 0.05, but once again, no SNP demonstrated a significant association with more than one of the four outcomes, and furthermore, once again these p-values would not survive correction for multiple testing. When investigating the genetic region as a whole, a nominally significant association was found with the outcome of time to first seizure (p=0.05), and the resulting models following backward variable selection gave p-values of less than 0.05 for all four outcomes (minimum: p=0.007). One SNP in particular, rs329017, was retained in the final model for three of the four outcomes. While once again these p-values would not survive correction for multiple testing, this is the strongest evidence for any influence of *RLIP76* genetic variation on drug response; the smaller size of the carbamazepine-treated subgroup may have limited the power to detect a stronger association.

The lack of definitively positive findings suggests that *RLIP76* genotypes probably have no influence on drug response in epilepsy patients as a whole, which is consistent with those reported recently. Subgroup analysis in patients on carbamazepine alone, however, does raise candidate polymorphisms for further analysis. Even under the assumption that none of these results are significant, this does not exclude a genetic influence on drug response mediated by RLIP76 since common polymorphisms analyzed may not represent all common variants throughout the gene. Alternatively, genetic contributions may arise from rare variants, in which case our study may have lacked power to detect an association. In addition, genetic factors influencing RLIP76 expression or function may be remote from the *RLIP76* gene itself” [3].

The article provides a reference to a previous study which also investigated the association between *RLIP76* genotypes and drug response in epilepsy patients, and makes comparisons between the findings of the two studies.

## Generalisability

### 50. Discuss the generalisability (external validity) of the study results.

#### Explanation

Study authors ought to consider the extent to which the results of the study can be applied to other circumstances, i.e. different populations/settings/countries. This item is from the STROBE statement [1] (item 21); further guidance and rationale for this item is detailed in the STROBE explanation and elaboration paper [2].

#### Example

“Our data show that the frequency of c.983T>C polymorphism in the overall combined Malawian discovery and replication patients (0.18) was comparable to that observed in the Ugandan patients (0.15), and to that reported in a Mozambican population (0.14). Although genetic differences do exist between these African cohorts, it would appear from our study that *CYP2B6* c.983T>C is likely to be generalizable across other sub-Saharan-African populations” [6].

# Other information

## Study registration

### 51. State whether the study has been registered. If the study has been registered, provide details of the registry.

#### Explanation

Studies can be registered on many different official platforms; the most widely used platform is ClinicalTrials.gov. This platform provides information on how registering studies fulfils a number of purposes and benefits many different groups of people. This item was conceived by members of the Steering Committee.

#### Example

“Clinical Trials. gov Identifier: NCT 00824772” [61].

## Ethical approval

### 52. Report whether ethical approval was obtained for the collection of genetic data.

#### Explanation

If ethical approval was obtained, authors should also report the committee that gave ethical approval and a reference ID. This item was conceived by members of the Steering Committee.

#### Example

“Ethical approval for this study was granted by North West 3 Research Ethics Committee (10/H1002/57)” [38].

## Funding

### 53. Give the source of funding and the role of the funders for the present study and, if applicable, for the original study on which the present article is based.

#### Explanation

The role of the funders relates to which part of the study the funders took direct responsibility for, e.g., study design, data collection, analysis, drafting of manuscript, decision to publish. This item is from the STROBE statement [1] (item 22); further guidance and rationale for this item is detailed in the STROBE explanation and elaboration paper [2].

#### Example

“Role of the funding source: The sponsors of the study had no role in study design, data collection, data analysis, data interpretation, or writing of the report. The corresponding author had full access to all the data in the study and had final responsibility for the decision to submit for publication (…).

Acknowledgments: We thank the NHS Research and Development Health Technology Assessment Programme as sponsors of the SANAD study, all clinicians involved in the collection of blood samples, all involved at the Sanger Institute in DNA preparation, genotyping and bioinformatics, and the Wellcome Trust for their support. GDL was supported in part by a Neurology Entry/Exit Scholarship from the Guarantors of Brain. This study and DNA collection was funded by the Wellcome Trust” [41].

## Databases

### 54. State whether databases for the analysed data are or will become publicly available and if so, how they can be accessed.

#### Explanation

If databases are available, study authors should provide sufficient details that a reader would easily be able to locate these resources. This item is from the GRIPS statement [62] (item 24); further guidance and rationale for this item is detailed in the GRIPS explanation and elaboration paper [63].

#### Example

“The complete data set of genotypes and clinical variables, as well as the full genotype quality-control data, is available to registered PharmGKB users at [www.pharmgkb.org](http://www.pharmgkb.org) (full data set accession number, PA162355460)” [64].

# References

1. Von Elm E, Altman DG, Egger M, Pocock SJ, Gøtzsche PC, Vandenbroucke JP. The Strengthening the Reporting of Observational Studies in Epidemiology (STROBE) statement: guidelines for reporting observational studies. Ann Intern Med. 2007;147(8): 573-577.

2. Vandenbroucke JP, von Elm E, Altman DG, Gøtzsche PC, Mulrow CD, Pocock SJ, et al. Strengthening the Reporting of Observational Studies in Epidemiology (STROBE): explanation and elaboration. Int J Surg. 2014;12(12): 1500-1524.

3. Leschziner GD, Jorgensen AL, Andrew T, Williamson PR, Marson AG, Coffey AJ, et al. The association between polymorphisms in RLIP76 and drug response in epilepsy. Pharmacogemonics. 2007;8(12): 1715-1722.

4. Hawcutt DB, Francis B, Carr DF, Jorgensen AL, Yin P, Wallin N, et al. Susceptibility to corticosteroid-induced adrenal suppression: a genome-wide association study. Lancet Respir Med. 2018;6(6): 442-450.

5. Jorgensen AL, Williamson PR. Methodological quality of pharmacogenetic studies: issues of concern. Stat Med. 2008;27(30): 6547-6569.

6. Carr DF, Chaponda M, Cornejo Castro EM, Jorgensen AL, Khoo S, Van Oosterhout JJ, et al. CYP2B6 c. 983T> C polymorphism is associated with nevirapine hypersensitivity in Malawian and Ugandan HIV populations. J Antimicrob Chemother. 2014;69(12): 3329-3334.

7. Little J, Higgins JP, Ioannidis JP, Moher D, Gagnon F, Von Elm E, et al. STrengthening the REporting of Genetic Association studies (STREGA): an extension of the STROBE statement. Hum Genet. 2009;125(2): 131-151.

8. Bourgeois S, Jorgensen A, Zhang EJ, Hanson A, Gillman MS, Bumpstead S, et al. A multi-factorial analysis of response to warfarin in a UK prospective cohort. Genome Med. 2016;8(1): 2.

9. Wang JY, Tsai CH, Lee YL, Lee LN, Hsu CL, Chang HC, et al. Gender-dimorphic impact of PXR genotype and haplotype on hepatotoxicity during antituberculosis treatment. Medicine (Baltimore). 2015;94(24).

10. Jorgensen AL, Al-Zubiedi S, Zhang JE, Keniry A, Hanson A, Hughes DA, et al. Genetic and environmental factors determining clinical outcomes and cost of warfarin therapy: a prospective study. Pharmacogenet Genomics. 2009;19(10): 800-812.

11. Carr DF, Francis B, Jorgensen AL, Zhang E, Chinoy H, Heckbert SR, et al. Genomewide association study of statin‐induced myopathy in patients recruited using the UK Clinical Practice Research Datalink. Clin Pharmacol Ther. 2019;106(6): 1353-1361.

12. Finkelstein J, Friedman C, Hripcsak G, Cabrera M. Pharmacogenetic polymorphism as an independent risk factor for frequent hospitalizations in older adults with polypharmacy: a pilot study. Pharmgenomics Pers Med. 2016;9: 107.

13. Cho H-J, Koh W-J, Ryu Y-J, Ki C-S, Nam M-H, Kim J-W, et al. Genetic polymorphisms of NAT2 and CYP2E1 associated with antituberculosis drug-induced hepatotoxicity in Korean patients with pulmonary tuberculosis. Tuberculosis (Edinb). 2007;87(6): 551-556.

14. Chatterjee S, Lyle N, Mandal A, Kundu S. GSTT1 and GSTM1 gene deletions are not associated with hepatotoxicity caused by antitubercular drugs. J Clin Pharm Ther. 2010;35(4): 465-470.

15. Out M, Becker ML, van Schaik RH, Lehert P, Stehouwer CD, Kooy A. A gene variant near ATM affects the response to metformin and metformin plasma levels: a post hoc analysis of an RCT. Pharmacogenomics. 2018;19(8): 715-726.

16. Zhang JE, Jorgensen AL, Alfirevic A, Williamson PR, Toh CH, Park BK, et al. Effects of CYP4F2 genetic polymorphisms and haplotypes on clinical outcomes in patients initiated on warfarin therapy. Pharmacogenet Genomics. 2009;19(10): 781-789.

17. COMET. Core Outcome Measures in Effectiveness Trials [cited 2018 15 November]. Available from: <http://www.comet-initiative.org/>.

18. Kim S-H, Kim S-H, Yoon HJ, Shin DH, Park SS, Kim Y-S, et al. GSTT1 and GSTM1 null mutations and adverse reactions induced by antituberculosis drugs in Koreans. Tuberculosis (Edinb). 2010;90(1): 39-43.

19. McDonagh E, Whirl‐Carrillo M, Altman R, Klein T. Enabling the curation of your pharmacogenetic study. Clin Pharmacol Ther. 2015;97(2): 116-119.

20. Wain HM, Bruford EA, Lovering RC, Lush MJ, Wright MW, Povey S. Guidelines for human gene nomenclature. Genomics. 2002;79(4): 464-470.

21. Wain HM, Lush M, Ducluzeau F, Povey S. Genew: the human gene nomenclature database. Nucleic Acids Res. 2002;30(1): 169-171.

22. Thorn CF, Whirl‐Carrillo M, Hachad H, Johnson JA, McDonagh EM, Ratain MJ, et al. Essential characteristics of pharmacogenomics study publications. Clin Pharmacol Ther. 2019;105(1): 86-91.

23. Dandara C, Lombard Z, Du Plooy I, McLellan T, Norris SA, Ramsay M. Genetic variants in CYP (-1A2,-2C9,-2C19,-3A4 and-3A5), VKORC1 and ABCB1 genes in a black South African population: a window into diversity. Pharmacogenomics. 2011;12(12): 1663-1670.

24. Sherry ST, Ward M-H, Kholodov M, Baker J, Phan L, Smigielski EM, et al. dbSNP: the NCBI database of genetic variation. Nucleic Acids Res. 2001;29(1): 308-311.

25. Antonarakis SE, Group NW. Recommendations for a nomenclature system for human gene mutations. Hum Mutat. 1998;11(1): 1-3.

26. Dunnen JTd, Antonarakis SE. Mutation nomenclature extensions and suggestions to describe complex mutations: a discussion. Hum Mutat. 2000;15(1): 7-12.

27. Carr D, O'meara H, Jorgensen A, Campbell J, Hobbs M, McCann G, et al. SLCO1B1 genetic variant associated with statin‐induced myopathy: a proof‐of‐concept study using the clinical practice research datalink. Clin Pharmacol Ther. 2013;94(6): 695-701.

28. Gaedigk A, Ingelman‐Sundberg M, Miller NA, Leeder JS, Whirl‐Carrillo M, Klein TE, et al. The Pharmacogene Variation (PharmVar) Consortium: incorporation of the human cytochrome P450 (CYP) allele nomenclature database. Clin Pharmacol Ther. 2018;103(3): 399-401.

29. Gaedigk A, Sangkuhl K, Whirl‐Carrillo M, Twist GP, Klein TE, Miller NA, et al. The evolution of PharmVar. Clin Pharmacol Ther. 2019;105(1): 29.

30. Ramsey TL, Liu Q, Brennan MD. Replication of SULT4A1-1 as a pharmacogenetic marker of olanzapine response and evidence of lower weight gain in the high response group. Pharmacogenomics. 2014;15(7): 933-939.

31. Farzan N, Vijverberg SJ, Hernandez‐Pacheco N, Bel EH, Berce V, Bønnelykke K, et al. 17q21 variant increases the risk of exacerbations in asthmatic children despite inhaled corticosteroids use. Allergy. 2018;73(10): 2083-2088.

32. Hawcutt DB, Ghani A, Sutton L, Jorgensen A, Zhang E, Murray M, et al. Pharmacogenetics of warfarin in a paediatric population: time in therapeutic range, initial and stable dosing and adverse effects. Pharmacogenomics J. 2014;14(6): 542.

33. He L, Gao L, Shi Z, Li Y, Zhu L, Li S, et al. Involvement of cytochrome P450 1A1 and glutathione S-transferase P1 polymorphisms and promoter hypermethylation in the progression of anti-tuberculosis drug-induced liver injury: a case–control study. PloS One. 2015;10(3).

34. Zhou K, Bellenguez C, Spencer CC, Bennett AJ, Coleman RL, Tavendale R, et al. Common variants near ATM are associated with glycemic response to metformin in type 2 diabetes. Nat Genet. 2011;43(2): 117.

35. Laugsand EA, Fladvad T, Skorpen F, Maltoni M, Kaasa S, Fayers P, et al. Clinical and genetic factors associated with nausea and vomiting in cancer patients receiving opioids. Eur J Cancer. 2011;47(11): 1682-1691.

36. Pirmohamed M, Park K, Williamson P, Smyth R, Hawcutt D, Smyth A, et al. Protocol: MAGIC study. Molecular genetics of adverse drug reactions in paediatric patients [unpublished]. 2011.

37. McDonough CW, Gong Y, Padmanabhan S, Burkley B, Langaee TY, Melander O, et al. Pharmacogenomic association of nonsynonymous SNPs in SIGLEC12, A1BG, and the selectin region and cardiovascular outcomes. Hypertension. 2013;62(1): 48-54.

38. Thiesen S, Yin P, Jorgensen AL, Zhang JE, Manzo V, McEvoy ML, et al. TPMT, COMT and ACYP2 genetic variants in paediatric cancer patients with cisplatin-induced ototoxicity. Pharmacogenet Genomics. 2017;27(6): 213.

39. Giannoudis A, Wang L, Jorgensen AL, Xinarianos G, Davies A, Pushpakom S, et al. The hOCT1 SNPs M420del and M408V alter imatinib uptake and M420del modifies clinical outcome in imatinib-treated chronic myeloid leukemia. Blood. 2013;121(4): 628-637.

40. Musumba C, Jorgensen A, Sutton L, Van Eker D, Zhang E, O'Hara N, et al. CYP2C19* 17 gain‐of‐function polymorphism is associated with peptic ulcer disease. Clin Pharmacol Ther. 2013;93(2): 195-203.

41. Leschziner G, Jorgensen AL, Pirmohamed M, Williamson PR, Marson AG, Coffey AJ, et al. Clinical factors and ABCB1 polymorphisms in prediction of antiepileptic drug response: a prospective cohort study. Lancet Neurol. 2006;5(8): 668-676.

42. Stephens M, Smith NJ, Donnelly P. A new statistical method for haplotype reconstruction from population data. Am J Hum Genet. 2001;68(4): 978-989.

43. Marchini J, Band G. SNPTEST [Available from: <https://mathgen.stats.ox.ac.uk/genetics_software/snptest/snptest.html>.

44. Mefford J, Witte JS. The covariate's dilemma. PLoS Genet. 2012;8(11): e1003096.

45. Pirinen M, Donnelly P, Spencer CC. Including known covariates can reduce power to detect genetic effects in case-control studies. Nat Genet. 2012;44(8): 848.

46. Zaitlen N, Lindström S, Pasaniuc B, Cornelis M, Genovese G, Pollack S, et al. Informed conditioning on clinical covariates increases power in case-control association studies. PLoS Genet. 2012;8(11): e1003032.

47. Zaitlen N, Paşaniuc B, Patterson N, Pollack S, Voight B, Groop L, et al. Analysis of case–control association studies with known risk variants. Bioinformatics. 2012;28(13): 1729-1737.

48. Zhang H, Chatterjee N, Rader D, Chen J. Adjustment of nonconfounding covariates in case-control genetic association studies. Ann Appl Stat. 2018;12(1): 200-221.

49. Jorgensen AL, Hughes DA, Hanson A, Van Eker D, Toh CH, Pirmohamed M, et al. Adherence and variability in warfarin dose requirements: assessment in a prospective cohort. Pharmacogenomics. 2013;14(2): 151-163.

50. Chen R, Wang J, Tang S, Zhang Y, Lv X, Wu S, et al. Association of polymorphisms in drug transporter genes (SLCO1B1 and SLC10A1) and anti-tuberculosis drug-induced hepatotoxicity in a Chinese cohort. Tuberculosis (Edinb). 2015;95(1): 68-74.

51. Clark TG, Altman DG, De Stavola BL. Quantification of the completeness of follow-up. Lancet. 2002;359(9314): 1309-1310.

52. Hosking L, Lumsden S, Lewis K, Yeo A, McCarthy L, Bansal A, et al. Detection of genotyping errors by Hardy–Weinberg equilibrium testing. Eur J Human Genet. 2004;12(5): 395.

53. Attia J, Thakkinstian A, D'Este C. Meta-analyses of molecular association studies: methodologic lessons for genetic epidemiology. J Clin Epidemiol. 2003;56(4): 297-303.

54. Costa GN, Magno LA, Santana CV, Konstantinovas C, Saito ST, Machado M, et al. Genetic interaction between NAT2, GSTM1, GSTT1, CYP2E1, and environmental factors is associated with adverse reactions to anti-tuberculosis drugs. Mol Diagn Ther. 2012;16(4): 241-250.

55. Fernandes DCRO, Santos NPC, Moraes MR, Braga ACO, Silva CA, Ribeiro-dos-Santos A, et al. Association of the CYP2B6 gene with anti-tuberculosis drug-induced hepatotoxicity in a Brazilian Amazon population. Int J Infect Dis. 2015;33: 28-31.

56. Kim SH, Kim SH, Yoon HJ, Shin DH, Park SS, Kim YS, et al. TNF‐α genetic polymorphism− 308G/A and antituberculosis drug‐induced hepatitis. Liver Int. 2012;32(5): 809-814.

57. Ebid A-HI, Ehab M, Ismail A, Soror S, Mahmoud MA. The influence of SLC22A1 rs622342 and ABCC8 rs757110 genetic variants on the efficacy of metformin and glimepiride combination therapy in Egyptian patients with type 2 diabetes. J Drug Assess. 2019(just-accepted): 1-1.

58. Ardlie KG, Kruglyak L, Seielstad M. Patterns of linkage disequilibrium in the human genome. 2002:299.

59. Ng C-S, Hasnat A, Al Maruf A, Ahmed MU, Pirmohamed M, Day CP, et al. N-acetyltransferase 2 (NAT2) genotype as a risk factor for development of drug-induced liver injury relating to antituberculosis drug treatment in a mixed-ethnicity patient group. European journal of clinical pharmacology. 2014;70(9): 1079-1086.

60. Higashi MK, Veenstra DL, Kondo LM, Wittkowsky AK, Srinouanprachanh SL, Farin FM, et al. Association between CYP2C9 genetic variants and anticoagulation-related outcomes during warfarin therapy. JAMA. 2002;287(13): 1690-1698.

61. Kim K-M, Kim H, Lim SH, Cheong SH, Choi E-J, Kang H, et al. Effect of genetic polymorphisms of OPRM1, ABCB1, CYP3A4/5 on postoperative fentanyl consumption in Koren gynecological patients. Int J Clin Pharmacol Ther. 2013;51(5): 383-392.

62. Cecile A, Janssens W, John P, Ioannidis A, van Duijn CM, Little J, et al. Strengthening the reporting of Genetic RIsk Prediction Studies: the GRIPS statement. Eur J Epidemiol. 2011;26(4): 255.

63. Janssens ACJ, Ioannidis JP, Bedrosian S, Boffetta P, Dolan SM, Dowling N, et al. Strengthening the reporting of genetic risk prediction studies (GRIPS): explanation and elaboration. Eur J Epidemiol. 2011;26(4): 313.

64. International Warfarin Pharmacogenetics Consortium. Estimation of the warfarin dose with clinical and pharmacogenetic data. New Engl J Med. 2009;360(8): 753-764.
