# Supplementary material for: STrengthening the Reporting Of Pharmacogenetic Studies: Development of the STROPS guideline
Source: PLoS Med. 2020 Sep 21;17(9):e1003344. doi: 10.1371/journal.pmed.1003344 (PMC7505422; doi:10.1371/journal.pmed.1003344)
Supplement: S1 Presentation — (PDF) [file pmed.1003344.s008.pdf]

STROPS

(Strengthening The Reporting Of Pharmacogenetic Studies)

CONSENSUS MEETING: 7TH NOVEMBER 2019

Attendees

Steering committee
Marty Richardson
Andrea Jorgensen
Jamie Kirkham
Kerry Dwan
Gerry Davies
Derek Sloan

Expert panel
Martin H. Maurer
Chakradhara Rao S Uppugunduri
Aris Saoulidis
Juan E. Megías Vericat

STROPS protocol

Open access Protocol

BMJ Open

Protocol for the development of the STrengthening the Reporting Of Pharmacogenetic Studies (STROPS) guideline: checklist of items for reporting pharmacogenetic studies

Marty Richardson,[1] Jamie J Kirkham,[1] Kerry M Dwan,[2] Derek J Sloan,[3] Geraint Davies,[4] Andrea Jorgensen[1]

To cite: Richardson M, Kirkham JJ, Dwan KM, *et al*. Protocol for the development of the STrengthening the Reporting Of Pharmacogenetic Studies (STROPS) guideline: checklist of items for reporting pharmacogenetic studies. *BMJ Open* 2019;**9**:e030212. doi:10.1136/bmjopen-2019-030212

► Prepublication history and additional material for this paper are available online. To view these files, please visit the journal online (http://dx.doi.org/10.1136/bmjopen-2018-

ABSTRACT

Introduction Large sample sizes are often required to detect statistically significant associations between pharmacogenetic markers and treatment response. Meta-analysis may be performed to synthesise data from several studies, increasing sample size and consequently power to detect significant genetic effects. However, performing robust synthesis of data from pharmacogenetic studies is often challenging due to poor reporting of key data in study reports. There is currently no guideline for the reporting of pharmacogenetic studies. The aim of this project is to develop the STrengthening the Reporting Of Pharmacogenetic Studies (STROPS) guideline. The STROPS guideline will facilitate the conduct of high-quality meta-analyses and thus improve the power to detect genetic associations.

Strengths and limitations of this study

- We will conduct our project using methodology proposed by the EQUATOR (Enhancing the QUAlity and Transparency Of health Research) network for the development of reporting guidelines.
- The Delphi survey will enable us to gain information about the opinions of a wide group of participants.
- Our study design is limited by the fact that the consensus meeting will involve only the six members of the Steering Committee and one or two representatives from the key stakeholder groups and will be conducted via conference call.

[illegible]

Development Of STROPS Guideline

Five stages:

1. Establish a preliminary checklist of reporting items
2. Conduct a Delphi survey
3. **Hold a consensus meeting**
4. Develop and publish the reporting guideline and explanatory document
5. Dissemination activities

Delphi: Stakeholder Groups

Primary researchers

- Members of pharmacogenetics networks, such as the Pharmacogenomics Research Network (PGRN) and the UK Pharmacogenetics and Stratified Medicine Network

Systematic reviewers

- Authors of systematic reviews of pharmacogenetics studies

Journal editors

- Editors of pharmacogenetic journals

Delphi Process: Round 1

- Participants score each item with regards to the importance of including this item in the STROPS guideline
- Possible to suggest additional items

Importance									Unable to score
Not important to include in the guideline			Important but not critical to include in the guideline			Critical to include in the guideline			
1	2	3	4	5	6	7	8	9	

Delphi Process: Round 2

Participants are shown how each stakeholder group scored each item (and their own Round 1 response) and are asked to re-score the item

Your score from Round 1 is highlighted in yellow.
The percentage of people providing scores is shown above each row.

Stakeholder 1 group is represented by this background colour
Stakeholder 2 group is represented by this background colour
Stakeholder 3 group is represented by this background colour

Outcome	Number of people scoring this outcome	Not important			Important but not critical			Critical			Unable to score
		1	2	3	4	5	6	7	8	9	
**Methods: Variables**											
18. Provide justification for choice of outcomes.	2	50%	0%	0%	0%	0%	0%	0%	50%	0%	
	1	0%	0%	0%	0%	0%	0%	0%	0%	100%	
	0	0%	0%	0%	0%	0%	0%	0%	0%	0%	

Consensus:

A stakeholder group reached consensus for an item if at least 70% of individuals scored the item as critical for inclusion in the guideline (scores 7 – 9) in Round 2

Responses

Round 1 Responses (n=71)

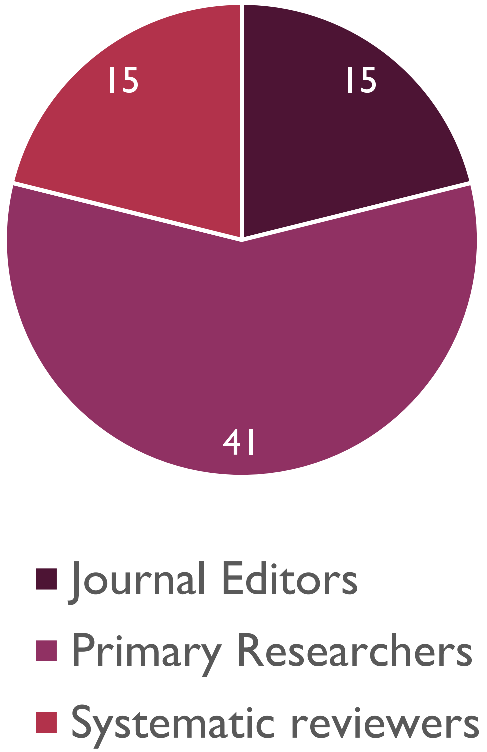

Round 2 Responses (n=52)

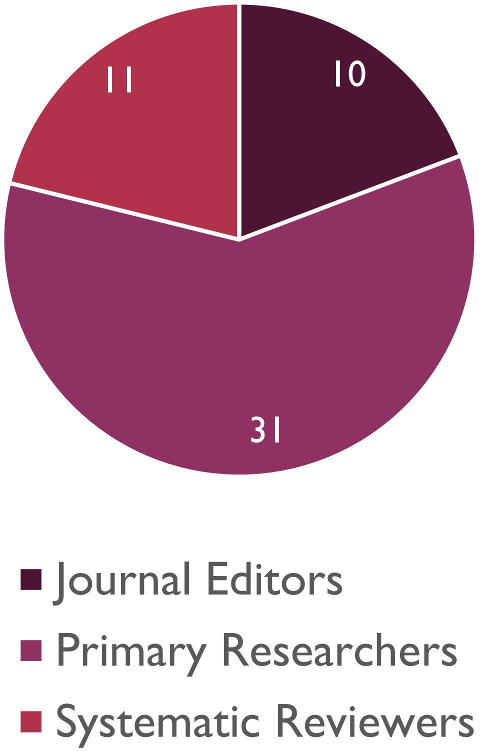

Attrition Bias: Overall

All stakeholder groups

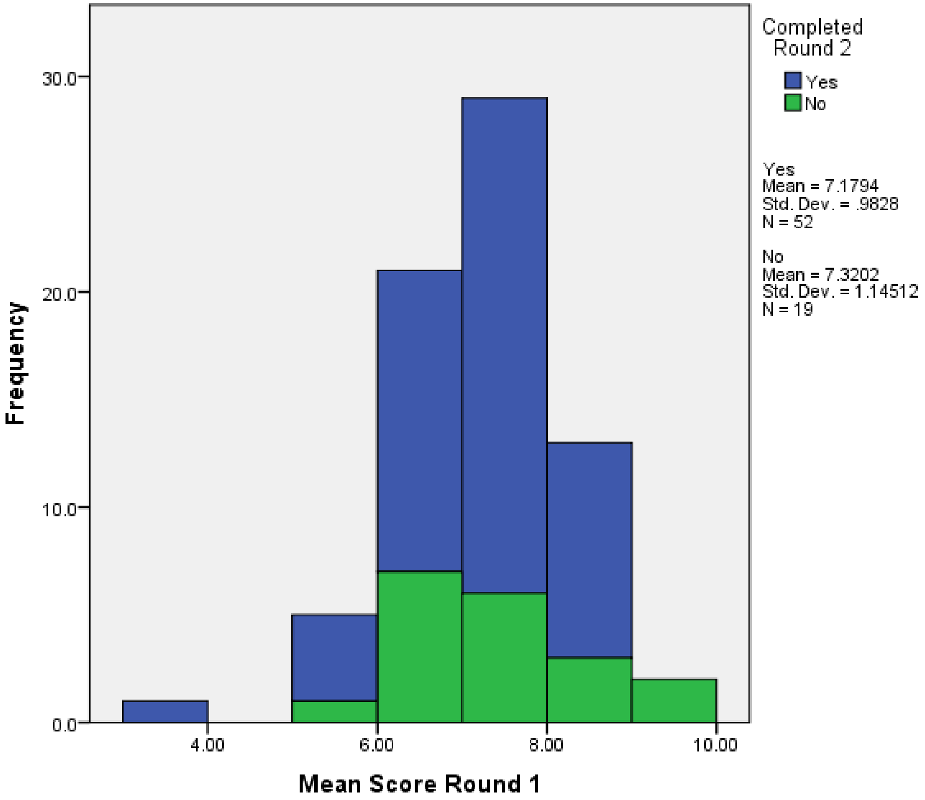

Attrition Bias: Stakeholder Groups

Journal Editors

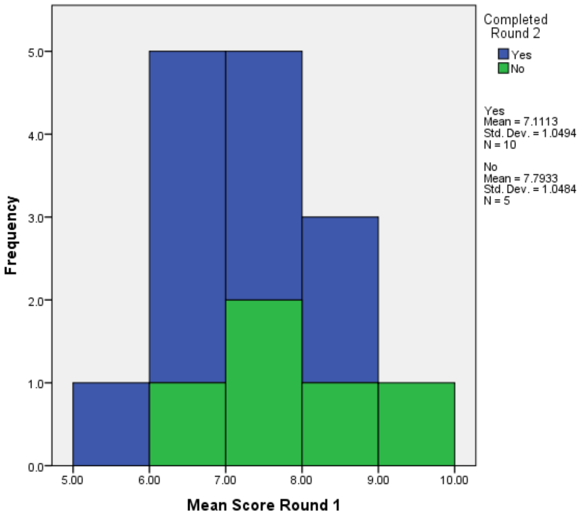

Primary Researchers

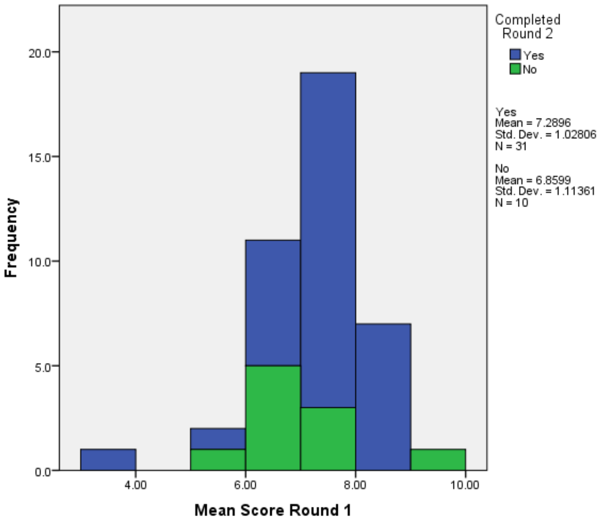

Systematic Reviewers

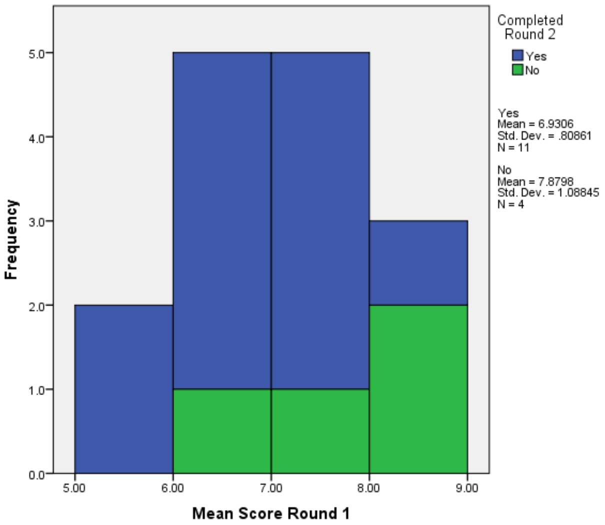

Changes In Score (R1 to R2)

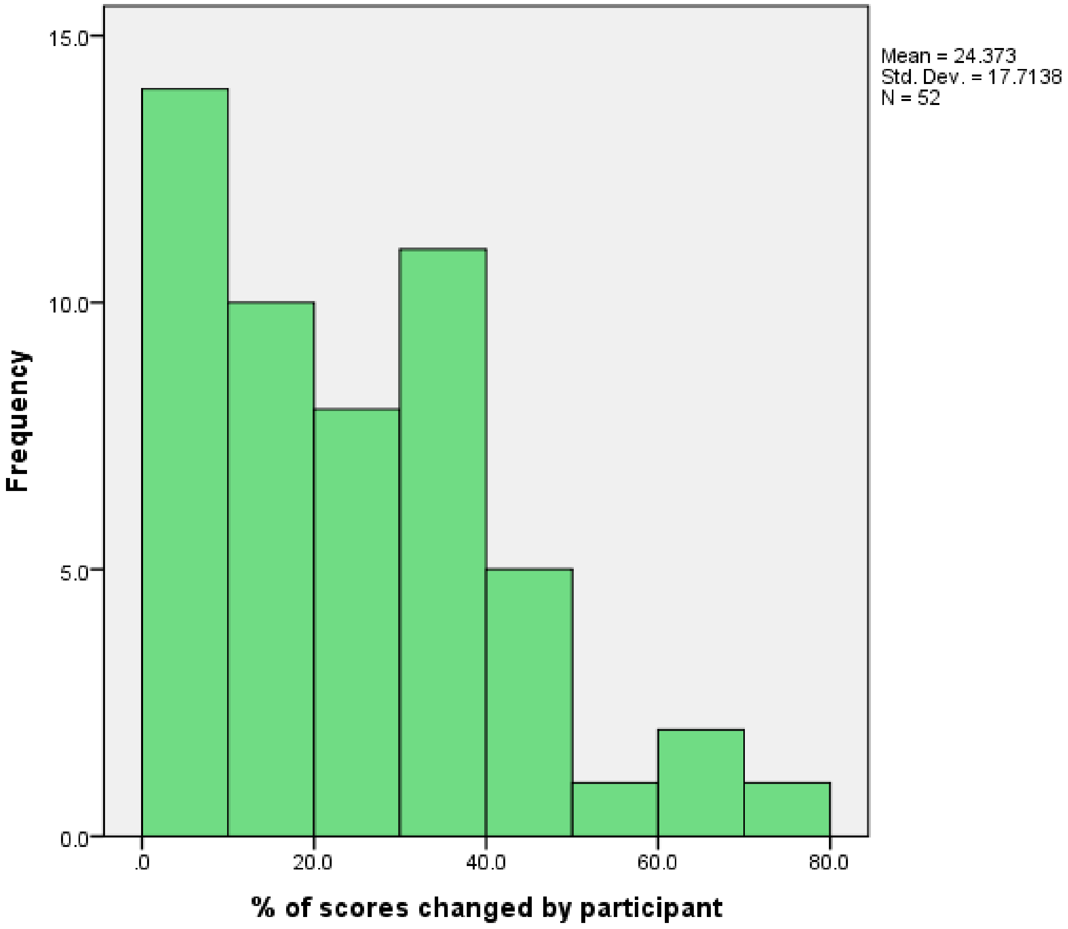

Round 2 Delphi Results

STROPS reporting guideline items

Category	#	Criteria	Help text	Consensus
**Title and abstract**				
	1	Indicate the study's pharmacogenetic design in the title and the abstract.	N/A	1 group
	2	Provide in the abstract an informative and balanced summary of what was done and what was found.	Provide the key information that enables readers to understand the research question, methods, results and conclusions of the study.	3 groups
**Introduction**				
Background/ rationale	3	Explain the scientific background and rationale for the investigation being reported.	Provide the rationale for conducting the pharmacogenetic study in the context of existing research in this health area, i.e. what is known on a topic and what gaps in current knowledge are addressed by the study.	3 groups
	4	Provide reasons for choosing the genes and SNPs genotyped.	Explain how the investigated genes and SNPs were chosen, with reference to relevant functional/animal studies, previous association studies, and any procedures used such as the "tagging SNP" approach, or assessing the likelihood of each individual SNP affecting the gene function with priority given to those with the most likely functional effect.	3 groups
	5	If reasons for (4) include previous association studies, provide key details from these studies (effect size and standard error/confidence interval).	N/A	1 group
Objectives	6	State specific objectives, including any pre-specified hypotheses.	Provide the objectives for the study, specifying the relevant population, genetic variants, drugs and outcomes.	3 groups
	7	State if the study is the first report of a pharmacogenetic association, a replication effort, or both.	N/A	2 groups
**Methods**				
Study design	8	Present key elements of study design early in the paper.	Present key elements of study design so that readers can understand the basics of the study, e.g. for a cohort study: state that the study used a cohort design, describe the group of people that comprised the cohort and the time period for which they were followed; for a case-control design: state that the study used a case-control design, describe the cases and controls and their source population; for a post-hoc pharmacogenetic analysis of a RCT: state how the subjects included in the analysis were chosen, including which arm of the RCT they were from.	3 groups
Setting	9	Describe the setting, locations and relevant dates, including periods of recruitment, follow-up, and data collection.	Provide sufficient information to enable readers to assess the context and generalisability of a study's results.	0 groups
Participants	10	Cohort study – Give the eligibility criteria, and the sources and methods of selection of participants. Describe methods of follow-up.	Provide sufficiently detailed descriptions of the study participants to help readers understand the applicability of the results. Include details of follow-up procedures, including any procedures to minimise non-response/loss to follow-up.	3 groups

Items Where All Groups Reached Consensus

- N=46
- Automatically included in the guideline
- Any objections?

Items Where No Groups Reached Consensus

- N=16
- Automatically excluded from the guideline
- Any objections?

Polling

- Where necessary, we will vote on inclusion/exclusion of items
- ttpoll.eu
- Session ID: Toosey

Title And Abstract

Item	Journal Editors	Primary Researchers	Systematic Reviewers
**1. Indicate the study's pharmacogenetic design in the title and the abstract.**	50%	70%	55%

Consensus in 1 group

"It is not practical to always include key features of study design in the title"
Primary Researcher

- Discussion and voting

A. Include
B. Exclude

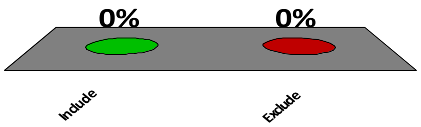

Title And Abstract

Item	Journal Editors	Primary Researchers	Systematic Reviewers
**2. Provide in the abstract an informative and balanced summary of what was done and what was found.**	100%	100%	91%

Consensus in 3 groups

- Automatic inclusion

A. Include

B. Exclude

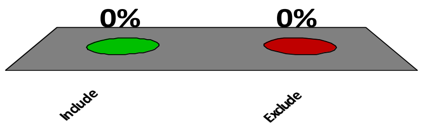

Introduction: Background/Rationale

Item	Journal Editors	Primary Researchers	Systematic Reviewers
**3. Explain the scientific background and rationale for the investigation being reported.**	90%	97%	91%

Consensus in 3 groups

- Automatic inclusion

A. Include

B. Exclude

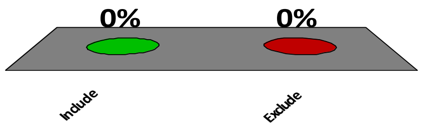

Introduction: Background/Rationale

Item	Journal Editors	Primary Researchers	Systematic Reviewers
**4. Provide reasons for choosing the genes and SNPs genotyped.**	90%	97%	91%

Consensus in 3 groups

- Automatic inclusion

A. Include

B. Exclude

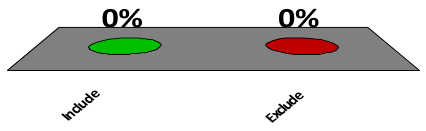

Introduction: Background/Rationale

Item	Journal Editors	Primary Researchers	Systematic Reviewers
**5. If reasons for (4) include previous association studies, provide key details from these studies (effect size and standard error/confidence interval).**	70%	45%	27%

Consensus in 1 group

- Discussion and voting

A. Include
B. Exclude

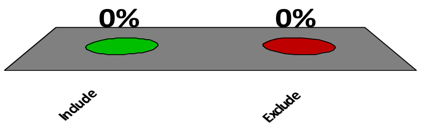

Introduction: Objectives

Item	Journal Editors	Primary Researchers	Systematic Reviewers
**6. State specific objectives, including any pre-specified hypotheses.**	70%	94%	91%

Consensus in 3 groups

- Automatic inclusion

A. Include

B. Exclude

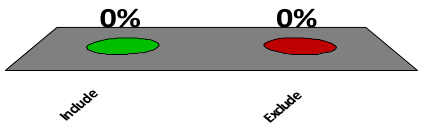

Introduction: Objectives

Item	Journal Editors	Primary Researchers	Systematic Reviewers
**7. State if the study is the first report of a pharmacogenetic association, a replication effort, or both.**	60%	74%	91%

Consensus in 2 groups

- Discussion and voting

A. Include

B. Exclude

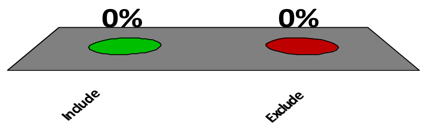

Methods: Study Design

Item	Journal Editors	Primary Researchers	Systematic Reviewers
**8. Present key elements of study design early in the paper.**	70%	74%	91%

Consensus in 3 groups

- Automatic inclusion

A. Include
B. Exclude

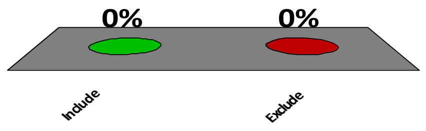

Methods: Setting

Item	Journal Editors	Primary Researchers	Systematic Reviewers
**9. Describe the setting, locations and relevant dates, including periods of recruitment, follow-up, and data collection.**	40%	61%	36%

Consensus in 0 groups

- Automatic exclusion

A. Include

B. Exclude

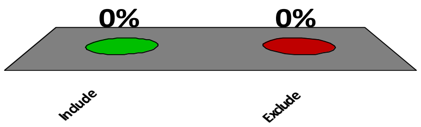

Methods: Participants

Item	Journal Editors	Primary Researchers	Systematic Reviewers
**10. Cohort study – Give the eligibility criteria, and the sources and methods of selection of participants. Describe methods of follow-up.**	90%	94%	91%

Consensus in 3 groups

- Automatic inclusion

A. Include

B. Exclude

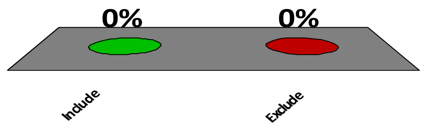

Methods: Participants

Item	Journal Editors	Primary Researchers	Systematic Reviewers
**11. Case-control study – Give the eligibility criteria, and the sources and methods of case ascertainment and control selection. State whether true controls or population controls were used. Give the rationale for the choice of cases and controls.**	90%	94%	91%

Consensus in 3 groups

- Automatic inclusion

A. Include

B. Exclude

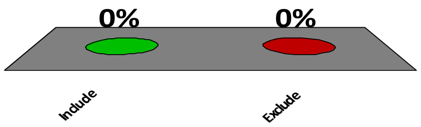

Methods: Participants

Item	Journal Editors	Primary Researchers	Systematic Reviewers
**12. Cross-sectional study – Give the eligibility criteria, and the sources and methods of selection of participants.**	90%	94%	91%

Consensus in 3 groups

- Automatic inclusion

A. Include

B. Exclude

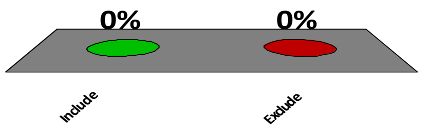

Methods: Participants

Item	Journal Editors	Primary Researchers	Systematic Reviewers
**13. Report the drug and regime participants were exposed to, and the length of exposure.**	90%	90%	100%

Consensus in 3 groups

- Automatic inclusion

A. Include

B. Exclude

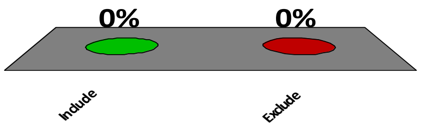

Methods: Participants

Item	Journal Editors	Primary Researchers	Systematic Reviewers
**14. Cohort study – For matched studies, give matching criteria and number in each genotype group.**	100%	94%	91%

Consensus in 3 groups

- Automatic inclusion

A. Include

B. Exclude

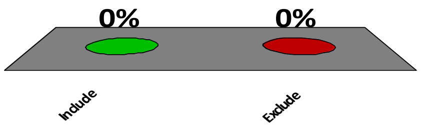

Methods: Participants

Item	Journal Editors	Primary Researchers	Systematic Reviewers
**15. Case-control study – For matched studies, give matching criteria and the number of controls per case.**	100%	94%	91%

Consensus in 3 groups

- Automatic inclusion

A. Include

B. Exclude

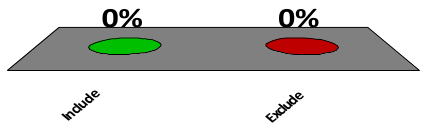

Methods: Participants

Item	Journal Editors	Primary Researchers	Systematic Reviewers
**16. Give information on the criteria and methods for selection of subsets of participants from a larger study, when relevant.**	70%	90%	91%

Consensus in 3 groups

- Automatic inclusion

A. Include

B. Exclude

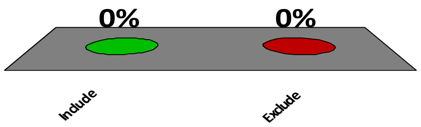

Methods: Participants

Item	Journal Editors	Primary Researchers	Systematic Reviewers
**17. If other publications report results for the same patient cohort, or a subset of the patient cohort, provide information on this patient cohort overlap and references to the relevant publications.**	50%	74%	91%

Consensus in 2 groups

- Discussion and voting

A. Include

B. Exclude

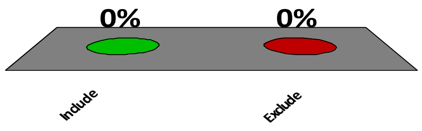

Methods: Participants

Item	Journal Editors	Primary Researchers	Systematic Reviewers
**18. Report disease/clinical indication of patients using a standardised ontology.**	44%	65%	73%

Consensus in 1 group

- Discussion and voting

A. Include

B. Exclude

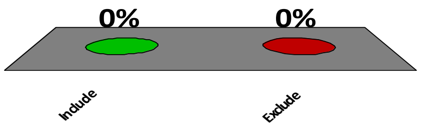

Methods: Participants

Item	Journal Editors	Primary Researchers	Systematic Reviewers
**19. Confirm whether patients were blinded to their genotyping result.**	80%	52%	64%

Consensus in 1 group

- Discussion and voting

A. Include

B. Exclude

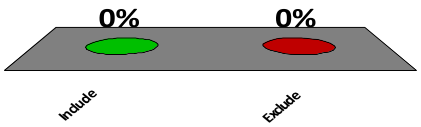

Methods: Variables

Item	Journal Editors	Primary Researchers	Systematic Reviewers
**20. Provide justification for choice of outcomes.**	80%	84%	82%

Consensus in 3 groups

- Automatic inclusion

A. Include

B. Exclude

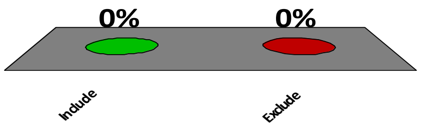

Methods: Variables

Item	Journal Editors	Primary Researchers	Systematic Reviewers
**21. Clearly define all outcomes, potential confounders, and effect modifiers. Give diagnostic criteria, if applicable.**	90%	87%	100%

Consensus in 3 groups

- Automatic inclusion

A. Include

B. Exclude

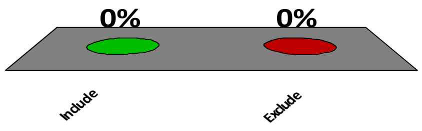

Methods: Variables

Item	Journal Editors	Primary Researchers	Systematic Reviewers
**22. Clearly define genetic exposures (genetic variants) using a widely-used nomenclature system. Identify variables likely to be associated with population stratification (confounding by ethnic origin).**	90%	97%	91%

Consensus in 3 groups

- Automatic inclusion

A. Include

B. Exclude

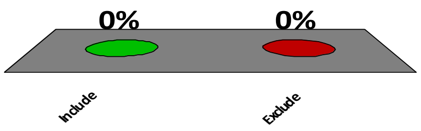

Methods: Variables

Item	Journal Editors	Primary Researchers	Systematic Reviewers
**23. Report the rs number of each genotyped SNP.**	90%	94%	82%

Consensus in 3 groups

"Can combine this with item 22"
Primary Researcher

- Automatic inclusion

A. Include
B. Exclude

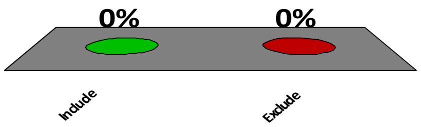

Methods: Variables

Item	Journal Editors	Primary Researchers	Systematic Reviewers
**24. Report whether the outcomes measured (including definitions) are in line with core/preferred outcome sets for the particular topic of interest.**	80%	66%	64%

Consensus in 1 group

- Discussion and voting

A. Include

B. Exclude

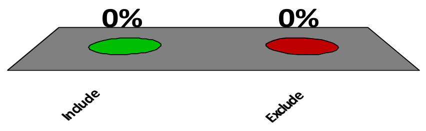

Methods: Variables

Item	Journal Editors	Primary Researchers	Systematic Reviewers
**25. Clearly state how haplotypes or star alleles were defined.**	80%	73%	82%

Consensus in 3 groups

- Automatic inclusion

A. Include

B. Exclude

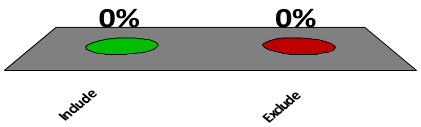

Methods: Variables

Item	Journal Editors	Primary Researchers	Systematic Reviewers
**26. Clearly state on which chromosomal strand the alleles are reported.**	60%	59%	73%

Consensus in 1 group

- Discussion and voting

A. Include

B. Exclude

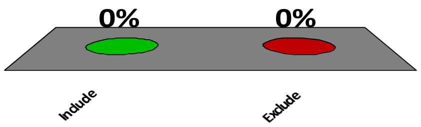

Methods: Variables

Item	Journal Editors	Primary Researchers	Systematic Reviewers
**27. If referring to the minor, wild-type or mutant allele of a variant, state which allele this is and for which given population/cohort.**	80%	67%	82%

Consensus in 2 groups

- Discussion and voting

A. Include

B. Exclude

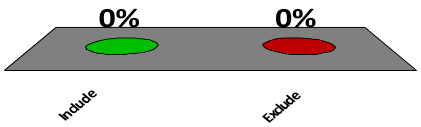

Methods: Variables

Item	Journal Editors	Primary Researchers	Systematic Reviewers
**28. If studying drug metabolites; provide references and links to structures and database identifiers.**	40%	48%	64%

Consensus in 0 groups

- Automatic exclusion

A. Include

B. Exclude

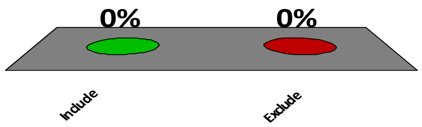

Methods: Data Sources Measurement

Item	Journal Editors	Primary Researchers	Systematic Reviewers
**29. For each variable of interest, give sources of data and details of methods of assessment (measurement). Describe comparability of assessment methods if there is more than one group.**	70%	61%	55%

Consensus in 1 group

- Discussion and voting

A. Include

B. Exclude

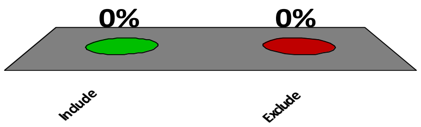

Methods: Data Sources Measurement

Item	Journal Editors	Primary Researchers	Systematic Reviewers
**30. Describe laboratory methods, including source and storage of DNA, genotyping methods and platforms (including the allele calling algorithm used, and its version), error rates and call rates. State the laboratory/centre where genotyping was done. Describe comparability of laboratory methods if there is more than one group. Specify whether genotypes were assigned using all of the data from the study simultaneously or in smaller batches.**	80%	81%	73%

Consensus in 3 groups

- Automatic inclusion

A. Include

B. Exclude

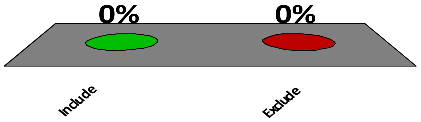

Methods: Data Sources Measurement

Item	Journal Editors	Primary Researchers	Systematic Reviewers
**31. If study is case-control, confirm whether patients were genotyped in mixed batches.**	30%	58%	36%

Consensus in 0 groups

- Automatic exclusion

A. Include

B. Exclude

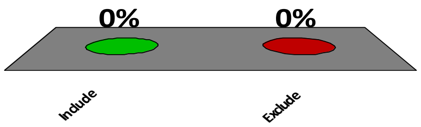

Methods: Data Sources Measurement

Item	Journal Editors	Primary Researchers	Systematic Reviewers
**32. Confirm whether genotyping personnel were blinded to outcome status.**	40%	55%	46%

Consensus in 0 groups

- Automatic exclusion

A. Include

B. Exclude

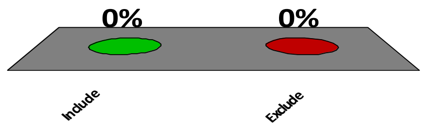

Methods: Data Sources Measurement

Item	Journal Editors	Primary Researchers	Systematic Reviewers
**33. Describe the primers used.**	50%	29%	9%

Consensus in 0 groups

"Out of date concept as most studies of this type involve chips or real time PCR where primers are not particularly relevant" - Primary Researcher

"Primers might be proprietary" - Primary Researcher

- Automatic exclusion

A. Include

B. Exclude

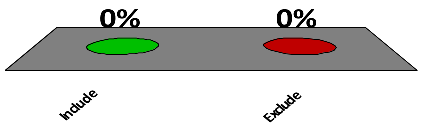

Methods: Data Sources Measurement

Item	Journal Editors	Primary Researchers	Systematic Reviewers
**34. Describe genotype quality control methods.**	90%	52%	46%

Consensus in 1 group

- Discussion and voting

A. Include
B. Exclude

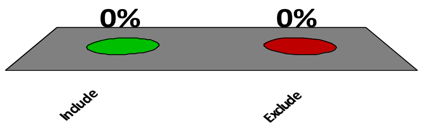

Methods: Data Sources Measurement

Item	Journal Editors	Primary Researchers	Systematic Reviewers
**35. Describe findings of genotype quality control methods.**	60%	42%	46%

Consensus in 0 groups

- Automatic exclusion

A. Include

B. Exclude

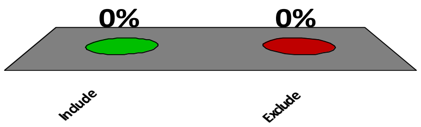

Methods: Bias

Item	Journal Editors	Primary Researchers	Systematic Reviewers
**36. Describe any efforts to address potential sources of bias.**	60%	84%	55%

Consensus in 1 group

- Discussion and voting

A. Include

B. Exclude

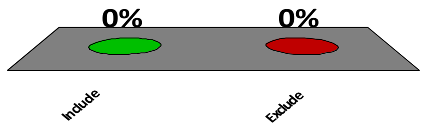

Methods: Bias

Item	Journal Editors	Primary Researchers	Systematic Reviewers
**37. For quantitative outcome variables, specify if any investigation of potential bias resulting from pharmacotherapy was undertaken. If relevant, describe the nature and magnitude of the potential bias, and explain what approach was used to deal with this.**	90%	73%	73%

Consensus in 3 groups

- Automatic inclusion

A. Include

B. Exclude

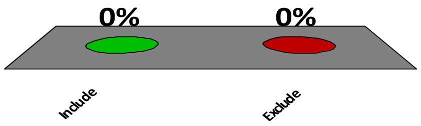

Methods: Bias

Item	Journal Editors	Primary Researchers	Systematic Reviewers
**38. Report how adherence to treatment was assessed, and report the results of the assessment.**	90%	81%	73%

Consensus in 3 groups

- Automatic inclusion

A. Include

B. Exclude

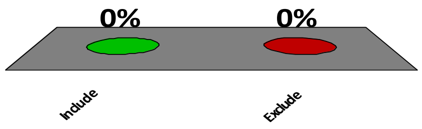

Methods: Study Size

Item	Journal Editors	Primary Researchers	Systematic Reviewers
**39. Explain how the study size was arrived at, or provide details of the a priori power to detect effect sizes of varying degrees.**	90%	84%	73%

Consensus in 3 groups

- Automatic inclusion

A. Include

B. Exclude

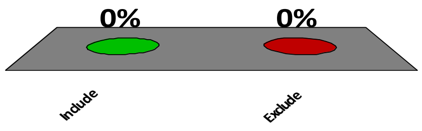

Methods: Quantitative Variables

Item	Journal Editors	Primary Researchers	Systematic Reviewers
**40. Explain how quantitative variables (confounders and effect modifiers) were handled in the analyses. If applicable, describe which groupings were chosen, and why.**	80%	87%	73%

Consensus in 3 groups

- Automatic inclusion

A. Include

B. Exclude

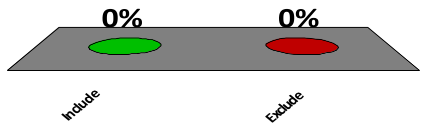

Methods: Quantitative Variables

Item	Journal Editors	Primary Researchers	Systematic Reviewers
**41. If applicable, describe how effects of treatment on quantitative outcome variables were dealt with.**	90%	77%	46%

Consensus in 2 groups

"Is this not the same as item 37?" - Primary Researcher

- Discussion and voting

A. Include

B. Exclude

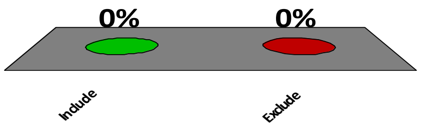

Methods: Statistical Methods

Item	Journal Editors	Primary Researchers	Systematic Reviewers
**42. Describe all statistical methods, including those used to control for confounding.**	80%	97%	82%

Consensus in 3 groups

- Automatic inclusion

A. Include

B. Exclude

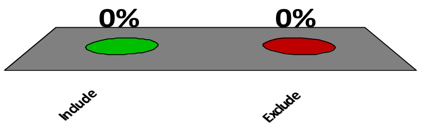

Methods: Statistical Methods

Item	Journal Editors	Primary Researchers	Systematic Reviewers
**43. State software version used and options (or settings) chosen.**	60%	42%	18%

Consensus in 0 groups

- Automatic exclusion

A. Include

B. Exclude

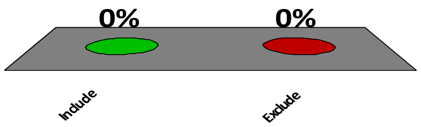

Methods: Statistical Methods

Item	Journal Editors	Primary Researchers	Systematic Reviewers
**44. Describe any methods used to examine subgroups and interactions.**	60%	87%	64%

Consensus in 1 group

- Discussion and voting

A. Include

B. Exclude

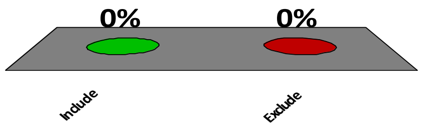

Methods: Statistical Methods

Item	Journal Editors	Primary Researchers	Systematic Reviewers
**45. Explain how missing data were addressed.**	80%	81%	73%

Consensus in 3 groups

- Automatic inclusion

A. Include

B. Exclude

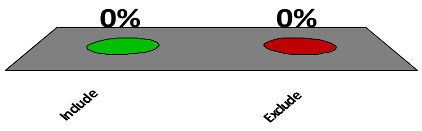

Methods: Statistical Methods

Item	Journal Editors	Primary Researchers	Systematic Reviewers
**46. Report any methods used to assess the assumption of missingness at random and the finding of such assessments.**	70%	66%	46%

Consensus in 1 group

- Discussion and voting

A. Include

B. Exclude

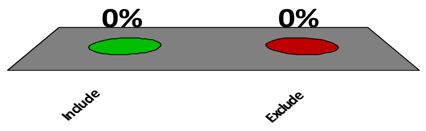

Methods: Statistical Methods

Item	Journal Editors	Primary Researchers	Systematic Reviewers
**47. Cohort study – If applicable, explain how loss to follow-up was addressed.**	60%	77%	36%

Consensus in 1 group

- Discussion and voting

A. Include

B. Exclude

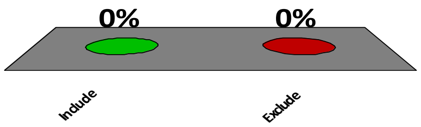

Methods: Statistical Methods

Item	Journal Editors	Primary Researchers	Systematic Reviewers
**48. Case-control study – If applicable, explain how matching of cases and controls was addressed.**	70%	83%	27%

Consensus in 2 groups

- Discussion and voting

A. Include

B. Exclude

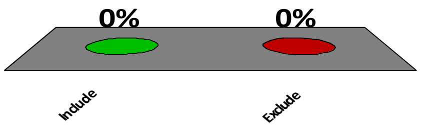

Methods: Statistical Methods

Item	Journal Editors	Primary Researchers	Systematic Reviewers
**49. Cross-sectional study – If applicable, describe analytical methods taking account of sampling strategy.**	80%	69%	27%

Consensus in 1 group

- Discussion and voting

A. Include
B. Exclude

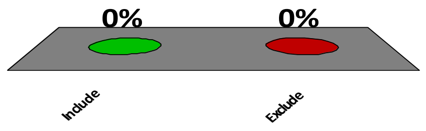

Methods: Statistical Methods

Item	Journal Editors	Primary Researchers	Systematic Reviewers
**50. Describe any sensitivity analyses.**	22%	67%	36%

Consensus in 0 groups

- Automatic exclusion

A. Include

B. Exclude

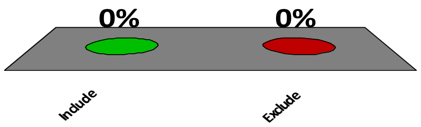

Methods: Statistical Methods

Item	Journal Editors	Primary Researchers	Systematic Reviewers
**51. State whether Hardy-Weinberg equilibrium was considered and, if so, how.**	90%	80%	64%

Consensus in 2 groups

- Discussion and voting

A. Include

B. Exclude

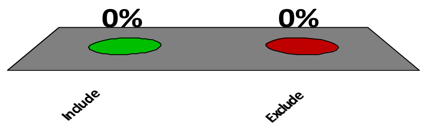

Methods: Statistical Methods

Item	Journal Editors	Primary Researchers	Systematic Reviewers
**52. Where HWE test is undertaken, quote the p-value threshold applied to determine deviation from HWE.**	80%	77%	36%

Consensus in 2 groups

- Discussion and voting

A. Include

B. Exclude

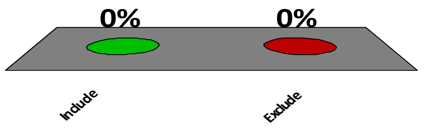

Methods: Statistical Methods

Item	Journal Editors	Primary Researchers	Systematic Reviewers
**53. Describe any methods used for inferring genotypes or haplotypes.**	70%	90%	55%

Consensus in 2 groups

- Discussion and voting

A. Include

B. Exclude

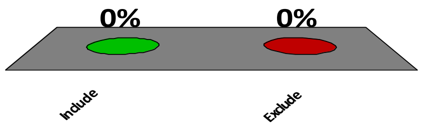

Methods: Statistical Methods

Item	Journal Editors	Primary Researchers	Systematic Reviewers
**54. Describe any methods used to assess or address population stratification.**	60%	97%	64%

Consensus in 1 group

- Discussion and voting

A. Include

B. Exclude

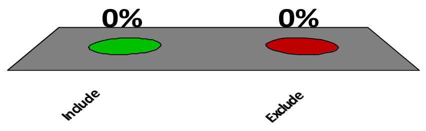

Methods: Statistical Methods

Item	Journal Editors	Primary Researchers	Systematic Reviewers
**55. Describe any methods used to assess and correct for relatedness among subjects. Report results of assessments for relatedness.**	60%	81%	27%

Consensus in 1 group

- Discussion and voting

A. Include

B. Exclude

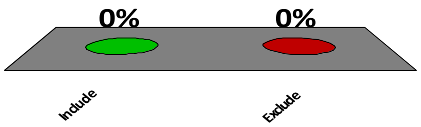

Methods: Statistical Methods

Item	Journal Editors	Primary Researchers	Systematic Reviewers
**56. Describe any assumptions made regarding mode of inheritance.**	60%	73%	36%

Consensus in 1 group

- Discussion and voting

A. Include

B. Exclude

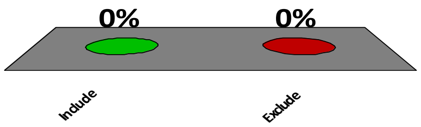

Methods: Statistical Methods

Item	Journal Editors	Primary Researchers	Systematic Reviewers
**57. Provide justification for assumption of mode of inheritance or if no mode is assumed.**	60%	77%	55%

Consensus in 1 group

- Discussion and voting

A. Include

B. Exclude

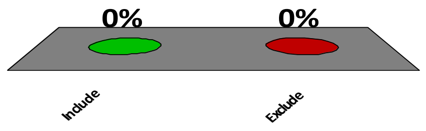

Methods: Statistical Methods

Item	Journal Editors	Primary Researchers	Systematic Reviewers
**58a. Describe any methods used to address multiple comparisons or to control risk of false positive results due to investigating multiple genetic variants.**	90%	97%	82%

Consensus in 3 groups

- Automatic inclusion

A. Include

B. Exclude

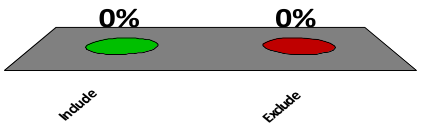

Methods: Statistical Methods

Item	Journal Editors	Primary Researchers	Systematic Reviewers
**58b. Describe any methods used to address multiple comparisons or to control risk of false positive results due to investigating multiple outcomes.**	90%	93%	82%

Consensus in 3 groups

- Automatic inclusion

A. Include

B. Exclude

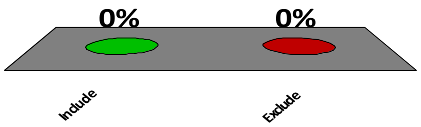

Methods: Statistical Methods

Item	Journal Editors	Primary Researchers	Systematic Reviewers
**58c. Describe any methods used to address multiple comparisons or to control risk of false positive results due to investigating multiple assumptions regarding mode of inheritance.**	50%	86%	73%

Consensus in 2 groups

- Discussion and voting

A. Include

B. Exclude

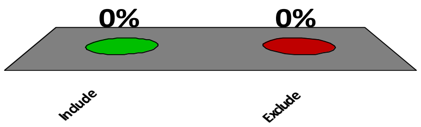

Methods: Statistical Methods

Item	Journal Editors	Primary Researchers	Systematic Reviewers
**59. Describe any methods used to adjust for extent of adherence in the analyses.**	89%	74%	50%

Consensus in 2 groups

- Discussion and voting

A. Include

B. Exclude

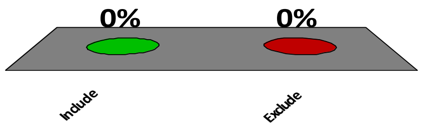

Results: Participants

Item	Journal Editors	Primary Researchers	Systematic Reviewers
**60a. Report the numbers of individuals at each stage of the study – e.g., numbers potentially eligible, examined for eligibility, confirmed eligible, included in the study, completing follow-up, and analysed.**	90%	90%	91%

Consensus in 3 groups

- Automatic inclusion

A. Include

B. Exclude

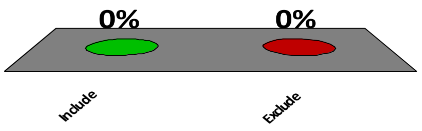

Results: Participants

Item	Journal Editors	Primary Researchers	Systematic Reviewers
**60b. Give reasons for non-participation at each stage.**	30%	58%	46%

Consensus in 0 groups

- Automatic exclusion

A. Include

B. Exclude

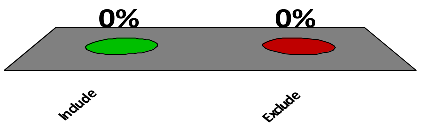

Results: Participants

Item	Journal Editors	Primary Researchers	Systematic Reviewers
**60c. Consider use of a flow diagram.**	30%	48%	36%

Consensus in 0 groups

- Automatic exclusion

A. Include

B. Exclude

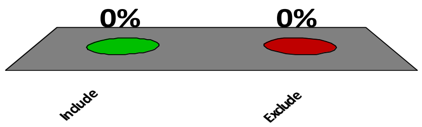

Results: Participants

Item	Journal Editors	Primary Researchers	Systematic Reviewers
**61. For each genetic variant, report numbers of individuals in whom genotyping was attempted and numbers of individuals in whom genotyping was successful.**	50%	63%	73%

Consensus in 1 group

- Discussion and voting

A. Include

B. Exclude

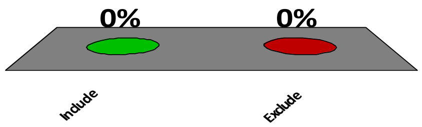

Results: SNPs

Item	Journal Editors	Primary Researchers	Systematic Reviewers
**62. Report any SNPs that were excluded from analysis, and provide reasons for these exclusions.**	50%	81%	82%

Consensus in 2 groups

- Discussion and voting

A. Include

B. Exclude

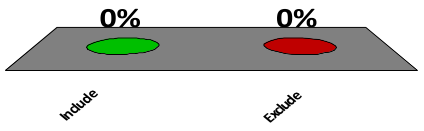

Results: Descriptive Data

Item	Journal Editors	Primary Researchers	Systematic Reviewers
**63. Give characteristics of study participants (e.g., demographic, clinical, social) and information on potential confounders.**	100%	94%	100%

Consensus in 3 groups

- Automatic inclusion

A. Include

B. Exclude

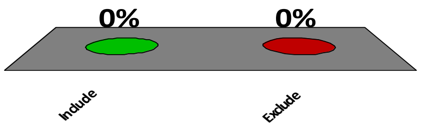

Results: Descriptive Data

Item	Journal Editors	Primary Researchers	Systematic Reviewers
**64. Indicate the number of participants with missing data for each variable of interest.**	90%	71%	91%

Consensus in 3 groups

- Automatic inclusion

A. Include

B. Exclude

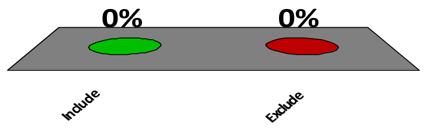

Results: Descriptive Data

Item	Journal Editors	Primary Researchers	Systematic Reviewers
**65. For a cohort study, consider giving information listed in (63) and (64) by genotype.**	30%	50%	73%

Consensus in 1 group

- Discussion and voting

A. Include

B. Exclude

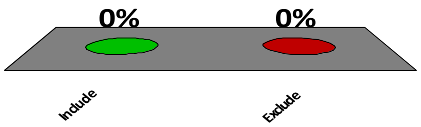

Results: Descriptive Data

Item	Journal Editors	Primary Researchers	Systematic Reviewers
**66. For a case-control study, give the information listed in (63) and (64) for cases and controls separately.**	60%	67%	64%

Consensus in 0 groups

- Automatic exclusion

A. Include

B. Exclude

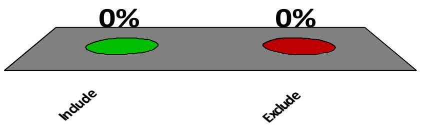

Results: Descriptive Data

Item	Journal Editors	Primary Researchers	Systematic Reviewers
**67. Report reasons for missing genotype data.**	40%	42%	36%

Consensus in 0 groups

- Automatic exclusion

A. Include
B. Exclude

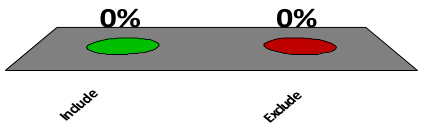

Results: Descriptive Data

Item	Journal Editors	Primary Researchers	Systematic Reviewers
**68. Cohort study – Summarize follow-up time, e.g. average and total amount.**	70%	87%	64%

Consensus in 2 groups

- Discussion and voting

A. Include

B. Exclude

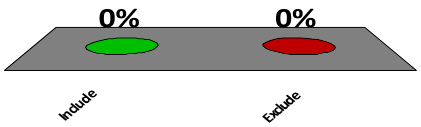

Results: Descriptive Data

Item	Journal Editors	Primary Researchers	Systematic Reviewers
**69. Where HWE tests have been undertaken, highlight SNPs that deviate from HWE.**	90%	79%	73%

Consensus in 3 groups

- Automatic inclusion

A. Include

B. Exclude

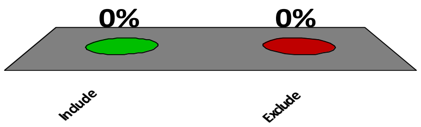

Results: Descriptive Data

Item	Journal Editors	Primary Researchers	Systematic Reviewers
**70. Where population stratification is assessed, report the results.**	90%	87%	73%

Consensus in 3 groups

- Automatic inclusion

A. Include

B. Exclude

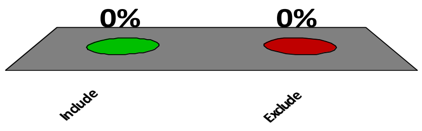

Results: Outcome Data

Item	Journal Editors	Primary Researchers	Systematic Reviewers
**71a. For a cohort study, report all outcomes (phenotypes) investigated for each genotype category over time.**	70%	80%	91%

Consensus in 3 groups

- Automatic inclusion

A. Include

B. Exclude

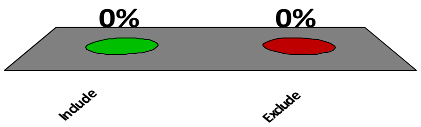

Results: Outcome Data

Item	Journal Editors	Primary Researchers	Systematic Reviewers
**71b. For a case-control study, report numbers in each genotype category for all outcomes investigated.**	90%	87%	91%

Consensus in 3 groups

- Automatic inclusion

A. Include

B. Exclude

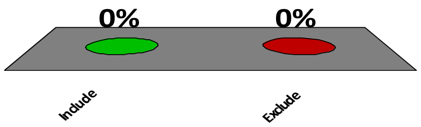

Results: Outcome Data

Item	Journal Editors	Primary Researchers	Systematic Reviewers
**71c. For a cross sectional study, report all outcomes (phenotypes) investigated for each genotype category.**	70%	90%	91%

Consensus in 3 groups

- Automatic inclusion

A. Include

B. Exclude

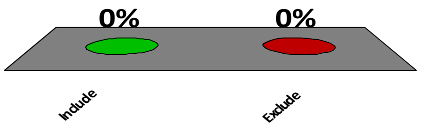

Results: Outcome Data

Item	Journal Editors	Primary Researchers	Systematic Reviewers
**72. If a study includes more than one ethnic group, provide the summary data specified in (71) per ethnic group.**	90%	84%	73%

Consensus in 3 groups

- Automatic inclusion

A. Include

B. Exclude

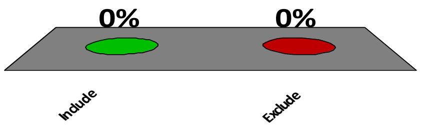

Results: Main Results

Item	Journal Editors	Primary Researchers	Systematic Reviewers
**73. Give unadjusted estimates and, if applicable, confounder-adjusted estimates and their precision (e.g., 95% confidence intervals). Make clear which confounders were adjusted for and why they were included.**	100%	97%	100%

Consensus in 3 groups

- Automatic inclusion

A. Include

B. Exclude

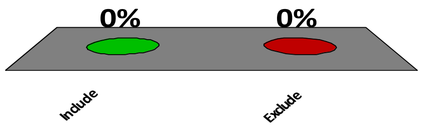

Results: Main Results

Item	Journal Editors	Primary Researchers	Systematic Reviewers
**74. Report category boundaries when continuous variables were categorised.**	70%	97%	100%

Consensus in 3 groups

- Automatic inclusion

A. Include

B. Exclude

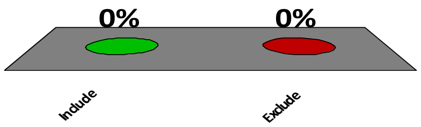

Results: Main Results

Item	Journal Editors	Primary Researchers	Systematic Reviewers
**75. If relevant, consider translating effect estimates to number needed to test to illustrate potential clinical utility of any significant findings.**	60%	62%	46%

Consensus in 0 groups

- Automatic exclusion

A. Include

B. Exclude

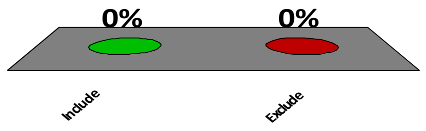

Results: Main Results

Item	Journal Editors	Primary Researchers	Systematic Reviewers
**76. Report results of any adjustments for multiple comparisons.**	80%	94%	100%

Consensus in 3 groups

- Automatic inclusion

A. Include

B. Exclude

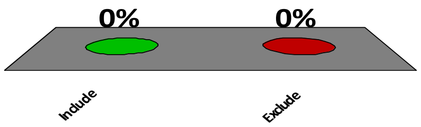

Results: Main Results

Item	Journal Editors	Primary Researchers	Systematic Reviewers
**77. Report precise p-values for all associations.**	70%	87%	91%

Consensus in 3 groups

- Automatic inclusion

A. Include

B. Exclude

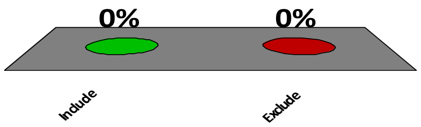

Results: Other Analyses

Item	Journal Editors	Primary Researchers	Systematic Reviewers
**78. Report other analyses done – e.g., analyses of subgroups and interactions, and sensitivity analyses.**	50%	83%	91%

Consensus in 2 groups

- Discussion and voting

A. Include

B. Exclude

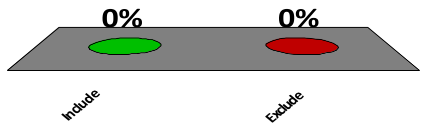

Results: Other Analyses

Item	Journal Editors	Primary Researchers	Systematic Reviewers
**79. If numerous genetic exposures (genetic variants) were examined, summarize results from all analyses undertaken.**	20%	74%	73%

Consensus in 2 groups

"Lack of association can inform other studies and is something we capture at PharmGKB when the paper has sufficient descriptions"
Systematic Reviewer

- Discussion and voting

A. Include
B. Exclude

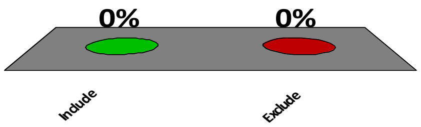

Results: Other Analyses

Item	Journal Editors	Primary Researchers	Systematic Reviewers
**80. If detailed results are available elsewhere, state how they can be accessed.**	80%	81%	82%

Consensus in 3 groups

- Automatic inclusion

A. Include

B. Exclude

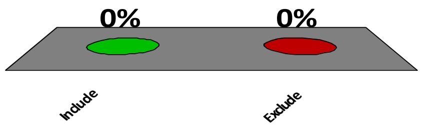

Discussion: Key Results

Item	Journal Editors	Primary Researchers	Systematic Reviewers
**81. Summarize key results with reference to study objectives.**	100%	97%	100%

Consensus in 3 groups

- Automatic inclusion

A. Include

B. Exclude

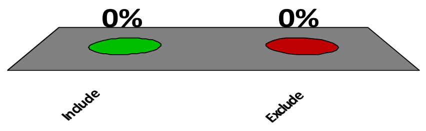

Discussion: Limitations

Item	Journal Editors	Primary Researchers	Systematic Reviewers
**82. Discuss limitations of the study, taking into account sources of potential bias or imprecision. Discuss both direction and magnitude of any potential bias.**	100%	97%	100%

Consensus in 3 groups

- Automatic inclusion

A. Include

B. Exclude

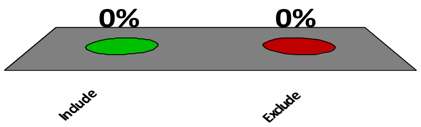

Discussion: Limitations

Item	Journal Editors	Primary Researchers	Systematic Reviewers
**83. Report on the risk of phenoconversion (genotype-phenotype mismatch) and its magnitude in the study population.**	78%	50%	46%

Consensus in 1 group

- Discussion and voting

A. Include

B. Exclude

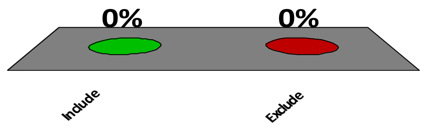

Discussion: Interpretation

Item	Journal Editors	Primary Researchers	Systematic Reviewers
**84. Give a cautious overall interpretation of results considering objectives, limitations, multiplicity of analyses, results from similar studies, and other relevant evidence.**	100%	90%	100%

Consensus in 3 groups

- Automatic inclusion

A. Include

B. Exclude

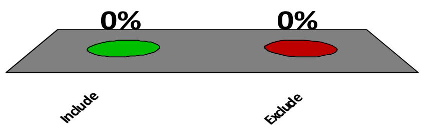

Discussion: Interpretation

Item	Journal Editors	Primary Researchers	Systematic Reviewers
**85. Report genotype frequencies from other studies.**	50%	23%	36%

Consensus in 0 groups

- Automatic exclusion

A. Include

B. Exclude

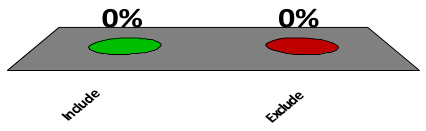

Discussion: Generalisability

Item	Journal Editors	Primary Researchers	Systematic Reviewers
**86. Discuss the generalisability (external validity) of the study results.**	90%	81%	73%

Consensus in 3 groups

- Automatic inclusion

A. Include

B. Exclude

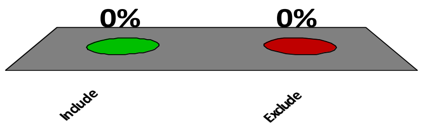

Discussion: Generalisability

Item	Journal Editors	Primary Researchers	Systematic Reviewers
**87. Discuss, if pertinent, the health care relevance of the study results.**	90%	67%	55%

Consensus in 1 group

- Discussion and voting

A. Include

B. Exclude

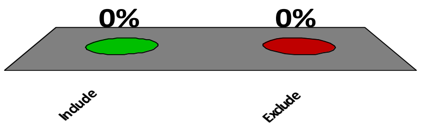

Other Information: Study Registration/Protocol

Item	Journal Editors	Primary Researchers	Systematic Reviewers
**88. State whether the protocol for the analysed data is publicly available and if so, how the protocol can be accessed.**	30%	58%	46%

Consensus in 0 groups

- Automatic exclusion

A. Include
B. Exclude

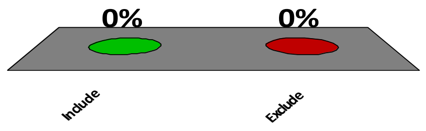

Other Information: Study Registration/Protocol

Item	Journal Editors	Primary Researchers	Systematic Reviewers
**89. State whether the study has been registered. If the study has been registered, provide details of the registry.**	80%	71%	36%

Consensus in 2 groups

- Discussion and voting

A. Include
B. Exclude

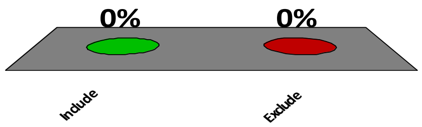

Other Information: Ethical Approval

Item	Journal Editors	Primary Researchers	Systematic Reviewers
**90a. Report whether ethical approval was obtained for the collection of genetic data.**	100%	97%	73%

Consensus in 3 groups

- Automatic inclusion

A. Include

B. Exclude

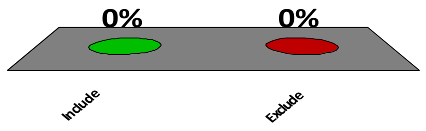

Other Information: Ethical Approval

Item	Journal Editors	Primary Researchers	Systematic Reviewers
**90b. If ethical approval was obtained, report the committee that gave ethical approval and a reference ID.**	50%	55%	27%

Consensus in 0 groups

- Automatic exclusion

A. Include

B. Exclude

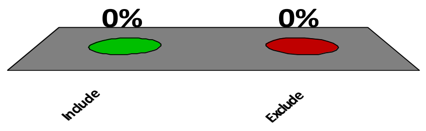

Other Information: Funding

Item	Journal Editors	Primary Researchers	Systematic Reviewers
**91. Give the source of funding and the role of the funders for the present study and, if applicable, for the original study on which the present article is based.**	80%	81%	82%

Consensus in 3 groups

- Automatic inclusion

A. Include

B. Exclude

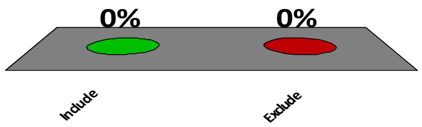

Other Information: Databases

Item	Journal Editors	Primary Researchers	Systematic Reviewers
**92. State whether databases for the analysed data are or will become publicly available and if so, how they can be accessed.**	70%	65%	46%

Consensus in 1 group

- Discussion and voting

A. Include

B. Exclude

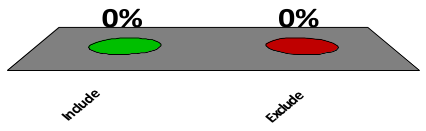

Groupings

- Large number of items
- Suggestions on how to group items are welcome
- Final guideline will be sent via e-mail for feedback
